# Role of the X Chromosome in Alzheimer Disease Genetics

**DOI:** 10.1001/jamaneurol.2024.2843

**Published:** 2024-09-09

**Authors:** Michael E. Belloy, Yann Le Guen, Ilaria Stewart, Kennedy Williams, Joachim Herz, Richard Sherva, Rui Zhang, Victoria Merritt, Matthew S. Panizzon, Richard L. Hauger, J. Michael Gaziano, Mark Logue, Valerio Napolioni, Michael D. Greicius

**Affiliations:** 1 Department of Neurology and Neurological Sciences, Stanford University School of Medicine, Stanford, California; 2 NeuroGenomics and Informatics Center, Washington University School of Medicine, St Louis, Missouri; 3 Department of Neurology, Washington University School of Medicine, St Louis, Missouri; 4 Quantitative Sciences Unit, Department of Medicine, Stanford University School of Medicine, Stanford, California; 5 Center for Translational Neurodegeneration Research, Department of Molecular Genetics University of Texas Southwestern Medical Center at Dallas, Dallas; 6 Biomedical Genetics, Boston University Chobanian & Avedisian School of Medicine, Boston, Massachusetts; 7 National Center for PTSD, Behavioral Sciences Division, VA Boston Healthcare System, Boston, Massachusetts; 8 Center of Excellence for Stress and Mental Health, VA San Diego Healthcare System, San Diego, California; 9 Department of Psychiatry, University of California San Diego, La Jolla; 10 Center for Behavior Genetics of Aging, University of California, San Diego, La Jolla; 11 Million Veteran Program (MVP) Coordinating Center, VA Boston Healthcare System, Boston, Massachusetts; 12 Division of Aging, Brigham & Women’s Hospital, Harvard Medical School, Boston, Massachusetts; 13 Department of Psychiatry, Boston University Chobanian & Avedisian School of Medicine, Boston, Massachusetts; 14 Department of Biostatistics, Boston University School of Public Health, Boston, Massachusetts; 15 School of Biosciences and Veterinary Medicine, University of Camerino, Camerino, Italy

## Abstract

**Importance:**

The X chromosome has remained enigmatic in Alzheimer disease (AD), yet it makes up 5% of the genome and carries a high proportion of genes expressed in the brain, making it particularly appealing as a potential source of unexplored genetic variation in AD.

**Objectives:**

To perform the first large-scale X chromosome–wide association study (XWAS) of AD.

**Design, Setting, and Participants:**

This was a meta-analysis of genetic association studies in case-control, family-based, population-based, and longitudinal AD-related cohorts from the US Alzheimer’s Disease Genetics Consortium, the Alzheimer’s Disease Sequencing Project, the UK Biobank, the Finnish health registry, and the US Million Veterans Program. Risk of AD was evaluated through case-control logistic regression analyses. Data were analyzed between January 2023 and March 2024. Genetic data available from high-density single-nucleotide variant microarrays and whole-genome sequencing and summary statistics for multitissue expression and protein quantitative trait loci available from published studies were included, enabling follow-up genetic colocalization analyses. A total of 1 629 863 eligible participants were selected from referred and volunteer samples, 477 596 of whom were excluded for analysis exclusion criteria. The number of participants who declined to participate in original studies was not available.

**Main Outcome and Measures:**

Risk of AD, reported as odds ratios (ORs) with 95% CIs. Associations were considered at X chromosome–wide (*P* < 1 × 10^−5^) and genome-wide (*P* < 5 × 10^−8^) significance. Primary analyses are nonstratified, while secondary analyses evaluate sex-stratified effects.

**Results:**

Analyses included 1 152 284 participants of non-Hispanic White, European ancestry (664 403 [57.7%] female and 487 881 [42.3%] male), including 138 558 individuals with AD. Six independent genetic loci passed X chromosome–wide significance, with 4 showing support for links between the genetic signal for AD and expression of nearby genes in brain and nonbrain tissues. One of these 4 loci passed conservative genome-wide significance, with its lead variant centered on an intron of *SLC9A7* (OR, 1.03; 95% CI, 1.02-1.04) and colocalization analyses prioritizing both the *SLC9A7* and nearby *CHST7* genes. Of these 6 loci, 4 displayed evidence for escape from X chromosome inactivation with regard to AD risk.

**Conclusion and Relevance:**

This large-scale XWAS of AD identified the novel *SLC9A7* locus. *SLC9A7* regulates pH homeostasis in Golgi secretory compartments and is anticipated to have downstream effects on amyloid β accumulation. Overall, this study advances our knowledge of AD genetics and may provide novel biological drug targets. The results further provide initial insights into elucidating the role of the X chromosome in sex-based differences in AD.

## Key Points

**Question:**

Does the X chromosome play a role in the genetics of Alzheimer disease (AD)?

**Findings:**

In a genetic meta-analysis across 1 152 284 individuals, several X chromosome loci were associated with AD. Four loci showed evidence of shared genetic associations between AD risk and regulation of nearby gene expression in brain tissue; the top association signal was intronic on *SLC9A7* and linked to its expression.

**Meaning:**

The results of this large-scale X chromosome–wide association study of AD prioritized *SLC9A7* as a novel risk locus, advancing our knowledge of AD genetics and providing novel biological drug targets.

## Teaser

This genetic association study evaluates the association between the X chromosome and Alzheimer disease.

## Introduction

Despite making up 5% of the genome, the X chromosome has remained enigmatic in the broader field of genome-wide association studies.^1^ It is typically excluded due to technical and analytical challenges.^1,2^ These include men having only 1 X chromosome (ie, hemizygous), while in women, 1 of 2 X chromosomes in each cell undergoes random X chromosome inactivation (XCI) to balance gene expression relative to men, complicating models and reducing statistical power in genetic association analyses.^1,2,3^ Additionally, approximately 30% of X chromosome genes show at least some level of escape from XCI, while XCI may additionally be nonrandom at the population level.^4,5^ In terms of quality control, the X chromosome requires sex-aware data processing and increased scrutiny due to larger intervariant linkage, decreased variant density and imputation quality on single-nucleotide variant (SNV) microarrays, challenges with variant calling in sequencing data, and several other factors.^1,2^

Nonetheless, there have been recent initial successes with large X chromosome–wide association studies (XWASs) in cardiovascular disease,^6,7^ kidney disease,^8^ and Parkinson disease,^9^ as well as other complex traits^3^ and brain imaging measures,^10,11^ revealing both sex-dependent and -independent associations. Yet, to our knowledge, no such XWAS has been conducted in AD, despite the X chromosome carrying a high proportion of genes that are expressed in the brain and relevant to intellectual disabilities.^11,12,13^ Furthermore, sex differences are pervasive in AD, having important impact on prevalence, pathobiology, and response to therapy,^14,15,16,17,18^ but how the X chromosome ties into this remains largely unknown. Interactions of the X chromosome with sex hormones can affect sex-specific gene expression and complex traits.^3,19^ Even independent of sex hormones, carrying 2 copies of the X chromosome has been shown to contribute to resilience to AD^20^ and appears to be relevant to other traits as well.^21^ These observations are likely driven by genes escaping XCI in women (eg, *KDM6A*^20^), leading to higher expression levels in women that in turn can contribute to sex differences in disease phenotypes.^3,21^

We thus set out to fill in this gap in the AD field by performing the first meta-analysis of XWASs conducted on various publicly available AD-related cohorts as well as multiple biobanks where AD phenotypes were available. To ensure maximal power, this study was designed as a large-scale discovery combining all available samples. We additionally sought to interrogate sex-specific associations and potential XCI escape.

## Methods

An in-depth overview of all methodologies is provided in the eMethods in Supplement 1. The current study followed the Strengthening the Reporting of Genetic Association Studies (STREGA) reporting guideline. Participants or their caregivers provided written informed consent in the original studies. The current study protocol was granted an exemption by the Stanford University institutional review board because the analyses were carried out on deidentified, off-the-shelf data; therefore, additional informed consent was not required.

### Data Ascertainment

Case-control, family-based, and longitudinal AD genetic cohorts from the Alzheimer’s Disease Genetics Consortium (ADGC) and Alzheimer’s Disease Sequencing Project (ADSP) (release 3) were available through public repositories with genetic data from SNV microarrays and whole-genome sequencing (eTable 1 and 2 in Supplement 1).^22,23^ These cohorts contributed clinically diagnosed AD cases and have similar sample demographic characteristics (40.0% pathology verified; eTable 3 in Supplement 1).^24,25,26^ Analyses in the UK Biobank (UKB), Finnish health registry (FinnGen), and US Million Veterans Program (MVP) used genetic data from SNV microarrays.^27,28,29,30^ UKB data and FinnGen summary results (version 10) were publicly available. For FinnGen, to maximize power, we made use of the broad AD definition phenotype without age filters for control individuals, consistent with prior AD autosomal association studies.^31^ UKB contributed health registry–confirmed AD cases and proxy Alzheimer disease and dementia (ADD) cases; FinnGen contributed health registry–confirmed AD cases; and MVP contributed health registry–confirmed (MVP-1) and proxy ADD cases (MVP-2). Data were analyzed between January 2023 and March 2024.

### Quality Control and Processing

ADGC and ADSP data underwent extensive quality control and imputation to the Trans-Omics for Precision Medicine reference panel (eTable 4 and 5 in Supplement 1). Specific consideration was given to X chromosome quality control, as in prior work (eMethods in Supplement 1).^9^ Genetic data processing for UKB, FinnGen, and MVP followed cohort-specific protocols.^27,28,29,30^ Non-Hispanic White, European ancestry individuals with AD and control individuals, carrying XX or XY with concordant self-reported sex and ages older than 60 years (older than 18 years and a median of 63 years in FinnGen), were retained for analyses (eFigure 1, eTable 3 in Supplement 1). Variants were filtered using cohort-specific minor allele frequency criteria, which on average correspond to a minor allele frequency greater than 0.05% (eTable 5 in Supplement 1).

### X Chromosome Considerations

X chromosome analyses considered nonpseudoautosomal regions (pseudoautosomal regions were not evaluated due to insufficient variant coverage across ADGC SNV microarrays). Analyses evaluated a model of random XCI, for which genotype encoding was 0/2 in men (XY) and 0/1/2 in women (XX). This considers that in women, a single genotype only represents a 50% probability of association due to random XCI. When men and women are combined into 1 analysis, the genotype effect for men has to be placed on the same scale as for women, which is achieved by treating the male genotype as biallelic (0/2) making a single genotype correspond to a 50% probability of association.^32^ In UKB, most cases were proxy cases (ie, family history of ADD in first-degree relatives). This proxy approach has been established to replicate AD autosomal genetic risk factors and be adaptable to XWASs.^9,33^ To maximize power, the health registry and proxy status were unified into a single phenotype, following a model for random XCI, for which association coefficients were adjusted onto a regular case-control scale (eTables 6 and 7 in Supplement 1). After rescaling, UKB showed consistent coefficient distributions with ADGC and ADSP (eFigure 2 in Supplement 1). A similar approach was used in MVP, but in line with MVP protocols, analyses were separated for health registry and proxy phenotypes.^29^

A typical model to evaluate XCI escape would set genotype encoding to 0/1 in men (XY) and 0/1/2 in women (XX).^32^ However, given the use of tools for mixed-model genetic association analyses (to boost power) and partial reliance on external summary statistics, it was not possible to implement this approach across cohorts. Instead, evidence for escape from XCI was evaluated by comparing variant β coefficients derived from male- and female-stratified XWASs, using a similar approach as described in Sidorenko et al.^3^ In this comparison, male β coefficients were rescaled to represent a 100% probability of association. The assumption is that under no escape from XCI in women (where a single genotype confers 50% probability of association), male β coefficients should be approximately twice as large compared to those in women (ratio closer to 2). Under escape from XCI in women (where a single genotype confers 100% probability of association), male and female β coefficients should be approximately consistent (ratio closer to 1).

### Statistical Analyses

XWASs evaluated case-control logistic regressions on AD risk, adjusting for sex, age, technical covariates, and genetic principal components (capturing population stratification) as applicable per dataset. Mixed models to include related participants were used in ADGC, ADSP, UKB (BOLT-LMM version 2.4),^35^ and FinnGen (Regenie).^28^ Association results across datasets were combined through fixed-effects inverse variance–weighted meta-analyses, which is the standard approach for within ancestry genome-wide meta-analyses in AD.^31,36^ To increase specificity to AD (rather than ADD), the XWAS meta-analysis was intersected to variants with association results in ADGC. Primary analyses were nonstratified. Secondary analyses were sex stratified and conducted across ADGC, ADSP, UKB, MVP-1, and MVP-2 (sex-stratified results were not available for FinnGen). Sensitivity analyses were conducted to meta-analyze all cohorts excluding those with proxy phenotypes (UKB and MVP-2). This was not pursued for the secondary sex-stratified analyses because MVP-1 is heavily skewed toward male participants, which would preclude a balanced male to female comparison. Association results were considered at the X chromosome–wide (*P* < 1 × 10^−5^) and conservative genome-wide thresholds (*P* < 5 × 10^−8^). In addition to evaluating escape from XCI, sex effects were further evaluated through heterogeneity tests and considered significant at *P* < .05. In these tests, male β coefficients were scaled to correspond to genotype encoding 0/2 (rather than 0/1 when evaluating XCI escape). This implies that sex heterogeneity is nonsignificant when no escape from XCI is apparent (ratio = 2), and becomes more significant with apparent escape (ratio = 1-2) or further sex specificity (ratio >2 or ratio <1). Lastly, the effective sample size of the AD XWAS was estimated using the formulation effective sample size = 4 × v × (1 − v), where v = the number of cases / (number of cases + the number of controls).^37^ This measure makes sample sizes and power estimation more comparable across different case-control genome-wide association studies, by converting a respective study’s sample size to that for an equivalently powered study with a balanced sample design (50/50 cases and controls).^37,38^

Independent lead variants were annotated with the nearest protein-coding gene, which was used to refer to the respective loci regardless of functional mapping results. To identify potentially causal genes in associated risk loci, statistical colocalization was evaluated between the local genetic association signal for AD and the genetic association signal for molecular traits, such as expression levels of genes within that locus (R version 4.2.1 [R Foundation], coloc package).^34^ We leveraged public datasets where quantitative trait loci for expression and protein levels were available for the X chromosome in brain and nonbrain tissues (eMethods in Supplement 1).

## Results

**Figure 1.**
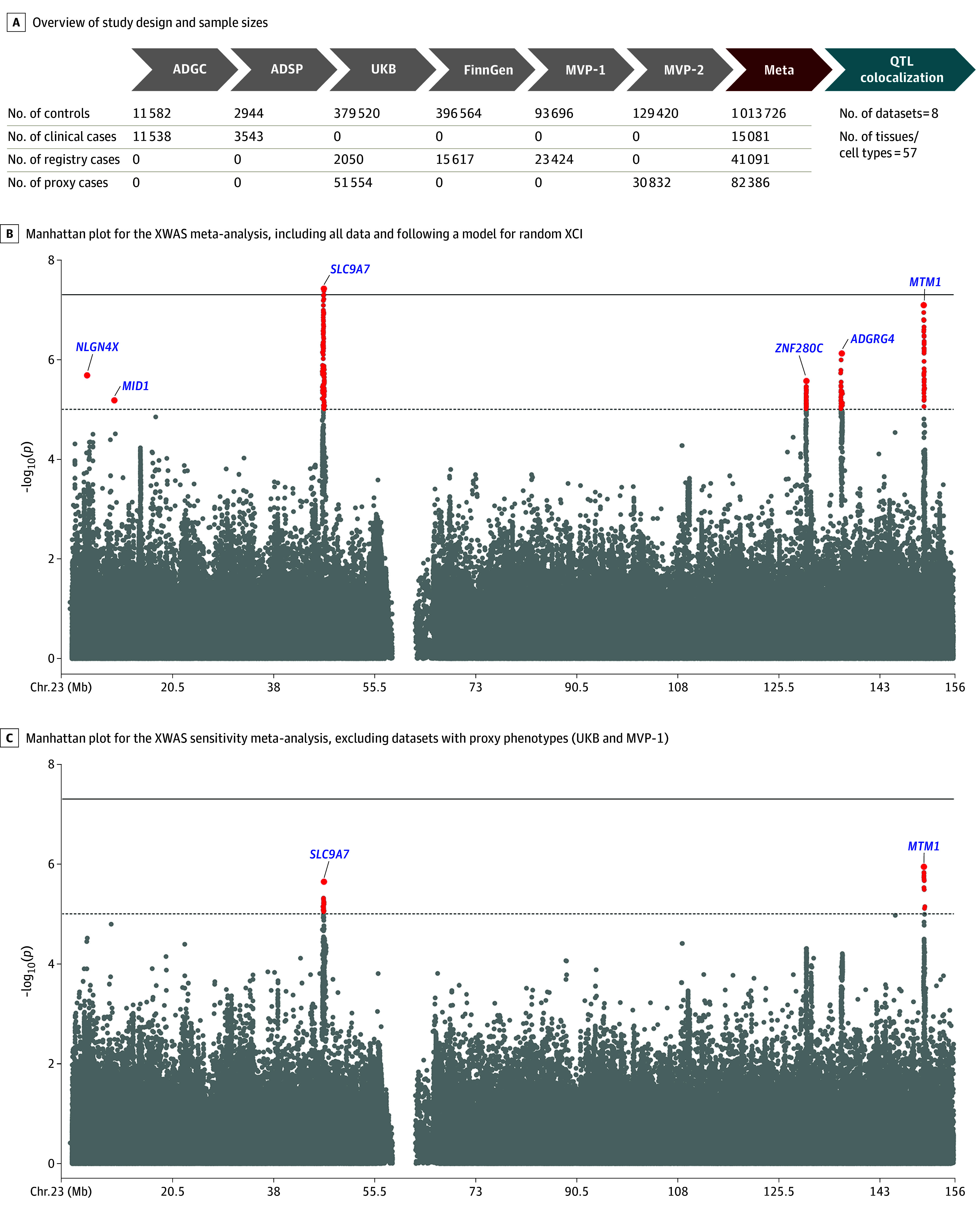
X Chromosome–Wide Association Study (XWAS) of Alzheimer Disease (AD) A, To increase specificity to AD (rather than AD with dementia), the XWAS meta-analysis was intersected to variants with association results in the ADGC, which used only clinically confirmed cases and controls. B and C, The dotted lines indicate X chromosome–wide significance (*P* < 1 × 10^−5^) and full lines indicate genome-wide significance (*P* < 5 × 10^−8^). Lead variants for independent loci are annotated with their nearest protein-coding gene (GENCODE version 42), which does not reflect functional mapping. Pseudo-autosomal regions were not included in the analyses and are thus not depicted. ADCG indicates Alzheimer’s Disease Genetics Consortium; ADSP, Alzheimer’s Disease Sequencing Project; meta, meta-analysis; MVP, Million Veteran Program; QTL, quantitative trait loci; UKB, UK Biobank; XCI, X chromosome inactivation.

The study design is provided in Figure 1A. A total of 1 152 284 individuals (664 403 [57.7%] female and 487 881 [42.3%] male; 138 558 with AD [15 081 clinically diagnosed and 41 091 health registry confirmed] and 82 386 proxy individuals) were included in the XWAS (eTable 3 in Supplement 1), with an estimated effective sample size of 487 588. There was no sign of genomic inflation (eFigure 3 in Supplement 1). Using all data, we associated 2 rare (minor allele frequency less than 1%) lead variants in the *NLGN4X* and *MID1* loci and 4 common lead variants in the *SLC9A7*, *ZNF280C*, *ARGRG4*, and *MTM1* loci (Figure 1B, Table 1; locus zoom and forest plots in eFigures 4 and 5 in Supplement 1). All common variant loci showed colocalization for at least 1 nearby gene in brain tissue (Table 2^39,40^; eTable 8 in Supplement 1). The overall top association signal (cross-cohort allele frequencies in eTable 9 in Supplement 1), intronic on *SLC9A7*, passed conservative significance criteria (OR, 1.03; 95% CI, 1.02-1.04) and showed colocalization for several genes, most notably *SLC9A7* and *CHST7*. Colocalization plots for top prioritized genes are in eFigures 6-10 in Supplement 1. In sensitivity analyses excluding data with proxy phenotypes, we again identified the *SLC9A7* and *MTM1* loci at X chromosome–wide significance (Figure 1C). These signals were not independent of those in the primary analysis. The 6 lead variants from the primary analyses displayed highly consistent effect sizes in the sensitivity analyses (Table 1).

**Table 1.** Associated Lead Variants in the X Chromosome-Wide Association Study (XWAS) of Alzheimer Disease (AD)

Lead variant^a^	Nearest protein coding gene	Result	BP	EA	OA	Analysis	No. of individuals	EAF, %	OR (95%-CI)^b^	*P* value^c^	Direction^d^					
rs150798997	*NLGN4X*	Intergenic	5 733 126	A	T	All data	1 145 553	0.32	0.80 (0.73-0.88)	2.08 × 10^−6^	−	NA	−	−	−	−
						No proxy data	552 177	0.29	0.80 (0.73-0.89)	3.06 × 10^−5^	−	NA	NR	−	−	NR
rs12852495	*MID1*	Intronic	10 458 864	T	C	All data	1 151 353	0.26	1.24 (1.13-1.36)	6.60 × 10^−6^	+	+	+	+	+	+
						No proxy data	557 977	0.21	1.18 (1.06-1.31)	3.22 × 10^−3^	+	+	NR	+	+	NR
rs2142791^e^	*SLC9A7*	Intronic	46 691 127	C	A	All data	1 152 185	46.12	1.03 (1.02-1.04)	3.78 × 10^−8^	+	+	+	+	+	+
						No proxy data	558 809	46.39	1.03 (1.01-1.04)	6.38 × 10^−6^	+	+	NR	+	+	NR
rs209215	*ZNF280C*	Intronic	130 251 839	T	C	All data	1 145 797	39.90	1.02 (1.01-1.03)	2.70 × 10^−6^	+	NA	+	+	+	+
						No proxy data	552 421	40.80	1.02 (1.01-1.04)	6.38 × 10^−5^	+	NA	NR	+	+	NR
rs5975709^f^	*MAP7D3*	Intronic	136 256 153	C	T	All data	1 145 797	43.25	0.98 (0.97-0.99)	1.02 × 10^−6^	−	NA	−	−	−	−
						No proxy data	552 421	40.90	0.98 (0.97-0.99)	1.15 × 10^−4^	−	NA	NR	−	−	NR
rs5930938	*ADGRG4*	Intronic	136 380 525	T	C	All data	733 616	32.62	0.97 (0.96-0.98)	7.55 × 10^−7^	−	NA	−	NA	−	−
						No proxy data	140 240	32.03	0.97 (0.96-0.99)	2.15 × 10^−4^	−	NA	NR	NA	−	NR
rs146964414	*MTM1*	Intronic	150 608 170	T	C	All data	1 152 184	8.23	1.05 (1.03-1.07)	8.10 × 10^−8^	+	−	+	+	+	+
						No proxy data	558 808	8.48	1.05 (1.03-1.07)	1.14 × 10^−6^	+	−	NR	+	+	NR

Abbreviations: BP, base pair; EA, effect allele; EAF, effect allele frequency; NA, not available; NR, not reported (omitted to avoid inclusion of proxy samples in sensitivity analyses); OA, other allele; OR, odds ratio.

^a^ Variants are annotated using dbSNP153.

^b^ Because the XWAS followed a model for random X chromosome inactivation (XCI), the reported ORs correspond to a 50% probability of the allele being active. Assuming no escape from XCI, the effect size for a single allele would thus be anticipated to be twice as large in men. Evaluation of evidence for escape from XCI and sex-specific effect sizes are provided in Figure 3 and eTable 10 in Supplement 1.

^c^ All reported variant meta-analyses were nonsignificant (*P* > .05) on the Cochran *Q* tests for effect heterogeneity.

^d^ The direction column indicates the association effect direction across meta-analyzed cohorts following the order of the Alzheimer’s Disease Genetics Consortium, the Alzheimer’s Disease Sequencing Project, UK Biobank, FinnGen, and Million Veteran Program 1 (using health registry status) and 2 (using proxy status).

^e^ Association signal passed genome-wide significance.

^f^ The lead variant in its respective locus, rs5975709, had no association results in the Alzheimer’s Disease Sequencing Project ADSP or FinnGen. The second most significant variant in this locus, rs5930938, did have association results in FinnGen and was therefore listed to provide additional insight.

**Table 2.** Genetic Colocalization With Quantitative Trait Locus Data^a^

Nonoverlapping datasets	Wingo et al, 2023 (AD Knowledge Portal)^39^		CommonMind (eQTL Catalogue)^40^	GTEx^41^			Fairfax et al, 2014 (eQTL Catalogue)^40^	CEDAR (eQTL Catalogue)^40^	No. of times prioritized across datasets and tissues^b^^,^^c^	No. of times prioritized in nonoverlapping datasets^b^^,^^c^^,^^d^
Tissue	Brain: dorsolateral prefrontal cortex		Brain: dorsolateral prefrontal cortex	Brain: 9 areas	Whole blood	Other (20 tissues not brain or blood)	Monocytes: 4 conditions	Monocytes	NA	NA
QTL	pQTL	eQTL	eQTL	eQTL	eQTL	eQTL	eQTL	eQTL	NA	NA
*SLC9A7*										
* ENSG00000286306*	NA	NA	0.88^c^	NA	NA	NA	NA	NA	1	1
* KRBOX4*	NA	0.77^c^^,^^e^	0.04	0.74^c^	0.23	0.92^c^	0.10	0.05	4	2
* CHST7b*	NA	0.95^c^^,^^e^	0.92^c^^,^^e^	0.86^c^^,^^e^	0.20	0.71^c^	0.27	0.15	10	3
* SLC9A7b*	0.56	0.56	0.86^c^^,^^e^	0.64	0.39	0.98^c^^,^^e^	0.93^c^^,^^e^	0.93^c^^,^^e^	12	4
* RP2*	0.89^c^^,^^e^	0.04	0.07	0.13	0.03	0.14	0.50	0.05	1	1
* JADE3*	NA	0.70^c^^,^^e^	0.07	0.51	0.06	0.76^c^	0.10	0.43	2	2
* UBA1*	0.05	0.04	0.93^c^^,^^e^	0.12	0.07	0.10	0.13	0.12	1	1
* ELK1*	NA	0.00	0.04	0.33	0.03	0.16	0.86^c^	0.08	1	1
*ZNF280C*										
* ELF4*	NA	NA	0.05	0.11	0.02	0.78^c^^,^^e^	0.01	0.06	1	1
* AIFM1*	0.02	0.16	0.12	0.56	0.19	0.78^c^^,^^e^	0.73^c^	0.78^c^^,^^e^	6	3
* ZNF280Cb*	NA	0.45	0.09	0.77^c^^,^^e^	0.05	0.92^c^^,^^e^	0.19	0.04	17	1
* RBMX2*	NA	0.00	0.29	0.07	0.02	0.77^c^	0.04	0.05	1	1
*ADGRG4*										
* FHL1*	0.12	0.78^c^^,^^e^	0.13	0.10	0.02	0.13	0.85^c^	0.29	2	2
* MAP7D3b*	0.05	0.93^c^^,^^e^	0.92^c^^,^^e^	0.77^c^^,^^e^	0.92^e^	0.89^c^^,^^e^	0.13	0.08	10	3
* BRS3*	NA	NA	0.04	0.04	NA	0.73^c^	0.04	0.04	1	1
* HTATSF1*	0.03	0.05	0.03	0.04	0.01	0.87^c^^,^^e^	0.07	0.06	2	1
* AL683813.2*	NA	NA	NA	0.07	0.02	0.77^c^	NA	NA	1	1
*MTM1*										
* MTMR1b*	0.01	0.05	0.16	0.88^c^	0.01	0.79^c^	0.18	0.11	2	1

Abbreviations: eQTL, expression quantitative trait locus; NA, not available; pQTL, protein quantitative trait locus; QTL, quantitative trait locus.

^a^ Colocalization was evaluated for genes in each AD-associated locus using a 2Mb-window centered on the lead variant. Evidence for colocalization was considered at colocalization posterior probability (PP4) > 0.7. The table presents PP4 results and is restricted to genes and datasets or tissues where at least 1 colocalization reached PP4 > 0.7. As such, the table is partitioned into 4 common variant loci that showed colocalization support.

^b^ The total number of times a gene was prioritized (PP4 > 0.7) is summarized to help identify the most likely causal gene per locus.

^c^ PP4 > 0.7.

^d^ Overlapping datasets were considered as those where individuals partially or fully overlapped.

^e^ The lead variant was also a significant QTL in the respective data or tissue.

**Figure 2.**
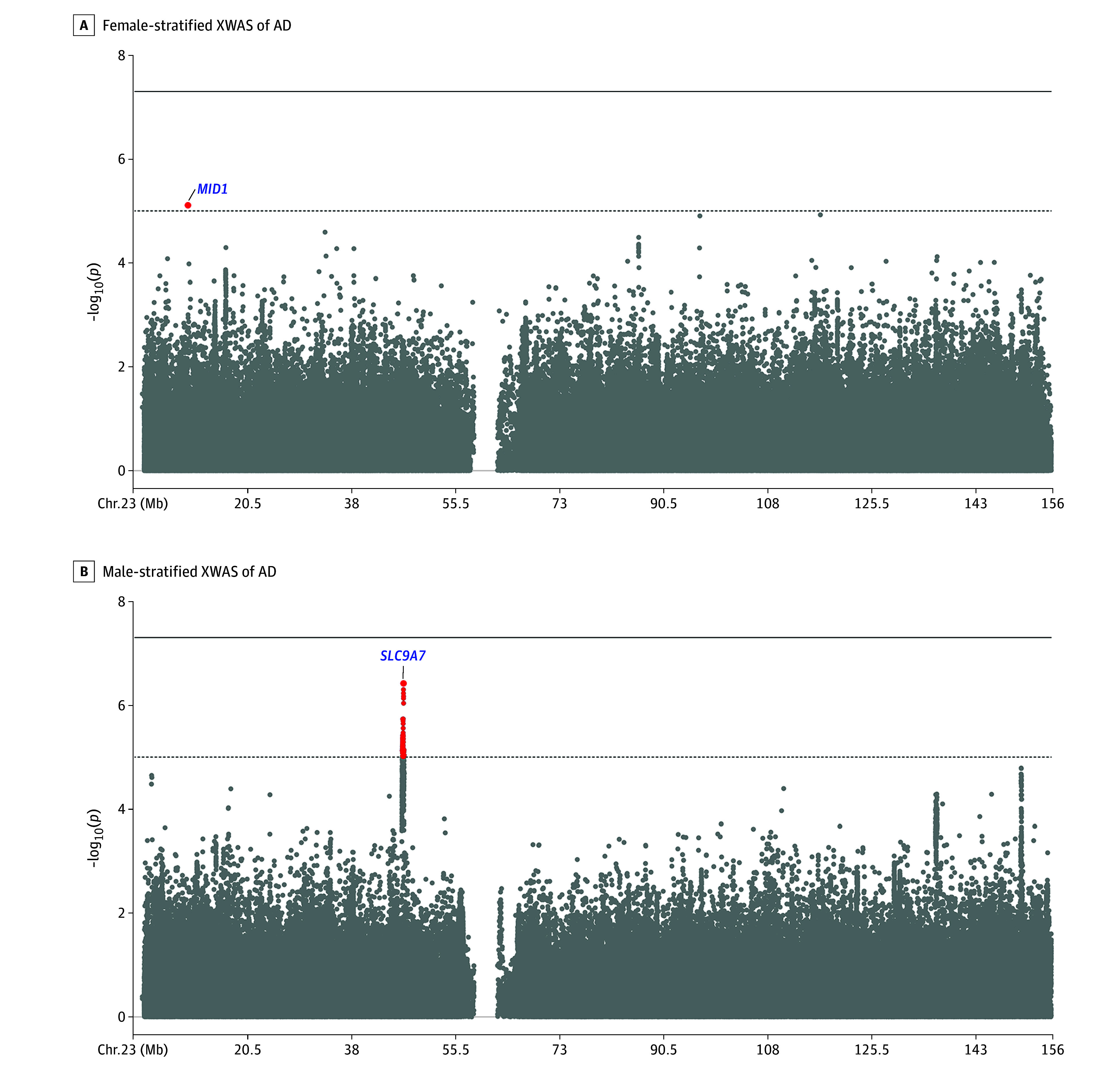
Sex-Stratified X Chromosome–Wide Association Studies (XWASs) of Alzheimer Disease (AD) A and B, Each respective sex-stratified XWAS displayed a single independent association signal that passed the X chromosome–wide significance (*P* < 1 × 10^−5^; dotted line), but these were consistent with those observed in the nonstratified XWAS (Figure 1B). None passed the genome-wide significance threshold (*P* < 5 × 10^−8^; solid line).

**Figure 3.**
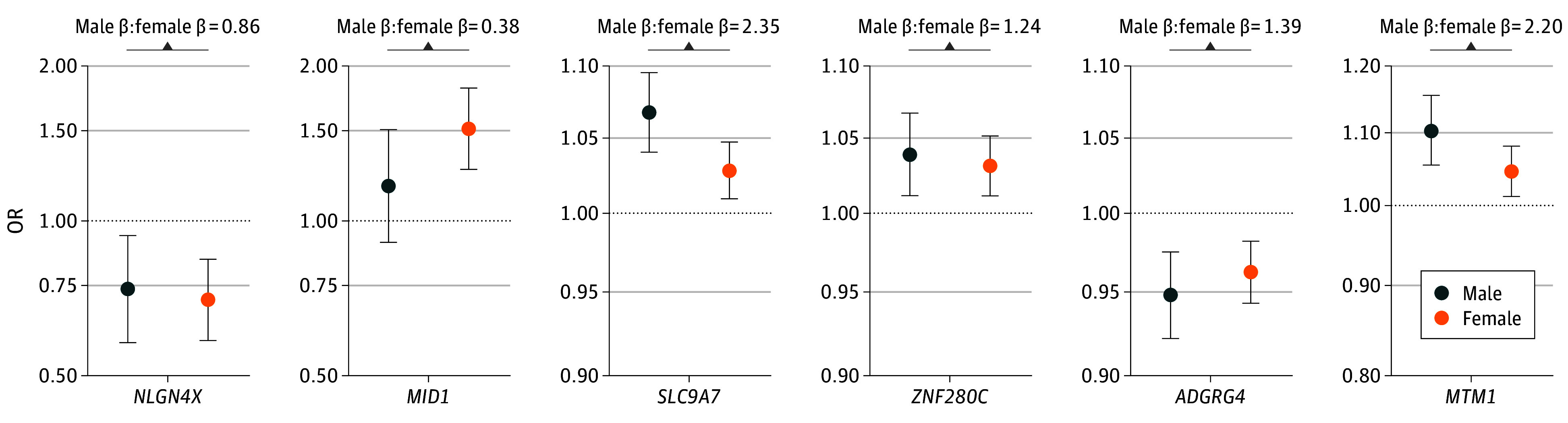
Evaluation of Escape From X Chromosome Inactivation (XCI) All nonstratified lead variants for the 6 independent risk loci are shown reporting their sex-specific odds ratios (ORs), as well as the ratio of male to female β coefficients to assess evidence for escape from XCI (eTable 10 in Supplement 1). The male β coefficients here correspond to a 100% probability of association of the genotype. Ratios close to 2 suggest no escape from XCI, while ratios closer to 1 suggest escape from XCI.

Sex-stratified XWASs showed the *SLC9A7* locus in men and the *MID1* locus in women passing X chromosome–wide significance criteria (Figure 2A and B). These signals were not independent of those in the nonstratified analyses, meaning no additional sex-stratified signals were observed. When evaluating lead variants from the nonstratified analyses for evidence of escape from XCI with regard to AD risk (Figure 3; eTable 10 in Supplement 1), escape was not apparent for the *SLC9A7* and *MTM1* lead variants but was apparent for *NLGN4X*, *MID1*, *ZNF280C*, and *ARGRG4*. The *MID1* lead variant furthermore displayed a significant female-biased heterogeneity effect (eTable 10 in Supplement 1). Additional evaluation of XCI escape, including subthreshold signals from male- and female-stratified XWASs, further supported apparent escape from XCI for some common, small effect size variants (eFigure 11 in Supplement 1).

## Discussion

We performed an XWAS of AD in 1 152 284 individuals, making this the largest genetic association study of AD to date.^38^ The top signal showed support for a link between the genetic regulation of *SLC9A7* or *CHST7* expression and AD risk. *CHST7* encodes a chondroitin 6-sulfotransferase that confers negatively charged sulfate groups to glycosaminoglycans, which may relate to promoting tau fibrillization and spreading.^42^ Notably, *SLC9A7* (also known as *NHE7*) is a paralog of *SLC9A6* (also known as *NHE6*), previously implicated in experimental work as an X-linked AD-modifying gene.^43^ It is further notable that *SLC9A6* is only 209Kb away from the *ADGRG4* lead variant. Although no colocalization support was observed for *SLC9A6* at this locus, it may still be linked to the AD association signal through other mechanisms that we could not explore here. *SLC9A7* and *SLC9A6* are highly conserved genes that regulate pH homeostasis in Golgi secretory compartments and endosomes and might thus be expected to contribute to increased amyloid accumulation across aging when their expression levels are increased (a potential mechanistic hypothesis is provided in the eDiscussion in Supplement 1). In line with this expectation, quantitative trait loci data support that the top risk allele in the *SLC9A7* locus was associated with increased expression of *SLC9A7* in brain tissue, increasing expression by 8% to 22% under an XCI model (eTable 11 in Supplement 1). Although the *SLC9A7* top variant has a small effect size under the XCI model (OR, 1.03; 95% CI, 1.02-1.04), it is important to note that genetic effect size on disease risk has no impact on the predictive value of genetic evidence in drug trial success.^44^ This relates to the fact that small effects on disease risk may be conferred by small effects on gene regulation, while a drug may have more profound impact on gene regulation and in turn disease pathogenesis. It is also relevant that genetic variation on *SLC9A7* has been shown to be associated with intellectual disability,^45^ such that variants with strong regulatory effects may in fact be selected against and thus not observed in an AD XWAS. Given the relatively small effect of the lead variant on *SLC9A7* expression in the brain, it may thus be that more substantial reduction or pharmacological inhibition of *SLC9A7* would prove to be an effective therapeutic strategy for AD.

Despite this study’s formidable sample size, only the *SLC9A7* locus reached conservative significance criteria. When comparing to the latest AD autosomal meta-analysis, we might have expected to observe approximately 4 independent genome-wide significant hits considering the size of the X chromosome or its number of protein-coding genes. This discrepancy may in part be due to the inherent technical challenges on X chromosome compared to autosomes that could have led to increased false negatives.^1,2^ Further, even when the X chromosome is included in genetic associations studies, it has been shown to contribute relatively fewer significant variants than the autosomes.^2^ This is thought to be due to the fact that the X chromosome is less tolerant of deleterious variations and has fewer functional variants than the autosomes as further reflected by the finding that the proportion of genic variants on X is smaller than on autosomes, whereas the proportion of intergenic variants is similar.^2^ Perhaps more importantly, the effective sample size and power of our AD XWAS is likely overestimated. The prior autosomal study in AD with the largest effective sample size had an effective sample size of 382 470,^38^ whereas ours had an effective sample size of 487 588. However, when taking into account reduced effective sample sizes when using proxy phenotypes,^46^ these numbers would be closer to approximate effective sample sizes of 258 364 and 273 815, respectively, suggesting no striking increase in power in our XWAS. Furthermore, statistical power for a genetic variant is correlated with the amount of phenotypic variance that variant can explain.^47^ Compared to autosomal analyses, male individuals are hemizygous for nonpseudoautosomal regions on the X chromosome, which constitutes a 50% reduction in how much phenotypic variance a variant can explain.^3^ In women, when there is random XCI, the impact is even more pronounced with a 75% reduction, while in the case of XCI escape there is no reduction.^3^ Indeed, prior XWASs of complex traits and brain phenotypes confirm that under comparable sample sizes, male XWASs identify substantially more loci than female XWASs,^3,10^ consistent with our observation of the *SLC9A7* locus in the male-stratified AD XWAS and it not appearing to escape XCI in women. It is therefore reasonable to consider that the effective sample in our AD XWAS is likely further reduced by approximately 50%. Combined with the other factors detailed above, this helps explain why only 1 locus in our study passed conventional significance criteria and highlights the power challenges faced when studying X chromosome genetics in AD.

Even when considering power challenges, the current findings primarily identified low-impact common variants, both in nonstratified and sex-stratified analyses, suggesting a modest role of X chromosome genetics in AD prevalence. This would be in line with less pronounced sex differences in AD prevalence after accounting for female survival bias.^48^ Nonetheless, sex-stratified analyses identified 4 loci appearing to escape XCI. These may tie into observations such as women appearing to show increased AD incidence at advanced ages^48^ and increased tau burden in the AD pathologic trajectory.^49^ It may also be that these loci are relevant to resilience to AD rather than AD directly, such that they may relate to women appearing to survive longer with AD (figure 1 in Davis et al^20^), having more preserved brain structure despite elevated tau burden,^50^ and higher baseline cognitive reserve but reduced coping with AD pathology and more rapid cognitive decline across aging and AD^51,52,53^ (see Arenaza-Urquijo et al^53^ for a comprehensive review of sex differences in resilience or vulnerability to aging and AD). Further, the *MID1* locus notably showed a significant female-biased association in addition to XCI escape, potentially indicating a hormone-related effect.^3^ Indeed, prior studies suggest a link between *MID1* and androgen receptor levels.^54,55^ Altogether, these results suggest that X chromosome genetics likely play a role in AD sex differences and warrant further investigation, opening the door to sex-specific pathogenic pathways and associated drug targets.

### Future Perspectives

It will be an important future research avenue to expand on sex-stratified XWASs to better elucidate the role of the X chromosome in sex-related differences in AD prevalence and pathogenesis. In light of promising initial evidence of X chromosome genetic and gene expression associations with AD biomarkers and pathology,^56,57^ as well as observations of sex differences in tau pathology and the genetics of tau pathology,^16,49,58,59^ there is, additionally, a need to more broadly explore the role of X chromosome genetics in AD pathogenesis rather than just AD risk. Furthermore, there is extensive evidence for a sex-specific effect of the APOE*4 allele on AD risk and pathology,^60,61,62^ but how the X chromosome ties into this remains to be determined. Addressing these research questions is likely to expand on the insights gained here. Furthermore, relevant risk genes may escape detection in an XWAS design, especially if the X chromosome is less tolerant of deleterious variations.^2^ Therefore, exploring the specific role of rare genetic variation,^63^ studying the impact of epigenetic alterations,^64,65,66^ and leveraging omics resources to profile molecular risk factors^39,56^ represent valuable complementary lines of research. Importantly, the results obtained in the current study should serve as an anchor point for future X chromosome studies in AD.

### Limitations

This study did not provide conclusive insight into the causal gene at the *SLC9A7* locus, which future experimental studies should interrogate. Further, the current study focused on individuals of European ancestry, limiting generalizability of the findings across populations. When larger cross-ancestry samples become available, future studies should extend AD XWASs into these populations. Additionally, findings from the current XWAS may be less specific to AD and may instead have been prone to capture associations with ADD, given the large fraction of proxy and registry-confirmed cases. The addition of more clinically confirmed cases and controls in future AD XWASs should help corroborate the current findings and reveal associations that may have been missed here. Under the current study design, the evaluation of escape from XCI was limited to comparing effect estimates from male vs female AD XWASs. Additionally, sex-specific effect estimates may have suffered from reduced accuracy by using proxy phenotypes, but those samples were needed to attain sufficient sample sizes and power. Future, larger sex-stratified AD XWASs will therefore be relevant to validate the current genes escaping XCI, as well as to identify additional ones.

## Conclusions

We performed the first large-scale XWAS of AD and identified the novel *SLC9A7* risk locus. Overall, this study advances our knowledge of the genetics of AD and may provide novel biological drug targets. In addition, our results provide initial insights into the role of the X chromosome in sex-related differences in AD prevalence and pathogenesis.

## Notes

Supplement 1. eMethods eDiscussion eFigure 1. Admixture plot across the five major super populations, for European ancestry case-control participants included in ADGC and ADSP eFigure 2. UKB beta coefficient adjustment onto a regular case-control scale eFigure 3. QQplot for the non-stratified AD XWAS meta-analysis including all data eFigure 4. Forest plots for all 6 lead variants from the non-stratified AD XWAS eFigure 5. Forest plots for all 6 lead variants from the non-stratified AD XWAS and locus zoom plots for all 6 lead variants from the non-stratified AD XWAS eFigure 6. CHST7 Colocalization plots eFigure 7. SLC9A7 colocalization plots and SLC9A7 colocalization plots eFigure 8. SLC9A7 colocalization plots eFigure 9. MAP7D3 colocalization plots and MAP7D3 colocalization plots eFigure 10. MTMR1 colocalization plots eFigure 11. Evaluation of escape from XCI in sex-stratified AD XWAS eTable 1. Overview of genotyping platforms across all available AD-related genetic data eTable 2. Overview of ADSP available through NIAGADS DSS (NG00067) eTable 3. Overview of participant demographics eTable 4. Overview of variant counts in ADGC cohorts with SNP arrays eTable 5. Overview of variant counts across datasets after quality control and intersection with ADGC eTable 6. Phenotype scoring and rescaling approach for the UKB non-stratified AD XWAS for a random XCI model eTable 7. Phenotype scoring and rescaling approach for the UKB sex-stratified AD XWAS eTable 8. Genetic colocalization with quantitative trait locus data: Extension of Table 2 without collapsing results from overlapping tissues eTable 9. Frequency in cases and controls for SLC9A7 lead variant across cohorts eTable 10. Evaluation of sex-specific effects and escape from X chromosome inactivation (XCI) for lead variants from non-stratified AD XWAS eTable 11. Effect sizes for SLC9A7 lead variant on SLC9A7 expression in brain tissue eReferences eAppendix. Additional contributions

Supplement 2. Data sharing statement
