# Supplementary material for: Role of the X Chromosome in Alzheimer Disease Genetics
Source: JAMA Neurol. 2024 Sep 9;81(10):1032–42. doi: 10.1001/jamaneurol.2024.2843 (PMC11385320; doi:10.1001/jamaneurol.2024.2843)
Supplement: Supplement 1. — eMethods eDiscussion eFigure 1. Admixture plot across the five major super populations, for European ancestry case-control participants included in ADGC and ADSP eFigure 2. UKB beta coefficient adjustment onto a regular case-control scale eFigure 3. QQplot for the non-stratified AD XWAS meta-analysis including all data eFigure 4. Forest plots for all 6 lead variants from the non-stratified AD XWAS eFigure 5. Forest plots for all 6 lead variants from the non-stratified AD XWAS and locus zoom plots for all 6 lead variants from the non-stratified AD XWAS eFigure 6. CHST7 Colocalization plots eFigure 7. SLC9A7 colocalization plots and SLC9A7 colocalization plots eFigure 8. SLC9A7 colocalization plots eFigure 9. MAP7D3 colocalization plots and MAP7D3 colocalization plots eFigure 10. MTMR1 colocalization plots eFigure 11. Evaluation of escape from XCI in sex-stratified AD XWAS eTable 1. Overview of genotyping platforms across all available AD-related genetic data eTable 2. Overview of ADSP available through NIAGADS DSS (NG00067) eTable 3. Overview of participant demographics eTable 4. Overview of variant counts in ADGC cohorts with SNP arrays eTable 5. Overview of variant counts across datasets after quality control and intersection with ADGC eTable 6. Phenotype scoring and rescaling approach for the UKB non-stratified AD XWAS for a random XCI model eTable 7. Phenotype scoring and rescaling approach for the UKB sex-stratified AD XWAS eTable 8. Genetic colocalization with quantitative trait locus data: Extension of Table 2 without collapsing results from overlapping tissues eTable 9. Frequency in cases and controls for SLC9A7 lead variant across cohorts eTable 10. Evaluation of sex-specific effects and escape from X chromosome inactivation (XCI) for lead variants from non-stratified AD XWAS eTable 11. Effect sizes for SLC9A7 lead variant on SLC9A7 expression in brain tissue eReferences eAppendix. Additional contributions [file jamaneurol-e242843-s001.pdf]

# Supplemental Online Content

Belloy ME, Le Guen Y, Stewart I, et al. Role of the X chromosome in Alzheimer disease genetics. *JAMA Neurology*. Published online September 3, 2024.
doi:10.1001/jamaneurol.2024.2843

**eMethods**

ADGC & ADSP Phenotype Ascertainment

ADGC & ADSP Genetic Data Quality Control and Processing

ADGC & ADSP Statistical Analyses

UKB Phenotype Ascertainment

UKB Genetic Data Quality Control and Processing

UKB Statistical Analyses

FinnGen

MVP Phenotype Ascertainment

MVP Genetic Data Quality Control and Processing

MVP Statistical Analyses

General Statistical Analyses

**eDiscussion**

**eFigure 1.** Admixture plot across the five major super populations, for European ancestry case-control participants included in ADGC and ADSP

**eFigure 2.** UKB beta coefficient adjustment onto a regular case-control scale

**eFigure 3.** QQplot for the non-stratified AD XWAS meta-analysis including all data

**eFigure 4.** Forest plots for all 6 lead variants from the non-stratified AD XWAS

**eFigure 5.** Forest plots for all 6 lead variants from the non-stratified AD XWAS and locus zoom plots for all 6 lead variants from the non-stratified AD XWAS

© 2024 Belloy ME et al. *JAMA Neurology.*

**eFigure 6.** *CHST7* Colocalization plots

**eFigure 7.** *SLC9A7* colocalization plots and *SLC9A7* colocalization plots

**eFigure 8.** *SLC9A7* colocalization plots

**eFigure 9.** *MAP7D3* colocalization plots and *MAP7D3* colocalization plots

**eFigure 10.** *MTMR1* colocalization plots

**eFigure 11.** Evaluation of escape from XCI in sex-stratified AD XWAS

**eTable 1.** Overview of genotyping platforms across all available AD-related genetic data

**eTable 2.** Overview of ADSP available through NIAGADS DSS (NG00067)

**eTable 3.** Overview of participant demographics

**eTable 4.** Overview of variant counts in ADGC cohorts with SNP arrays

**eTable 5.** Overview of variant counts across datasets after quality control and intersection with ADGC

**eTable 6.** Phenotype scoring and rescaling approach for the UKB non-stratified AD XWAS for a random XCI model

**eTable 7.** Phenotype scoring and rescaling approach for the UKB sex-stratified AD XWAS

**eTable 8.** Genetic colocalization with quantitative trait locus data: Extension of Table 2 without collapsing results from overlapping tissues

**eTable 9.** Frequency in cases and controls for SLC9A7 lead variant across cohorts

**eTable 10.** Evaluation of sex-specific effects and escape from X chromosome inactivation (XCI) for lead variants from non-stratified AD XWAS

**eTable 11.** Effect sizes for *SLC9A7* lead variant on *SLC9A7* expression in brain tissue

**eReferences**

**eAppendix.** Additional contributions

This supplemental material has been provided by the authors to give readers additional information about their work.

© 2024 Belloy ME et al. *JAMA Neurology.*

## eMethods

In the current study, we used data from a variety of cohorts and sequencing projects related to AD[1–23]. All available genetic/phenotypic data were jointly harmonized with the purpose of performing phenotype/covariate harmonization. Details are provided below.

### ADGC & ADSP Phenotype Ascertainment

*Cohorts and Phenotype Ascertainment*

Details on phenotype ascertainment are described elsewhere[1–5,7]. Briefly, all individuals with a diagnosis of AD met National Institute of Neurological and Communicative Disorders and Stroke/Alzheimer's Disease and Related Disorders Association (NINCDS-ADRDA) criteria for definite, probable, or possible late-onset AD[6], or met Diagnosis and Statistical Manual of Mental Disorders IV-V (DSMIV-V) criteria[8–10], or had a clinical dementia rating (CDR® Dementia Staging Instrument[11]) > 0.5. Some cohorts verified AD diagnoses through neuropathology, using Braak staging[12], CERAD scoring[22], or National Institute on Aging Reagan (NIA-Reagan) 1997 criteria[13]. Cognitively normal subjects did not have AD according to the above clinical AD criteria, did not have a diagnosis of mild-cognitive impairment (MCI), and had a CDR of 0 and/or Mini-Mental State Examination (MMSE[14]) > 25. In MIRAGE, control status was evaluated through a Modified Telephone Interview of Cognitive Status score ≥ 86 (a telephone version of the MMSE)[15].

Further, the National Alzheimer's Coordinating Center (NACC), Rush University Religious Orders Study/Memory and Aging Project (ROSMAP), and Alzheimer's Disease Neuroimaging Initiative (ADNI), are longitudinal cohorts that provide detailed information regarding clinical status (control, MCI, demented) and presumed disease etiology at repeated examinations. Additionally, deceased subjects are assessed for neuropathology. Where possible, in NACC, a final diagnosis of MCI or possible/probable/definite AD was obtained using NIA Alzheimer's Association (NIA-AA) 2011 criteria[16,17]. In all three cohorts, AD diagnoses were verified by neuropathology as middle or high AD likelihood following NIA-Reagan 1997 criteria (moderate to frequent neuritic plaques and Braak stage III-VI)[13]. In concordance with the category "possible AD dementia with evidence of the AD pathophysiological process" from the NIA-AA 2011 criteria[16], we attributed possible AD diagnoses to subjects who met clinical criteria for non-AD dementia but also met AD neuropathological criteria. In concordance with the NIA-AA 2011/2012 framework[17,18], we also evaluated neuropathology in MCI subjects to verify presumed AD etiology. Controls were not re-evaluated based on neuropathology data. Subjects that reverted from dementia to control status during longitudinal follow-up were excluded. Additional cohort-specific details are listed below.

*NACC*

Genotyping waves 1 through 7 from the Alzheimer's Disease Centers (ADC1-7) and a subset of the ADSP projects include subjects ascertained and evaluated by the clinical and neuropathological cores of 32 NIA-funded ADCs. NACC coordinates the collection of these phenotypes, implements diagnoses (cognitively normal, cognitively impaired but not MCI, MCI, demented; and presumed disease etiology), and then provides all data to researchers under the form of the Minimum Data Set (MDS), Uniform Data Set (UDS)[19,20,23], and Neuropathology data set (NP)[21]. The MDS represents an older subset of the NACC data and only contains cross-sectional data, while the more recent UDS provides longitudinal phenotypes and covariates. Since 2015, the UDS was updated to incorporate the NIA-AA 2011 criteria for MCI and AD[17,24]. In the current study, we used the UDS and NP for which data was collected between September 2005 and March 2022, to determine phenotypes for subjects in ADC1-7, ADSP WES/WGS, and ADGC Exome arrays.

Subjects that had a diagnosis of Down syndrome, central nervous system neoplasm, bipolar disorder, schizophrenia, alcohol-induced dementia, or substance-abuse-induced dementia, were excluded. Subjects carrying mutations of dominantly inherited AD or frontotemporal lobar degeneration (FTLD) were also excluded. Subjects with a final diagnosis of MCI or dementia, for which the etiology was unknown, not due to AD, or only secondary due to AD (and without AD neuropathological information), were excluded. Subjects with a final diagnosis of "cognitively impaired but not MCI", but having no other neurological disorder, were kept as controls, considering that this more consistently matched control criteria in many of the other cohorts considered in this study.

*ROSMAP*

In ROSMAP, subjects were diagnosed at each visit: as possible/probable AD according to NINCDS-ADRDA criteria[6]; as MCI when judged to have cognitive impairment but not meeting dementia criteria according to the clinician; or as control when there was no cognitive impairment or the subject did not meet dementia criteria[25,26]. At time of death, a final clinical diagnosis was made by an expert neurologist, followed by a case conference consensus review (blinded to postmortem data)[27].

*ADNI*

In ADNI, subjects were diagnosed at regular visits: as possible/probable AD according to NINCDS-ADRDA criteria[6]; as MCI according to Petersen/Winblad criteria; or as control when not demented, not MCI, CDR = 0, and MMSE > 28. Neuropathology assessments followed the NACC NP framework.

© 2024 Belloy ME et al. *JAMA Neurology.*

*Phenotype Harmonization*

The available sample contained many subjects that were genotyped multiple times across different studies. This largely reflected efforts from the ADGC, ADSP, and AMP-AD, to perform next-generation sequencing (NGS) on existing cohort samples for the purpose of rare variant discovery and AD gene prioritization. In other instances, participants were recruited in different studies at different times. Therefore, to handle potential duplicate discordance and phenotype heterogeneity, we implemented a cross-sample phenotype harmonization procedure aiming to standardize pathology-verified diagnoses where possible, share unique missing information across all duplicate entries of a given subject, resolve longitudinal changes in diagnosis, and flag subjects with unresolvable duplicate discordance for exclusion.

Duplicate samples were identified by determining genetic cryptic relatedness (cf. below), but for sample cross-referencing did not include known identical twins in LOAD and ROSMAP samples. First, duplicate samples were flagged as discordant if their age-at-death information differed by more than 2 years or if pathology measures (Braak or neuritic plaque density) differed. Across all cohorts, where possible, AD diagnoses were verified by neuropathology as middle or high AD likelihood following NIA-Reagan 1997 criteria (moderate to frequent neuritic plaques and Braak stage III-VI)[13]. Additionally, when only either neuritic plaque or Braak information was available and in line with NIA-Reagan 1997 middle or high AD likelihood criteria, and/or the cohort/project demographics provided a diagnosis of definite AD, the subject was considered to have pathology-verified AD status. Cognitively normal (CN) subjects with evidence of AD pathology were kept as CN. Further, if at least one entry across duplicate samples indicated a diagnosis of Down syndrome, central nervous system neoplasm, bipolar disorder, schizophrenia, alcohol-induced dementia, substance-abuse-induced dementia, neurological (not including Parkinson's disease), or systemic disease despite being cognitively normal, or carrying mutations of dominantly inherited AD or frontotemporal lobar degeneration (FTLD), then all duplicate samples were marked as such and flagged for exclusion. Extending on the above, all genetic samples were checked for the presence of known pathogenic mutations on *APP, PSEN1, PSEN2,* and *MAPT*, whereby carriers and their duplicate samples were flagged for exclusion.

Then, duplicate samples with differing age entries (i.e. longitudinal changes) were evaluated. Reversions from AD or dementia to MCI status, or from MCI to cognitively normal (CN) status, were permitted, but reversions from AD or non-AD dementia to CN status were flagged for exclusion. "Reversions" from AD to non-AD dementia status were permitted, unless pathology (cf. above) indicated the presence of AD pathology, thereby marking the subject as AD. Vice versa, "conversions" from non-AD

dementia to AD status were permitted, unless pathology (cf. above) indicated no presence of AD pathology, thereby marking the subject as non-AD dementia. All other types of conversions were directly permitted. Then, duplicate samples for which the diagnoses at the oldest shared age entries differed, or for which diagnoses differed but age was consistent (i.e. apparent cross-sectional discordances), were evaluated. Discordances between AD and non-AD dementia status were resolved based on pathology (cf. above) or flagged as discordant if no pathology data was available. Discordances between CN and AD status, or CN and non-AD dementia status, were resolved as respectively AD or non-AD dementia when those dementia diagnoses corresponded to a unique age-at-onset (of symptoms) without other available age information (i.e. indicating that a conversion likely occurred after the subject was lost to follow-up in the cohort that last observed a CN status), or, were flagged as discordant if duplicate entries shared the same age-at-examination and age-at-last-exam. Discordances between CN and MCI status, or MCI and AD status, or MCI and non-AD dementia status, were resolved as respectively MCI, AD, or non-AD dementia (i.e. keeping the most severe diagnosis).

Finally, once all clinical diagnostic and pathological data were unified across duplicate entries, pathological criteria were applied once more to obtain the final diagnoses. Where possible, AD diagnoses were verified by neuropathology as middle or high AD likelihood following NIA-Reagan 1997 criteria (moderate to frequent neuritic plaques and Braak stage III-VI)[13]. In concordance with the category "possible AD dementia with evidence of the AD pathophysiological process" from the NIA-AA 2011 criteria[16], we attributed possible AD diagnoses to subjects who met clinical criteria for non-AD dementia but also met AD neuropathological criteria. In concordance with the NIA-AA 2011/2012 framework[17,18], we also evaluated neuropathology in MCI subjects to verify presumed AD etiology and considered subjects as cases if AD pathology, following NIA-Reagan 1997 criteria (cf. above), was present (i.e. marking high likelihood of AD etiology). Controls were not re-evaluated based on neuropathology data.

Beyond cross-referencing clinical diagnostic and pathological data across subjects, other covariates were considered for cross-referencing or sharing in case of missingness across duplicate entries. These included age-at-onset of cognitive symptoms, age-at-examination providing clinical diagnosis, at-at-last exam, age-at-death, sex, race, ethnicity, *APOE* genotype provided from demographics, *APOE* genotype provided from whole-genome sequencing, and *APOE* genotype provided from whole-exome sequencing. Duplicate entries with discordant sex or race information were flagged for exclusion.

© 2024 Belloy ME et al. *JAMA Neurology.*

**ADGC and ADSP Genetic Data Quality Control and Processing**

*Ascertainment of Genetic Data*

Genotypes were available from high-density single-nucleotide polymorphism (SNP) genotyping microarrays (Illumina or Affymetrix) for ADGC or whole genome sequencing (WGS) for ADSP (**eTable 1-2**). Genotype samples had their genetic variants lifted to hg38 using liftOver if not released in hg38 and annotated using dbSNP153 variant identifiers[28].

*ADGC Autosomal Quality Control*

Autosomal variants were extracted from the SNP array data and further processed in several stages. In each cohort/platform/array, variants were excluded based on genotyping rate (<95%), MAF<1%, and Hardy-Weinberg equilibrium in controls ($p<10^{-6}$) using PLINK v1.9[29]. As in our prior work[30], information derived from the gnomAD v.3.1 database[31] was used to filter out SNPs that met one of the following exclusion criteria: (i) located in a low complexity region, (ii) located within common structural variants (MAF > 1%), (iii) multiallelic SNPs with MAF > 1% for at least two alternate alleles, (iv) located within a common insertion/deletion, (v) having any flag different than PASS in gnomAD, (vi) having potential probe polymorphisms.

*ADSP Autosomal Quality Control*

The ADSP WGS data (NG00067.v5) were joint called by the ADSP following the SNP/Indel Variant Calling Pipeline and data management tool used for the analysis of genome and exome sequencing for the Alzheimer's Disease Sequencing Project (VCPA)[32]. The current analyses of ADSP WGS were restricted to bi-allelic variants, to which we applied the Variant Quality Score Recalibration (VSQR) quality control filter ("PASS" variants; GATK v4.1)[33]. Variants with a genotyping rate less than 80%, deviating from Hardy Weinberg Equilibrium (HWE) in the full sample or in controls ($p<10^{-6}$), and a minor allele count less than 10, were excluded. Consistent with the methodology detailed in Belloy et al. 2022[34], we then applied several filters to remove artifactual variants: (i) variants that represented sequencing center or platform artifacts as identified by Fisher exact testing in controls ($p<10^{-5}$), (ii) variants reported in gnomAD v3.1[31] to have a "non-PASS", falling in a low complexity region, or showing more than 10% allele frequency devation between our European ancestry control participants in ADSP and non-Finnish European participants in gnomAD, and (iii) duplicate discordance variants that show discrepancies across several 100 technical duplicates present in ADSP.

© 2024 Belloy ME et al. *JAMA Neurology.*

*Genetic Relationship Determination using King*

Across all cohorts, the relatedness of subjects (after QC indicated above) was evaluated through identity-by-descent (IBD) analysis (using directly genotyped non-palindromic SNPs shared across all genetic datasets with a call rate > 95% & minor allele frequency (MAF)>1%)[35]. This outcome was used for duplicate tracking across samples, which in turn was used to enable phenotype harmonization (cf. above).

*Ancestry Determination*

Individual ancestries were determined using SNPweights v.2.1 with populations from the 1000 Genomes Consortium as a reference[36,37]. By applying an ancestry percentage cut-off ≥ 75%, the samples were stratified into the five super populations, South-Asians (SAS), East-Asians (EAS), Amerindians (AMR), Africans (AFR) and Europeans (EUR) (**eFigure 1**). When multiple samples were available for a single unique individual, the ancestry was inferred from the sample with the highest genetic coverage.

*Restriction to European ancestry for XWAS*

XWAS were focused on the European ancestry subsample. Two main reasons explain the more extreme population structure on the X-chromosome compared to autosomes: (i) the X-chromosome has a smaller effective population size and thus the rate of genetic drift of X-linked loci is amplified, (ii) local adaptation will lead to higher levels of differentiation between geographically isolated populations[38]. As such, to better control for population structure, we restricted our analyses to European ancestry participants.

*Relationship Determination and Principal Component Analysis using GENESIS*

For ADGC and ADSP data respectively, the relatedness of subjects and principal components capturing population substructure were determined using IBD and principal component analyses (PCA) as implemented through the R package GENESIS (R v3.6.0)[39]. Specifically, this approach first uses an R-implementation of KING-robust to determine kinship coefficients that take into account ancestry divergence. The derived pairwise kinship coefficients are then used to perform a PCA in related samples (PC-AiR) providing accurate ancestry inference not confounded by family structure. The latter output is then used to estimate kinship coefficients using PC-Relate, which accounts for population structure (ancestry) among sample individuals through the use of ancestry representative principal components (PCs) to provide accurate relatedness estimates due only to recent family (pedigree) structure. For each respective data set, these analyses were performed on pruned SNPs ($R^2 < 0.5$, call rate > 95%, MAF > 1%, and excluding palindromic SNPs) in non-Hispanic White European ancestry individuals.

*ADGC X chromosome Quality Control and TOPMed Imputation*

The X chromosome variants underwent a similar harmonization pipeline as the autosomes. We excluded multi-allelic SNPs, SNPs within structural variations, and potential probe polymorphism SNPs. Additionally, our analysis excluded the pseudoautosomal regions of the X chromosome and used only the European ancestry participants as derived above. Several steps were performed to avoid spurious findings: (i) variants with less than 95% genotyping rate and (ii) individuals with more than 5% genotype missingness were excluded. (iii) Reported sex was checked using PLINK1.9 --*check-sex* flag[29], with 0.4 max value for females and 0.94 min value for men, and all individuals with a discordant sex label were excluded. (iv) Heterozygous SNPs in males were set as missing in males, while (v) SNPs with differential missingness between AD cases and controls were removed ($p<10^{-5}$ per cohort/platform/array). (vi) HWE was tested in female controls and SNPs with $p<10^{-5}$ were removed (per cohort/platform/array). (vii) Any monomorphic SNPs that remained were removed. (viii) Differential missingness and differential MAF between males and females were both tested and SNPs with $p<10^{-5}$, for either one of the tests, were excluded (per cohort/platform/array). Finally, as for the autosomes and based on gnomAD v3.1[31] information, we filtered variants (ix) located in a low complexity region, (x) located within common structural variants (MAF > 1%), (xi) multiallelic SNPs with MAF>1% for at least two alternate alleles, (xii) located within a common insertion/deletion, (xiii) having any flag different than PASS in gnomAD v.3.1, (xiv) having potential probe polymorphisms (xv) more than 10% MAF difference with gnomAD frequency in non-Finnish Eurpeans. The remaining SNPs were checked for consistency with the TOPMed panel, flipping of palindromic SNPs, and were imputed on the TOPMed Imputation server[40,41], which uses Minimac 4 for imputation. The following parameters were selected: reference panel TOPMed-r2 (2022), phasing with Eagle v2.4, r-square imputation score cut off 0.3.

*ADSP X chromosome Quality Control*

The X chromosome variants underwent a similar harmonization pipeline as the autosomes. Our analysis excluded the pseudoautosomal regions of the X chromosome and used only the European ancestry participants. The ADSP WGS data for X chromosome (NG00067.v5) were joint called by the ADSP following the SNP/Indel Variant Calling Pipeline and data management tool used for the analysis of genome and exome sequencing for the Alzheimer's Disease Sequencing Project (VCPA)[32]. The current analyses of ADSP WGS were restricted to bi-allelic variants, to which we applied the Variant Quality Score Recalibration (VSQR) quality control filter ("PASS" variants; GATK v4.1)[33]. Variants with a genotyping rate of less than 80% and a minor allele count of less than 2 were excluded. Consistent with the methodology detailed in

Belloy et al. 2022[34], we then applied several filters to remove artifactual variants: (i) variants that represent sequencing center or platform artifacts as identified by Fisher exact testing in controls ($p<10^{-5}$), (ii) variants reported in gnomAD v3.1 to have a “non-PASS”, falling in a low complexity region, or showing more than 10% allele frequency devation between our European ancestry control participants in ADSP and non-Finnish European participants in gnomAD, and (iii) duplicate discordance variants that show discrepancies across several 100 technical duplicates present in ADSP.

Several additional steps were performed to avoid spurious findings: (i) Heterozygous SNPs in males were set as missing in males, (ii) variants with a genotyping rate less than 80% in controls, cases, men, or women were excluded, (iii) variants with differential missingness between AD cases and controls were removed ($p<10^{-10}$ for the full sample). (iv) HWE was tested in female and male controls using the Plink --hardy command that allows joint sex evaluation and variants with $p<10^{-5}$ were removed (for the full sample). (v) Differential missingness between males and females was tested and SNPs with $p<10^{-20}$ were excluded (for the full sample).

© 2024 Belloy ME et al. *JAMA Neurology.*

**ADGC & ADSP Statistical Analyses**

*Case-control XWAS*

All association analyses with AD risk were adjusted for sex (in non-stratified XWAS), array type, the first 5 genetic prinicipal components (PC-AiRs), *APOE**4 dosage (0/1/2), and *APOE**2 dosage (0/1/2). Age adjustment in case-control analyses was not performed, given that the current AD genetic samples often showed younger ages for cases than controls due to the use of age-at-onset information (**eTable3**), which violates the assumption for age adjustment (which is that older age is associated with increased AD incidence). In prior work, we showed that age adjustment in such scenarios leads to significantly decreased power for genetic association analyses[30]. Adjustment for *APOE* genotypes is relevant given the established interactions with sex[42], which may notably be relevant to the X chromosome and could lead to increased model noise if not accounted for. Additionally, the case-control clinical cohorts are enriched for *APOE**4 cases compared to population-based studies[42], which may further exacerbate any potential confounding effects.

Cohorts from ADGC were pooled into a mega-analysis. LMM-BOLT was used in both ADGC and ADSP[43], using autosomal data to derive genetic relationship matrices to allow the inclusion of related subjects. Resultant betas were converted to traditional odds ratios using the transformation approach as detailed in the LMM-BOLT manual. Across ADGC and ADSP, subjects were unrelated down to 1st degree.

*Age information*

For cases that only had age-at-death (AAD) available, the final ages used for regression analysis were subtracted by 10 years to approximate age-at-onset (AAO). This reflects expected mean delays between AAO and AAD for AD patients[44], and is consistent with the derived age covariate for AD cohorts provided by the Alzheimer's Disease Genetics Consortium (ADGC) on NIAGADS[45]. In cohorts that provide conversion information but not AAO, age-at-examination (AAE) was used and followed a prioritization of age-at-MCI-diagnosis > age-at-dementia-diagnosis (incident) > age-at-dementia diagnosis (prevalent). This was done to most closely approximate AAO. For the remaining control samples, age-at-last-examination (AAL) was used. After implementing these criteria, samples were filtered to have a minimal age of 60 years. Some samples were censored at ages 90+, for which we assumed the age was 90 (since there was no way to estimate the actual age).

© 2024 Belloy ME et al. *JAMA Neurology.*

**UKB Phenotype ascertainment**

Detailed descriptions of all the variables and field provided by UKB are provided elsewhere[46].

In the first round of phenotype ascertainment, we derived health-registry-confirmed AD status and related age information for the individuals directly. Subjects were assumed to be controls if they had no other diagnosis inferred from health registry information relevant to dementia status. We specifically considered the following data fields and entries: *Diagnoses_main_ICD10* [G300,G301,G308,G309,F000,F001,F002,F009], *Diagnoses_secondary_ICD10* [G300,G301,G308, G309,F000,F001,F002,F009], *Date_of_first_in_patient_diagnosis_main_ICD10* [if date provided], *Date_of_first_in_patient_diagnosis_ICD10* [if date provided], *Source_of_alzheimers_disease_ report* [0,1,11,12,2,21,22 = selft report, hospital admission, death record], *Date_of_alzheimers_disease_ report* [if date provided], *Source_of_all_cause_dementia_report* [0,1,11,12,2,21,22 = selft report, hospital admission, death record], *Source_of_frontotemporal_dementia_report* [any entry], *Source_of_vascular_ dementia_report* [any entry], and *Date_of_all_cause_dementia_report* [if data provided]. The above fields were used to determine dementia status, allowing us to differentiate between late-onset AD individuals (LOAD), early-onset AD (EOAD), vascular dementia, frontotemporal dementia, and other all-cause dementia participants. For the health-registry AD phenotype, cases were restricted to all LOAD individuals. The above fields were further used to determine the earliest available age at which a dementia occurrence or report was made. Age information for controls was available from the variables: *Age_when_attended_assessment_centre* [oldest age entry retrieved] and *Age_at_death*.

We then identified the proxy ADD case and control status and related age by accessing the following fields and entries: *Illnesses_of_father*, *Illnesses_of_mother*, *Illnesses_of_sibblings*, *Fathers_age*, *Fathers_age_at_death*, *Mothers_age*, and *Mothers_age_at_death* (where it should be noted that age and sex info was not available for siblings). The youngest reported age was used for proxy ADD cases, while the oldest reported age was used for proxy controls. Proxy status was ignored if subjects were adopted.

Both health-registry-confirmed AD status and proxy ADD status were then combined into a single phenotype (cf. **eTables6-7**). Notably, all subjects were ages >60y in at least the subject or one parent.

**UKB Genetic Data Quality Control and Processing**

A Detailed description of all the UKB genetic data and processing is provided elsewhere[46]. Specifically, we accessed SNP array data imputed to the Haplotype Reference Consortium (HRC) and UK10K haplotype

resource. We further filtered to subjects with consent, passing sex check QC, no heterozygosity outliers, having age information available, and belonging to a white ethnic background (field *Ethnic_background* [1001,1002,1003]). We then identified a homogenous ancestry cluster within this group using "aberrant" on the first 20 genetic PCs, as in Schwartzentruber et al. 2021[47].

**UKB Statistical Analyses**

All association analyses with the AD phenotype were adjusted for sex (in non-stratified XWAS), array type, assessment center, the first 20 genetic principal components provided by UKB, and *APOE**4/2 dosage. LMM-BOLT was used (as was done for ADGC and ADSP)[43], using autosomal data to derive genetic relationship matrices to allow the inclusion of related subjects. Resultant betas were converted to traditional odds ratios using the transformation approach as detailed in the LMM-BOLT manual. Additionally, since the UKB XWAS leveraged the proxy phenotype, an additional correction factor was needed to rescale beta coefficients onto a regular case-control scale. This correction is detailed in **eTables6-7**.

© 2024 Belloy ME et al. *JAMA Neurology.*

**FinnGen**

The FinnGen study is a large-scale genomics initiative that has analyzed over 500,000 Finnish biobank samples and correlated genetic variation with health data to understand disease mechanisms and predispositions. The project is a collaboration between research organisations and biobanks within Finland and international industry partners.

Ascertainment of FinnGen phenotype and genotype data is described in detail elsewhere[48]. Summary statistics were available from version 10 (v10) of the publicly released set of genetic summary statistics made available by FinnGen here: https://www.finngen.fi/en/access_results. Documentation on Genetic data processing and statistical analyses is provided here: https://finngen.gitbook.io/documentation. FinnGen made us of Regenie to include related individuals in the genetic association analyses[49]. Information about phenotypes and endpoints is provided here: https://www.finngen.fi/en/researchers/clinical-endpoints, https://r10.risteys.finngen.fi/. We specifically leveraged the "Alzheimer's disease, wide definition" phenotype.

© 2024 Belloy ME et al. *JAMA Neurology.*

**MVP Phenotype Ascertainment**

Generation of phenotypes for MVP mirrors methods as described in Sherva et al. 2023[50], but was updated based on the more recent MVP 2022_1 data release. Briefly, MVP phenotype data were generated from VA electronic medical records. Alzheimer's disease and related dementia (ADRD) cases were identified on the basis of International Classification of Disease (ICD) 9 and 10 codes. Information on proxy cases (parental dementia) was obtained from the MVP Baseline Survey[51].

**MVP Genetic Data Quality Control and Processing**

Generation and quality control of the MVP genetic data is described in detail elsewhere[52]. Briefly, the genetic data were genotyped using the MVP 1.0 custom Axiom array13, phasing was performed by SHAPEIT4 v 4.1.3, and imputation was performed with MINIMAC4 based on the TopMed imputation reference panel[40]. Variants were then filtered to imputation scores > 0.4 and allele frequencies >= 0.1% in the full dataset. The subset of European-ancestry MVP participants, as determined by the genetically informed Harmonized Ancestry and Race/Ethnicity (HARE) method[53], was then extracted for XWAS and variants were subsequently filtered to allele frequencies >= 0.05%.

**MVP Statistical Analyses**

As in Sherva et al. 2023, our ADRD case-control analysis was independent of the cohort used for the proxy analysis. The MVP case-control XWAS (MVP-1) included ICD-identified cases with onset after age 60. The controls were all over age 65 without any dementia or mild cognitive impairment ICD codes and without a history of AD medication usage. The MVP proxy XWAS (MVP-2) was performed separately using a set of controls without any report of parental dementia. Only survey data for Veteran participants over age 45 at last visit were included in proxy analyses (age for parents was not available). The proxy analyses were focused on maternal phenotypes only. This represents the majority of proxy samples since paternal phenotypes cannot be used for men and there was a relative paucity of women. In both the MVP-1 and MVP-2 cohorts, Plink was used to conduct case-control logistic regression analyses on unrelated subjects. For the proxy XWAS, a correction factor was needed to rescale beta coefficients onto a regular case-control scale. This correction is detailed in **eTables6-7**.

© 2024 Belloy ME et al. *JAMA Neurology.*

**General Statistical Analyses**

*Meta-analysis.*

AD XWAS meta-analyses were conducted using genome-wide, fixed effects inverse-variance weighted meta-analysis as implemented in GWAMA[54].

*Sex heterogeneity.*

Sex heterogeneity tests were evaluated as: Z-value = $(Beta_{men} - Beta_{women})/\sqrt{(SE_{men}^2 + SE_{women}^2)}$. P-values were then determined using the normal distribution with a two-sided hypothesis in R using the following formulation: P-value = 2*pnorm(q=Z-value, lower.tail=FALSE).

*Escape from X chromosome inactivation.*

XCI escape status with regard to AD was evaluated by dividing XWAS beta coefficients from men by beta coefficients from women (similar as in Sidorenko et al. 2019[55]), where a ratio close to 2 suggests no escape from XCI (male beta coefficients for a single active X genotype are double compared to those in women where the X genotype undergoes random XCI) and a ratio close to 1 suggests escape from XCI (the beta coefficients in women become consistent with those in men if there is escape from XCI). We further identified if there was any prior support for XCI at each identified locus by consulting 2 published research articles, containing summaries of genes with prior reported XCI status in addition to novel findings[56,57].

*Genetic Colocalization.*

QTL resources with X chromosome genetic data were available for various tissues from GTEx[58], brain tissue from Wingo et al. 2023[59], and brain tissue (CommonMind, Braineac2), monocytes (CEDAR, Fairfax et al. 2014), microglia (Young et al. 2019), and T cells (Kasela et al. 2017), uniformly processed by the eQTL Catalogue[60]. Colocalization was considered for all genes in each associated locus using a 2Mb window centered on the lead XWAS variant. Evidence for colocalization was considered at colocalization posterior probability (PP4)>0.7 (as in Bellenguez et al. 2022)[61]. Additionally, colocalizations with PP4>0.7 were annotated to indicate whether the QTL passed significane criteria in the respective data/tissues (FDR correction in GTEx and Wingo et al. 2023; P<1e-5 in the eQTL Catalogue which corresponds on average to FDR corrected QTL P-values (no FDR corrected P-values were provided)). Genetic colocalization analyses were restricted to variants seen in 95% of the full XWAS meta-analysis and for which MAF did not deviate >10% across the XWAS and QTL data. We used the “coloc.abf” function from the *coloc* package (R-v.4.2.1)[62], providing sample size, P-value, and MAF. We did not use beta coefficients and standard errors due to potential concerns for variants with MAF close to 50%.

© 2024 Belloy ME et al. *JAMA Neurology.*

**Supplemental Discussion: *SLC9A7*.**

The need to maintain the pH of the cytoplasm and intracellular compartments within close parameters is essential to the functioning of the cell and the regulation of intracellular transport mechanisms. Many if not most of the biological functions of the cell have evolved to take place at or near a narrowly defined pH optimum and any deviation from this optimum results in progressively impaired functions and ultimately death of the cell and the organism. Enzymes have evolved to function at a pH optimum that resides usually around 7.4 in the cytoplasm. However, enzymes like lysosomal proteases or lipid metabolic enzymes have evolved to be most active at an acidic pH that is closer to 5. Indeed, activation of these enzymes often occurs only once they have progressed into heavily acidified compartments in order to prevent their premature activation in the biosynthetic or secretory pathway where they could prematurely degrade newly synthesized molecules. The acidification of these intracellular organelles is mediated by the vacuolar ATPase (vATPase) a.k.a. the proton pump. This is an energy intensive process by which the vATPase establishes an electrochemical proton gradient across diverse cellular membranes which not only regulates the activity and rate of catalytic events but also drives the appropriate sorting of intracellular cargo-containing compartments (reviewed in (Vasanthakumar and Rubinstein, 2020). For instance, alkalinization of intracellular compartments of the Golgie and the endolysosomal pathway with weak bases like ammonium chloride or chloroquine prevents or delays the processing of endocytosed cargo and the timely recycling of endocytic receptors from early endosomes (Thorens and Vassalli, 1986). Therefore, the kinetics at which acidification of specific compartments occurs is of the utmost importance for the coordinated and regulated functioning of the cell.

The electrochemical gradient that is generated by the vATPase also drives other ion exchange mechanisms that are themselves not dependent upon the consumption of ATP. Such mechanisms involve for instance the chloride/proton antiporter, chloride channels and the sodium hydrogen exchangers (NHEs) (Jentsch and Pusch, 2018; Flessner and Orlowski, 2021). The concerted functions of these ion exchangers in conjunction with the vATPase then regulate the ultimate pH and ion composition of the respective compartments in which they reside.

Nine different sodium hydrogen antiporters regulate pH homeostasis in virtually all tissues of the body in this manner (Flessner and Orlowski, 2021). While NHE 1-4 are primarily involved in ion transport at the plasma membrane, NHE 5 to 9 primarily regulate proton, sodium and potassium homeostasis across intracellular membranes. NHE5 and NHE6 control proton exchange in recycling and early endosomes,

while NHE9 has been localized to late endosomes, phagosomes and recycling endosomes. NHE8 appears to be required for perinuclear vesicle fusion and sorting. NHE7, by contrast, localizes to Golgi compartments where it has been implicated in the exchange of protons with the cytoplasm and the acidification of secretory compartments, although its precise functions there require further investigations.

Human genetic defects in *NHE6*, *NHE7* and *NHE9* have been described and found to cause neurodevelopmental syndromes that include autism, epilepsy, mental disability and selective neuronal loss (Flessner and Orlowski, 2021). These findings emphasize the importance of intracellular compartmental pH and ion homeostasis especially for the development and function of the human brain. The critical importance of early endosomal pH regulation is underscored by loss of function mutations in the X-chromosomal *NHE6*, which is the cause for Christianson syndrome. The resulting accelerated and unchecked acidification of the early endosomal compartment results in the premature activation of lysosomal enzymes which in turn mediate the aberrant degradation of various neuronal proteins including neurotrophins and neurotrophin receptors (Ouyang et al., 2013).

At the other end of the spectrum, i.e. in the aging brain, reduced metabolism and impaired energy production would be predicted to adversely impact vATPase function and consequently result in the delayed acidification of the endolysosomal compartment, leading to impaired autophagy and lysosomal degradation of cellular waste products, including amyloid and tau. Such a model of AD pathogenesis is supported by numerous studies in mice and humans that have revealed profound impairments of vATPase-mediated proton translocation activity in mouse models of AD (Lee et al., 2022) as well as the prominent enlargement of endosomes that has been proposed to be the result of impaired endosomal acidification kinetics (Pohlkamp et al., 2021). Consequently, genetic disruption of *NHE6*, the primary proton leak channel in the early endosome, was found to greatly delay amyloid accumulation in an AD mouse model (Pohlkamp et al., 2021). Similarly, pharmacological or genetic inhibition of NHE6 function in cortical neurons completely abolished the endosomal recycling delay and intracellular sequestration of ApoE and excitatory neurotransmitter receptors in the presence of ApoE4 (Xian et al., 2018). Conversely, missense mutations in *NHE7* lead to Golgi alkalinization (Khayat et al., 2019) where NHE7 has been proposed to mediate proton influx from the cytosol in exchange for sodium ions (Milosavljevic et al., 2014). Consistent with the proposed models and the conclusions of these earlier studies, our current finding now further suggests that a genetic polymorphism that results in a modest increase of NHE7 protein expression is neurodevelopmentally neutral, but by disrupting Golgi pH homeostasis also appears

to increase risk for late-onset AD. This finding therefore supports proposed therapeutic interventions where partial pharmacological inhibition of NHEs, i.e. intracellular proton leak channels, during aging to support ailing proton pump activity might stave off or prevent the manifestation of AD (Xian et al., 2018; Pohlkamp et al., 2021).

**References**


Flessner R, Orlowski J (2021) Na+/H+ Exchangers. In: Encyclopedia of Molecular Pharmacology (Offermanns S, Rosenthal W, eds), pp 1047-1062. Cham: Springer International Publishing.

Jentsch TJ, Pusch M (2018) CLC Chloride Channels and Transporters: Structure, Function, Physiology, and Disease. Physiol Rev 98:1493-1590.

Khayat W, Hackett A, Shaw M, Ilie A, Dudding-Byth T, Kalscheuer VM, Christie L, Corbett MA, Juusola J, Friend KL, Kirmse BM, Gecz J, Field M, Orlowski J (2019) A recurrent missense variant in SLC9A7 causes nonsyndromic X-linked intellectual disability with alteration of Golgi acidification and aberrant glycosylation. Hum Mol Genet 28:598-614.

Lee JH, Yang DS, Goulbourne CN, Im E, Stavrides P, Pensalfini A, Chan H, Bouchet-Marquis C, Bleiwas C, Berg MJ, Huo C, Peddy J, Pawlik M, Levy E, Rao M, Staufenbiel M, Nixon RA (2022) Faulty autolysosome acidification in Alzheimer's disease mouse models induces autophagic build-up of Abeta in neurons, yielding senile plaques. Nat Neurosci 25:688-701.

Milosavljevic N, Monet M, Lena I, Brau F, Lacas-Gervais S, Feliciangeli S, Counillon L, Poet M (2014) The intracellular Na(+)/H(+) exchanger NHE7 effects a Na(+)-coupled, but not K(+)-coupled proton-loading mechanism in endocytosis. Cell Rep 7:689-696.

Ouyang Q, Lizarraga SB, Schmidt M, Yang U, Gong J, Ellisor D, Kauer JA, Morrow EM (2013) Christianson syndrome protein NHE6 modulates TrkB endosomal signaling required for neuronal circuit development. Neuron 80:97-112.

Pohlkamp T, Xian X, Wong CH, Durakoglugil MS, Werthmann GC, Saido TC, Evers BM, White CL, 3rd, Connor J, Hammer RE, Herz J (2021) NHE6 depletion corrects ApoE4-mediated synaptic impairments and reduces amyloid plaque load. Elife 10.

Thorens B, Vassalli P (1986) Chloroquine and ammonium chloride prevent terminal glycosylation of immunoglobulins in plasma cells without affecting secretion. Nature 321:618-620.

Vasanthakumar T, Rubinstein JL (2020) Structure and Roles of V-type ATPases. Trends Biochem Sci 45:295-307.

Xian X, Pohlkamp T, Durakoglugil MS, Wong CH, Beck JK, Lane-Donovan C, Plattner F, Herz J (2018) Reversal of ApoE4-induced recycling block as a novel prevention approach for Alzheimer's disease. Elife 7.


© 2024 Belloy ME et al. *JAMA Neurology.*

# eFigures.

© 2024 Belloy ME et al. *JAMA Neurology.*

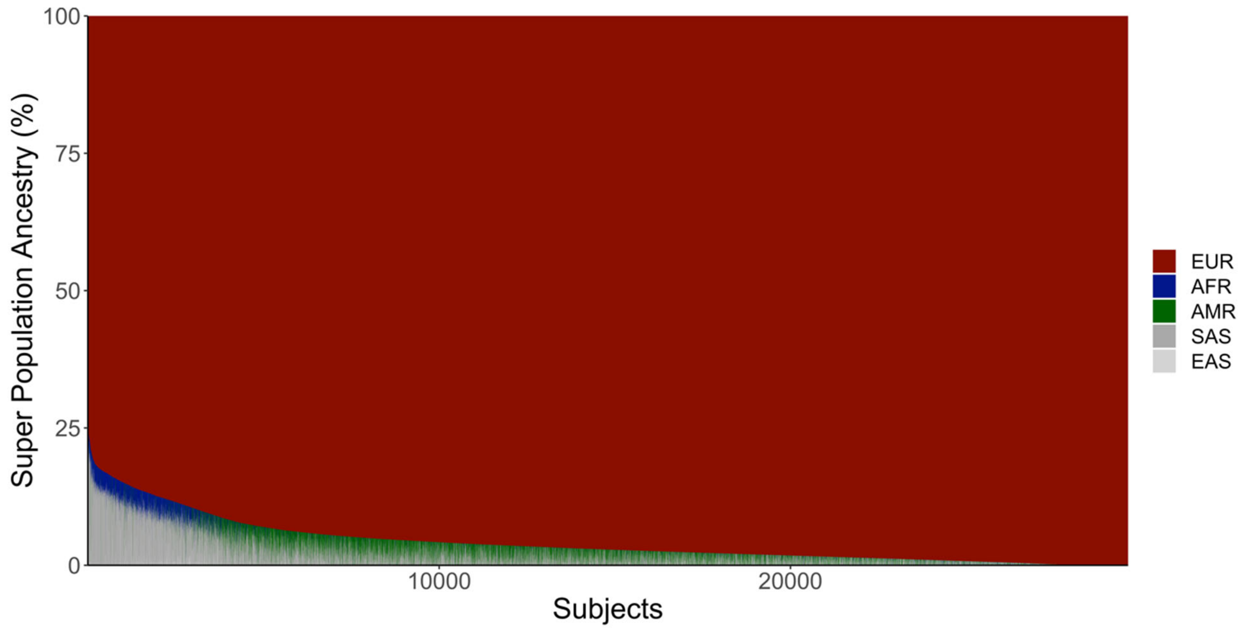


**eFigure 1. Admixture plot across the five major super populations, for European ancestry case-control participants included in ADGC and ADSP.**

*Abbreviations: EUR, European; AFR, African; AMR; Amerindian; SAS, South Asian; EAS; East Asian.*

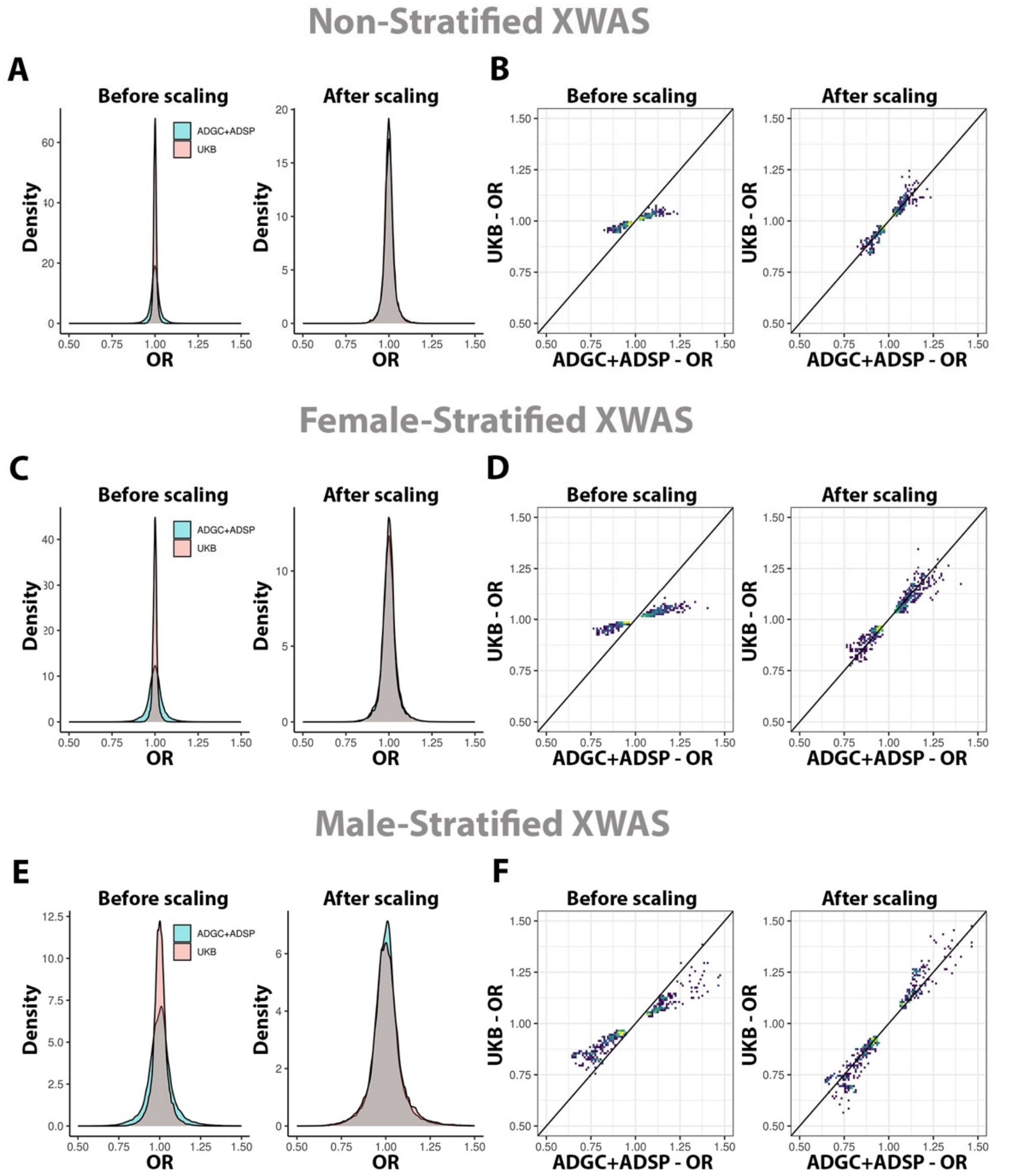


**eFigure 2. UKB beta coefficient adjustment onto a regular case-control scale.** Rescaling for (**A-B**) non-stratified, (**C-D**) female-stratified, and (**E-F**) male-stratified AD XWAS. **A,C,E)** Density plots show beta coefficients for all variants intersecting across ADGC+ADSP and UKB, before and after rescaling. **B,D,F)** Scatter density plots show beta coefficients for prioritized variants intersecting across ADGC+ADSP and UKB, before and after rescaling. Intensity increases from dark blue to bright yellow. A line with slope=1 is plotted for reference. Variants had allele frequencies >= 1%, P<0.1 in both ADGC+ADSP and UKB, concordant effect directions across ADGC+ADSP and UKB, and P<0.01 in ADGC+ADSP+UKB (these variants are more likely to include true associations).

*Abbreviations: OR; Odds Ratio.*

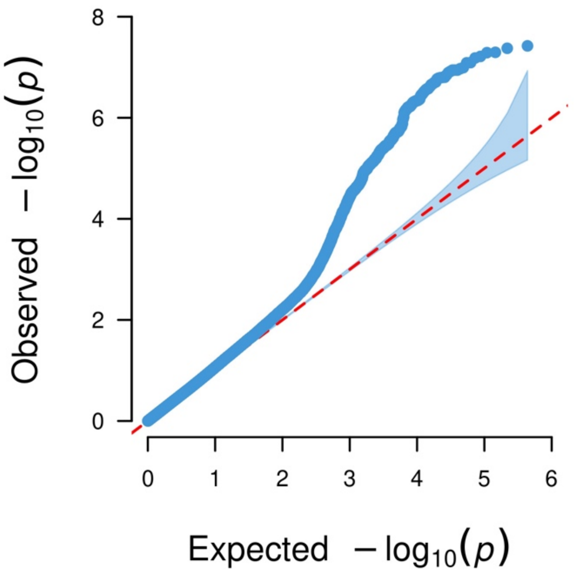


**eFigure 3. QQplot for the non-stratified AD XWAS meta-analysis including all data**. The inflation factor ($\lambda$=1.0671) and sample size-adjusted inflation factor ($\lambda_{1,000}$=1.0003) showed no sign of inflation.

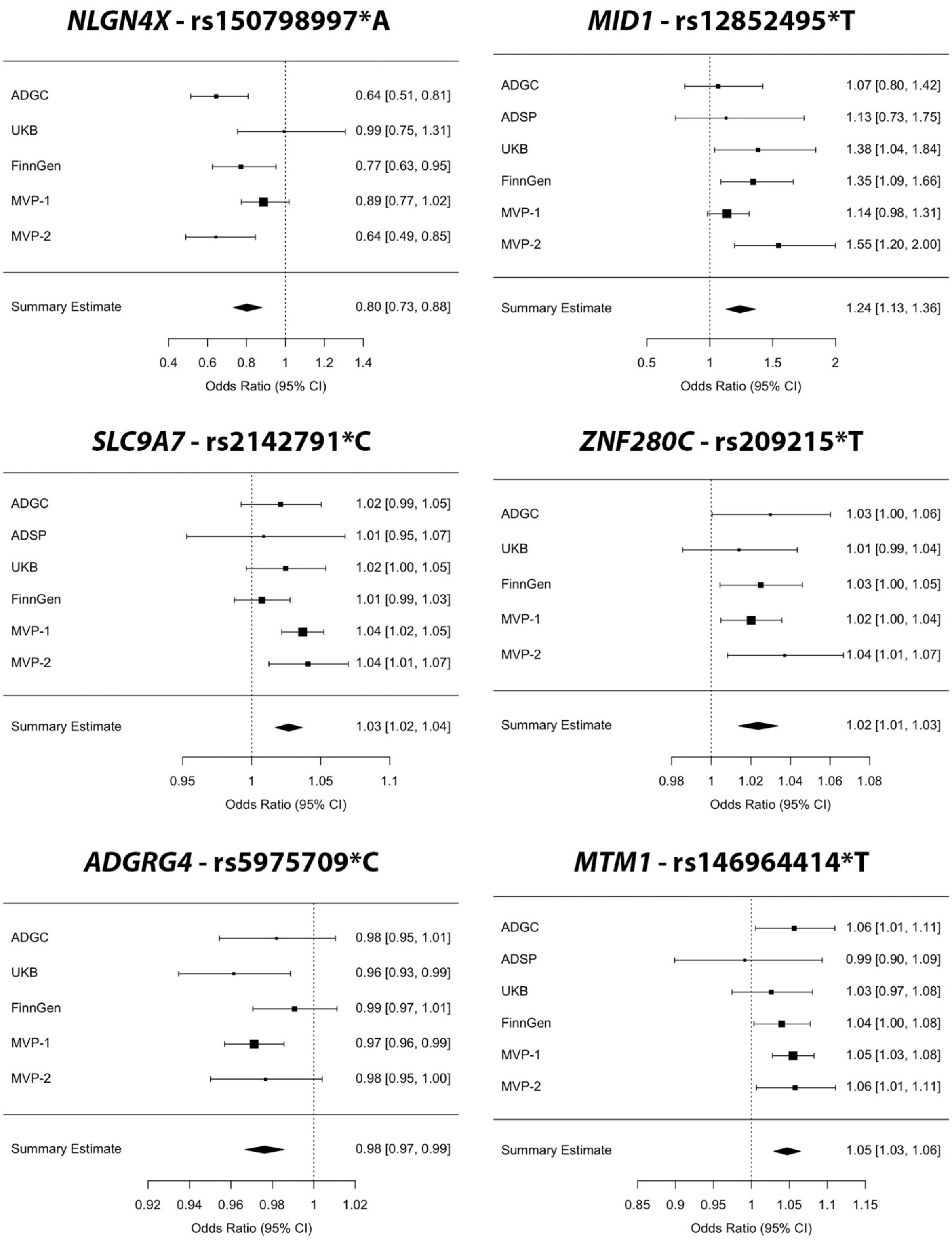


**eFigure 4. Forest plots for all 6 lead variants from the non-stratified AD XWAS.** Because the XWAS followed a model for random XCI, the reported odds ratios correspond to a 50% probability of the allele being active, assuming there is no escape from XCI.

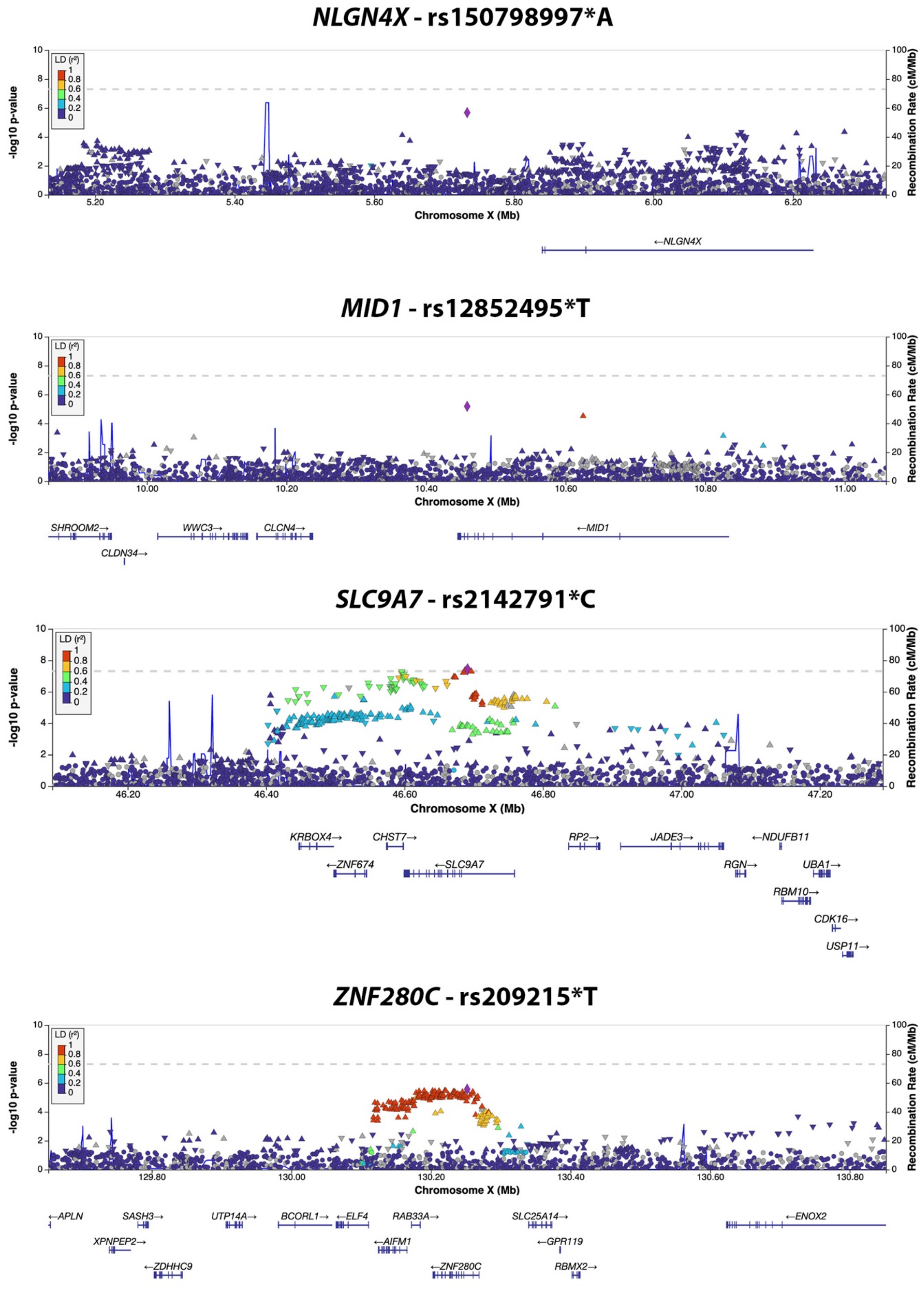


**eFigure 5 (part 1). Locus zoom plots for all 6 lead variants from the non-stratified AD XWAS**. Purple markers indicate the lead variant per locus. Linkage Disequilibrium (LD) is shown for European ancestry.

***ADGRG4* - rs5930938*T**

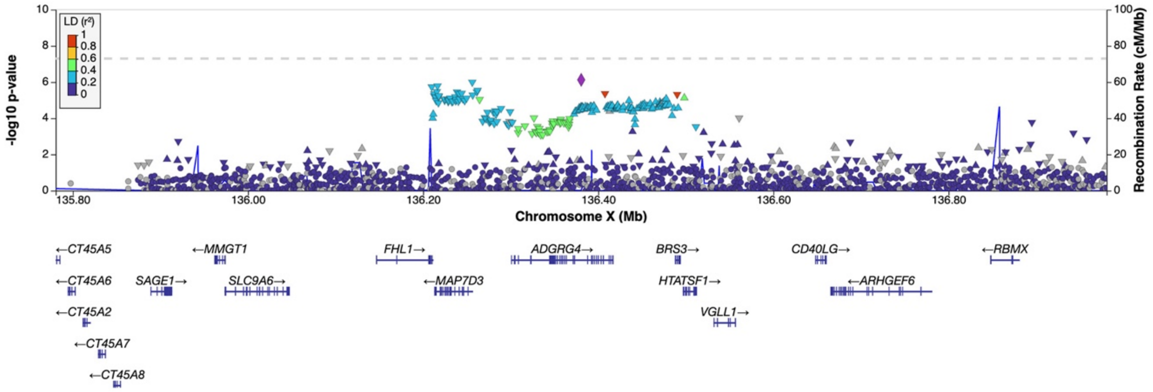


***MTM1* - rs146964414*T**

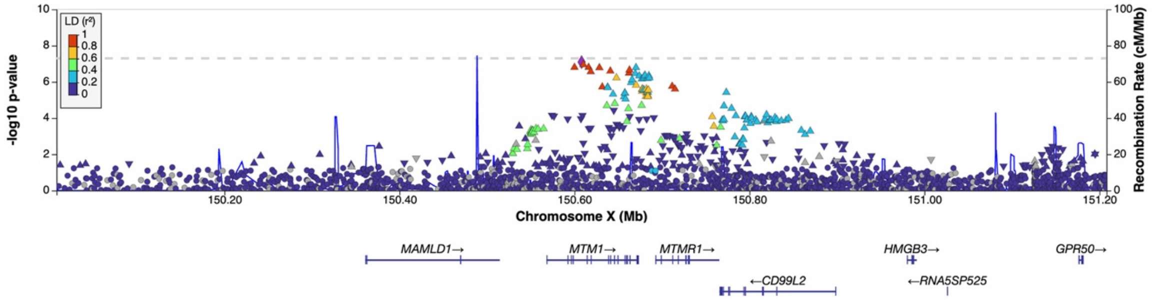


**eFigure 5 (part 2). Locus zoom plots for all 6 lead variants from the non-stratified AD XWAS**. Purple markers indicate the lead variant per locus. Linkage Disequilibrium (LD) is shown for European ancestry

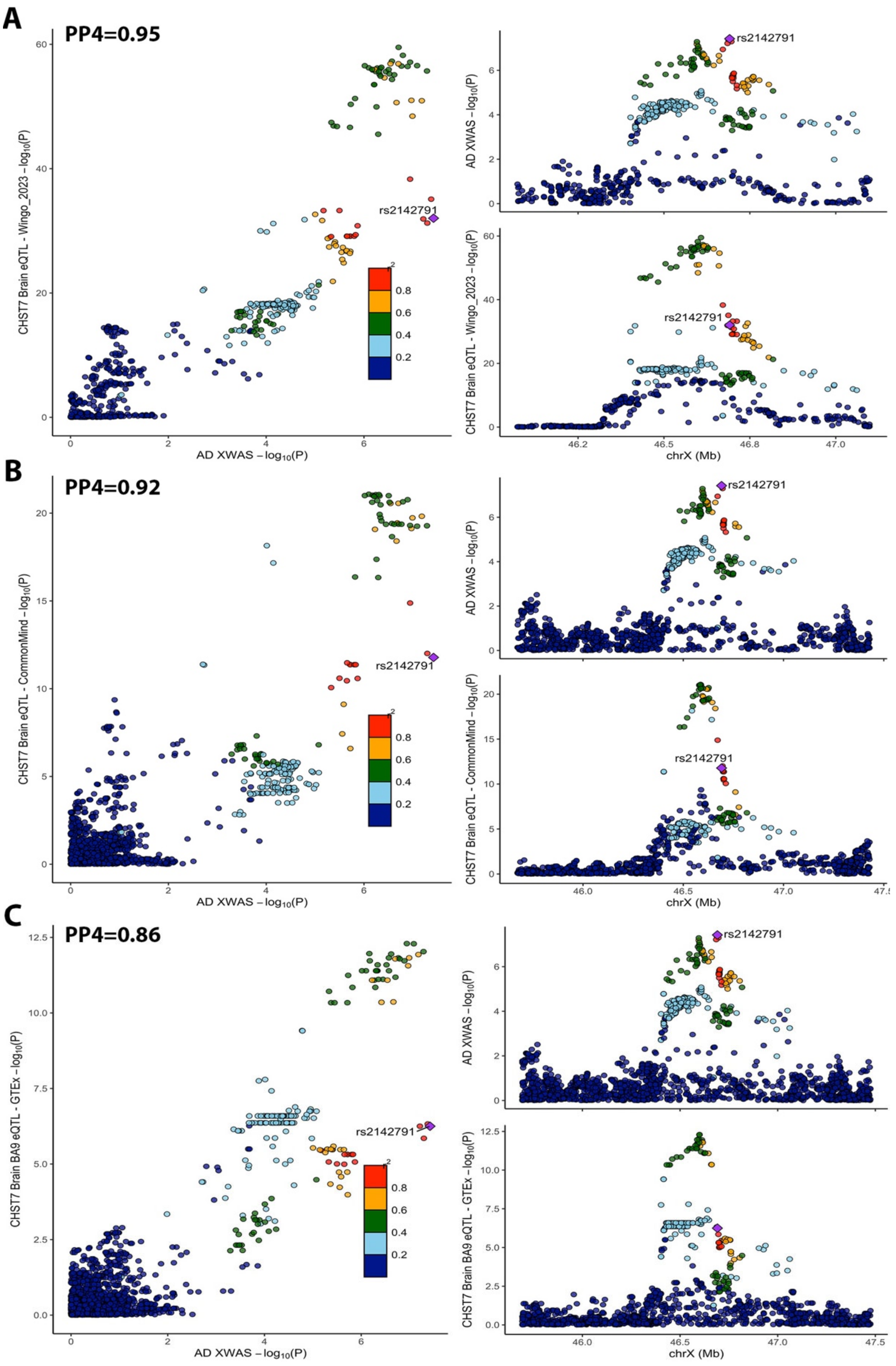


**eFigure 6. *CHST7* Colocalization plots.** For GTEx, only the colocalization with best PP4 in brain (that also had a significant QTL) is visualized. For other brain datasets, colocalizations with PP4>0.7 are visualized.

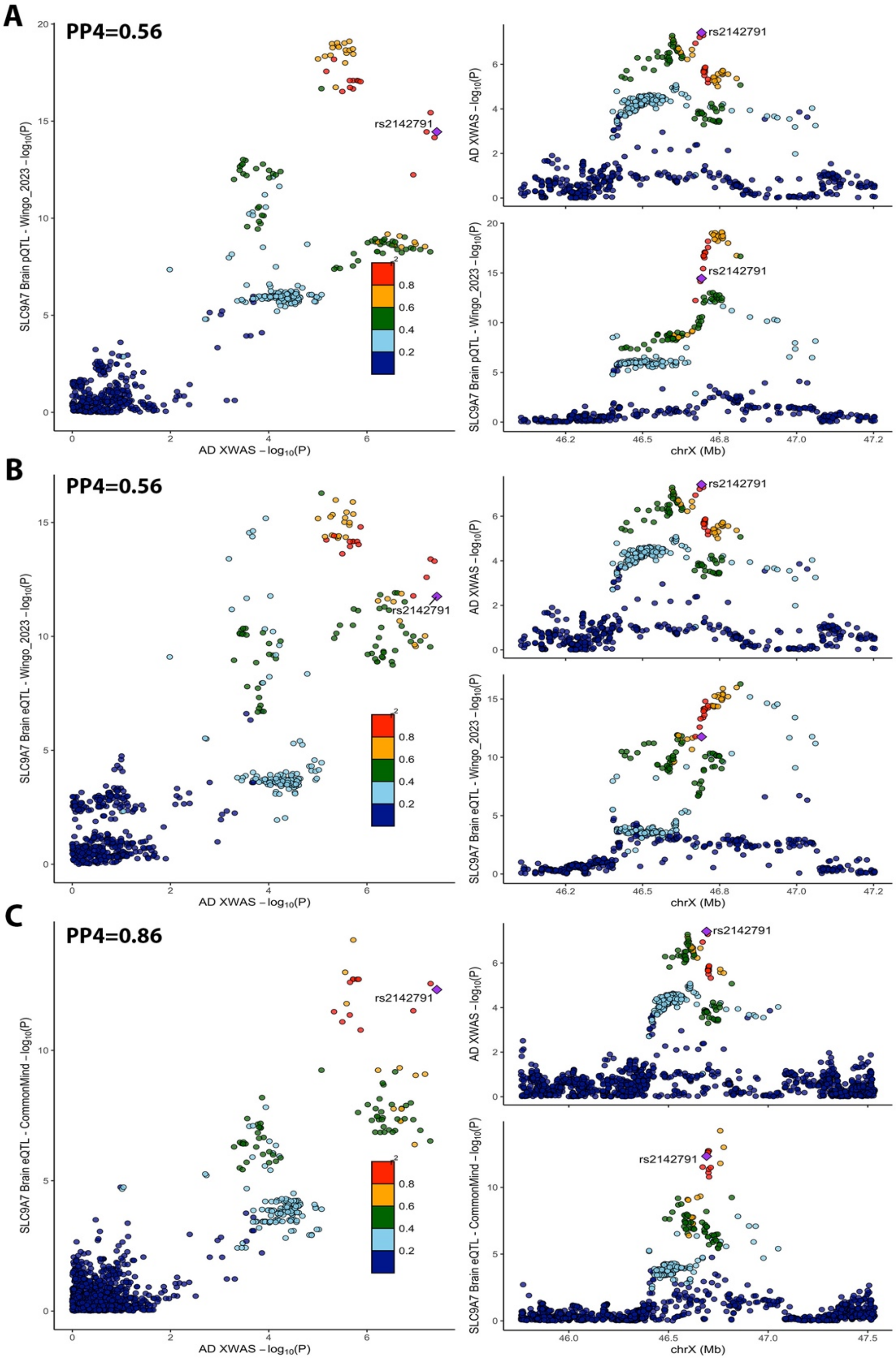


**eFigure 7 (part1). *SLC9A7* colocalization plots.** For GTEx, only the colocalization with best PP4 in brain and non-brain tissues are respectively visualized. All other brain colocalizations with a relaxed PP4>0.4 threshold are visualized, as well as the colocalization with best PP4 in monocytes.

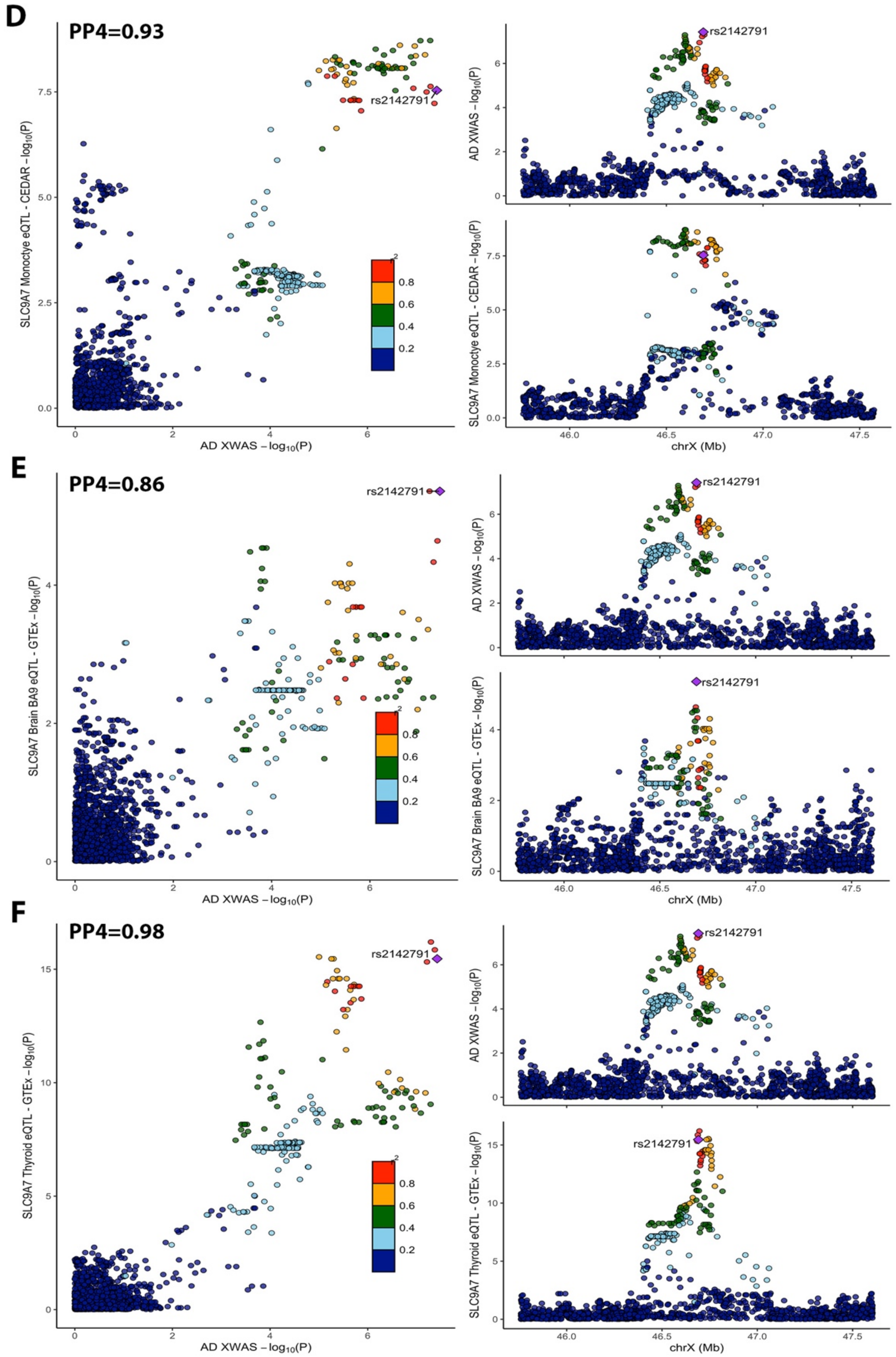


**eFigure 7 (part2). *SLC9A7* colocalization plots.** For GTEx, only the colocalization with best PP4 in brain and non-brain tissues are respectively visualized. All other brain colocalizations with a relaxed PP4>0.4 threshold are visualized, as well as the colocalization with best PP4 in monocytes.

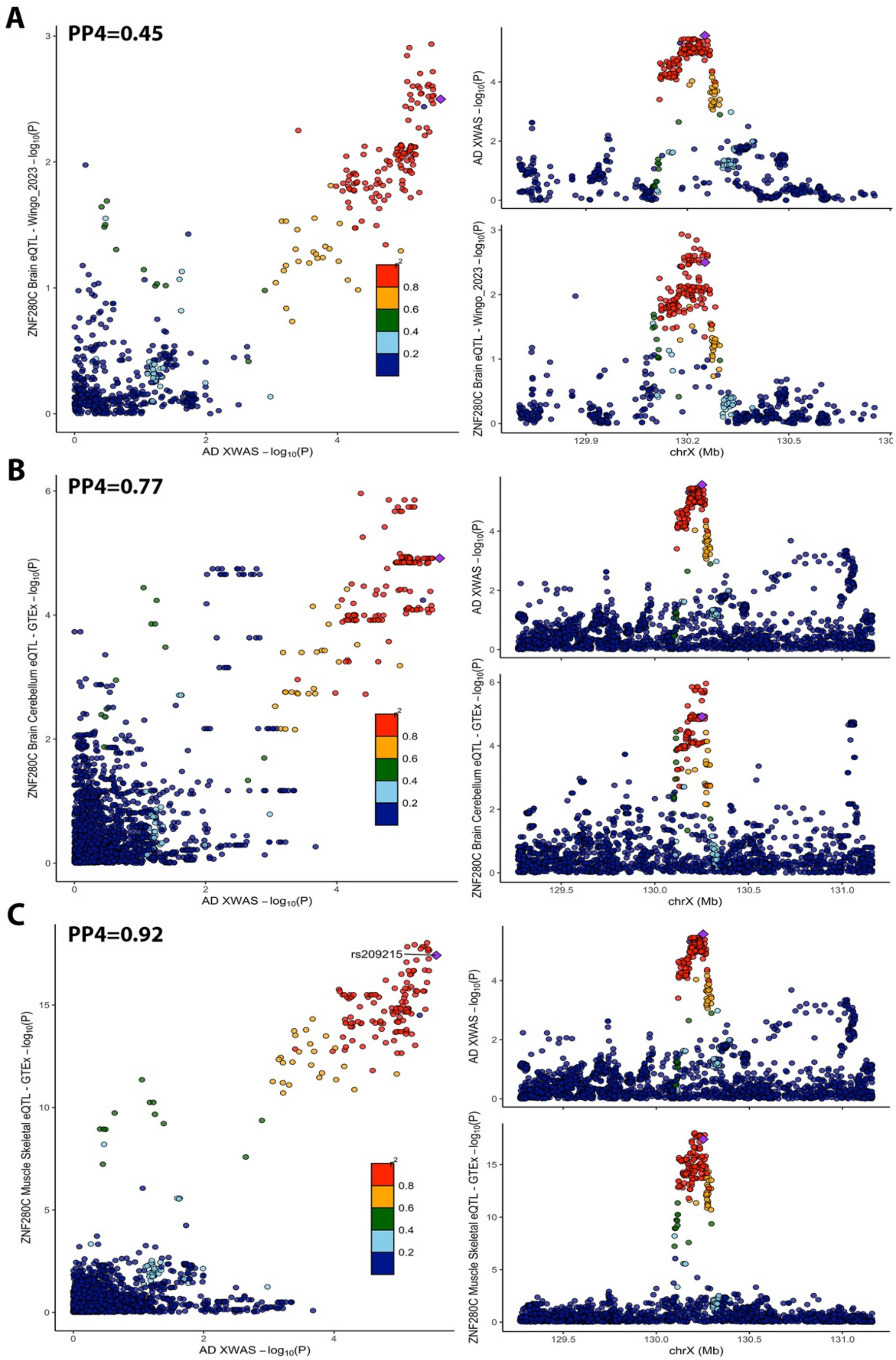


**eFigure 8. *ZNF280C* colocalization plots.** For GTEx, only the colocalizations with best PP4 in brain and non-brain tissues are respectively visualized. Additionally the colocalization in Brain eQTL data from Wingo et al. 2023, passing relaxed PP4>0.4 threshold, is visualized.

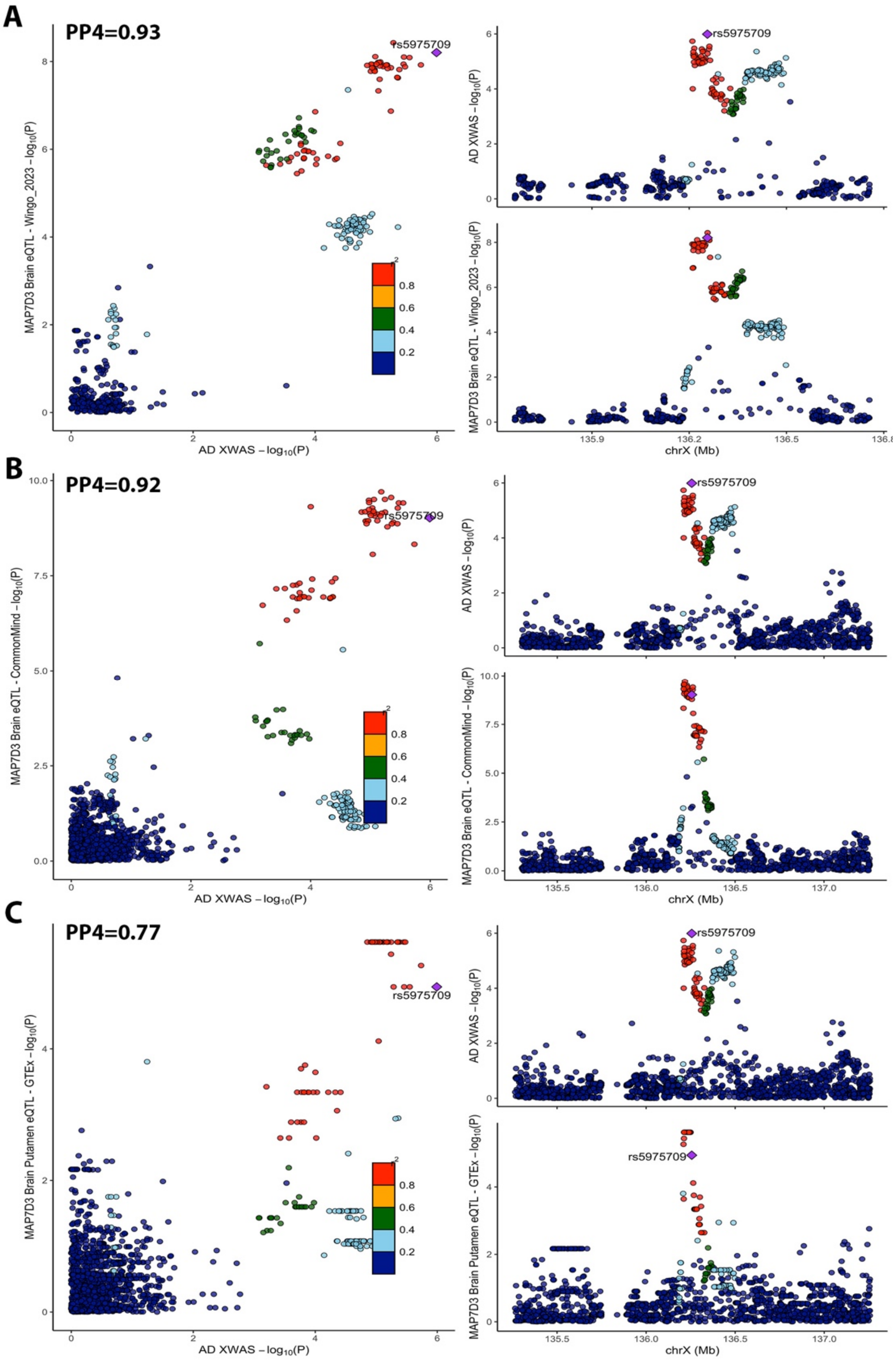


**eFigure 9.** ***MAP7D3*** **colocalization plots (part 1).** For GTEx, only the colocalizations with best PP4 in brain and non-brain tissues are respectively visualized. For other brain datasets, colocalizations with PP4>0.7 are visualized.

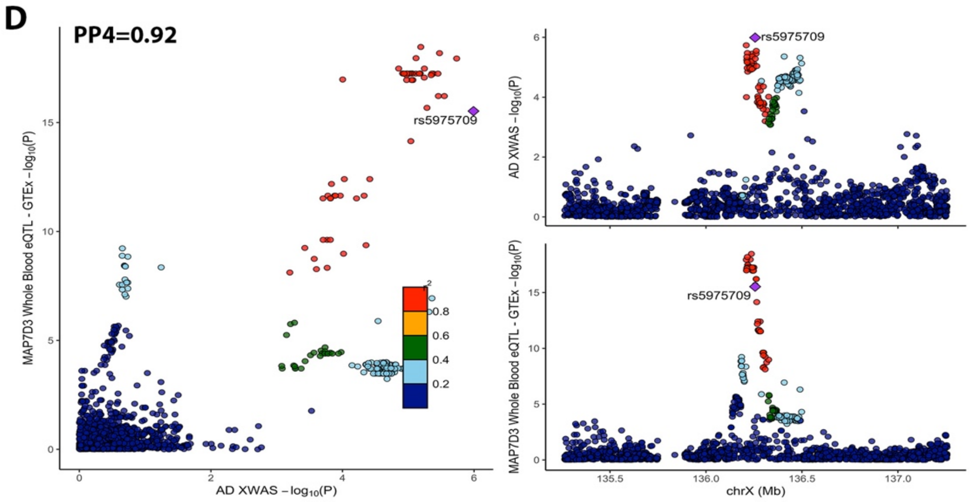


**eFigure 9.** ***MAP7D3*** **colocalization plots (part 2).** For GTEx, only the colocalizations with best PP4 in brain and non-brain tissues are respectively visualized. For other brain datasets, colocalizations with PP4>0.7 are visualized.

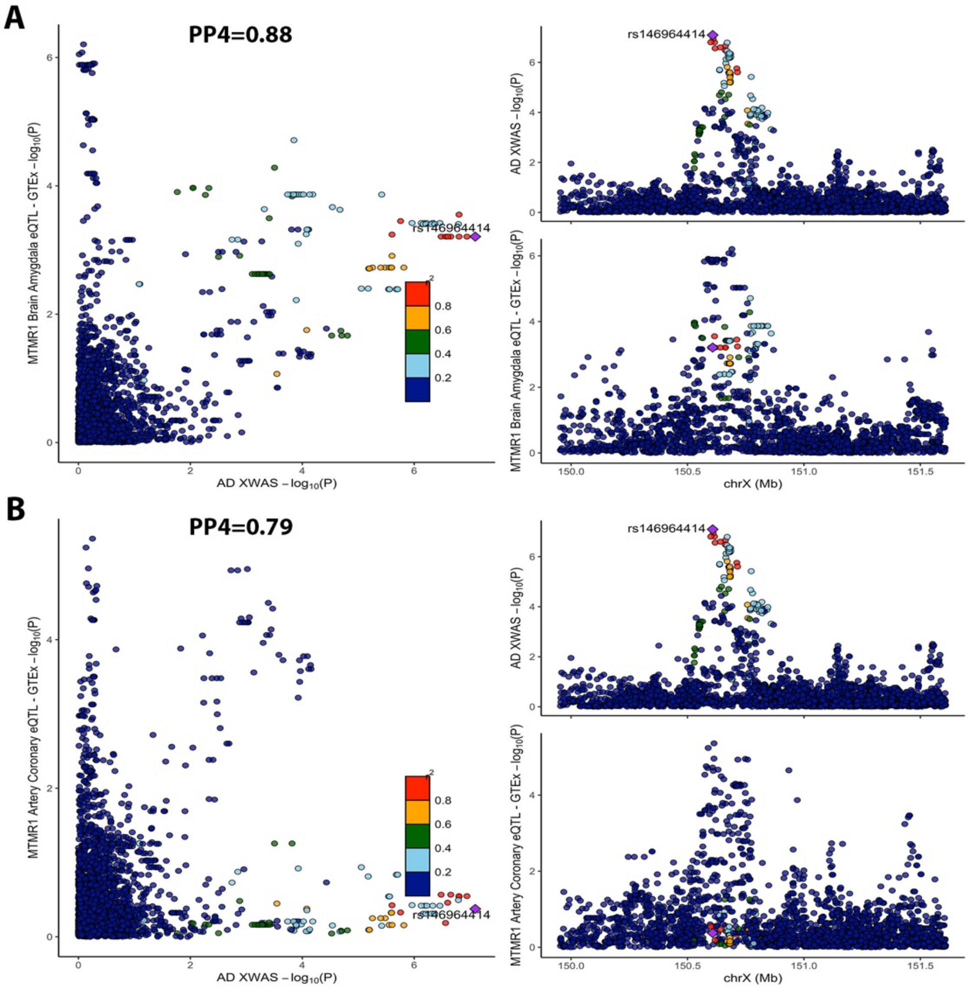


**eFigure 10.** ***MTMR1*** **colocalization plots.** The two colocalizations with PP4>0.7 are visualized. Although PP4 values were >0.7, visually, the plots suggest there is uncertainty in the colocalization with a second independent QTL signal appearing to be present.

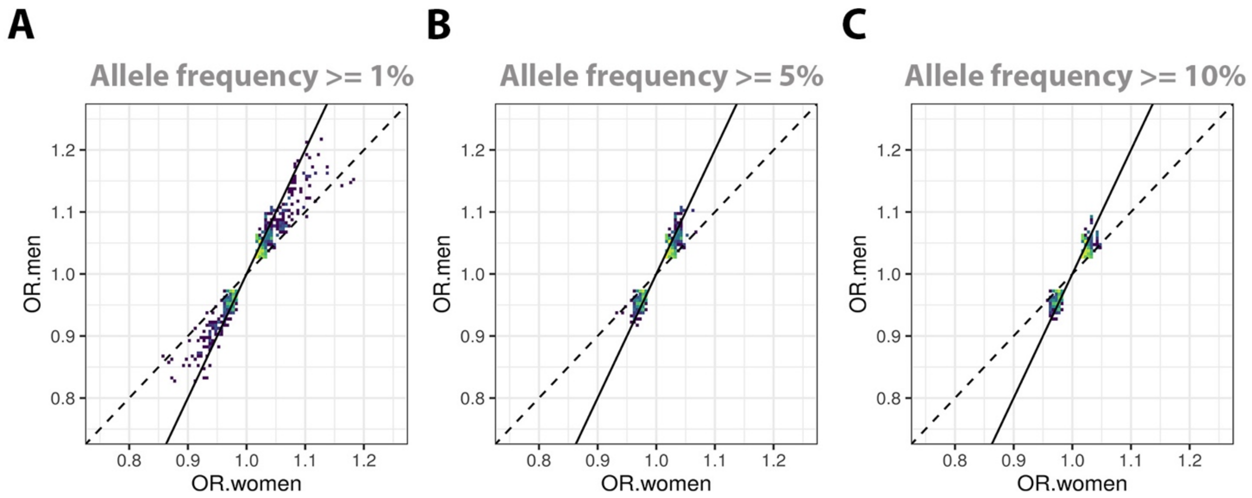


**eFigure 11. Evaluation of escape from XCI in sex-stratified AD XWAS.** Scatter density plots compare beta coefficients from men and women for prioritized variants. Intensity increases from dark blue to bright yellow. Beta coefficients from men were scaled to corresponded to a 100% probability of association. Lines with slope=1 (dashed) and slope=2 (solid) are plotted for reference and respectively indicate expectations for escape and no escape from XCI (similar approach as in Sidorenko et al. 2019[55]; for escape from XCI, the expectation is that beta coefficients from men and women are consistent). The prioritized variants had P<0.05 in men and P<0.10 in women (to balance out lower power in women under the presence of random XCI)[55], concordant effect directions across men and women, and allele frequencies **(A)** >= 1%, **(B)** >= 5%, **(C)** >= 10% (these variants are more likely to include true associations and notably include local association signals on common variant loci in Table 1). Overall, some common variants fall on or close to slope=1, suggesting they escape XCI with regard to AD risk. Theoretically, it would be the most specific to evaluate sex-specificity in data with clinically confirmed cases only (ADGC+ADSP), but given the small effect sizes or low frequencies of the lead variants, it was reasoned that the best evaluation would be based on the largest available sample size (ADGC+ADSP+UKB+MVP).

*Abbreviations: OR.women; Odds Ratio in women; OR.men; Odds Ratio in men.*

## eTables.

**eTable 1. Overview of genotyping platforms across all available AD-related genetic data**

| Cohort/Study | Genotyping Platform | Cohort-Platform ID | Sample count | Data Repository |
|---|---|---|---|---|
| ACT | Illumina Human 660W-Quad | ACT | 2790 | NIAGADS (NG00034) / dbGaP (phs000234) |
| ADC1 | Illumina Human 660W-Quad | ADC1 | 2731 | NIAGADS (NG00022) / NACC |
| ADC2 | Illumina Human 660W-Quad | ADC2 | 928 | NIAGADS (NG00023) / NACC |
| ADC3 | Illumina Human OmniExpress | ADC3 | 1526 | NIAGADS (NG00024) / NACC |
| ADC4 | Illumina Human OmniExpress | ADC4 | 1054 | NIAGADS (NG00068) / NACC |
| ADC5 | Illumina Human OmniExpress | ADC5 | 1224 | NIAGADS (NG00069) / NACC |
| ADC6 | Illumina Human OmniExpress | ADC6 | 1333 | NIAGADS (NG00070) / NACC |
| ADC7 | Illumina Infinium Human OmniExpressExome | ADC7 | 1462 | NIAGADS (NG00071) / NACC |
| ADDNEUROMED | Illumina Human 610-Quad | ADM_Q | 315 | Synapse AddNeuroMed (syn4907804) |
| | Illumina Human OmniExpress | ADM_O | 329 | Synapse AddNeuroMed (syn4907804) |
| ADNI | Illumina Human 610-Quad | ADNI_1 | 757 | LONI ADNI |
| | Illumina Human OmniExpress | ADNI_2 | 361 | LONI ADNI |
| | Illumina Global Screening Array (GSA) | ADNI_3 | 327 | LONI ADNI |
| | Illumina Omni 2.5 | ADNI_O25 | 812 | LONI ADNI |
| | Whole Genome Sequencing - Illumina | ADNI_WGS | 812 | LONI ADNI |
| ADNI-DOD | Illumina Human OmniExpress | ADNI_DOD | 204 | LONI ADNIDOD |
| ADSP WGS | Whole Genome Sequencing | ADSP_WGS | 16906 | NIAGADS DSS (NG00067.v5) / NACC |
| GenADA | Affymetrix 500K | GSK | 1571 | dbGaP (phs000219) |
| NIA-LOAD | Illumina Human 610-Quad | LOAD | 5220 | NIAGADS (NG00020) |
| MAYO | Illumina Human Hap300 | MAYO_1 | 2099 | Synapse AMP-AD (syn5591675) / NIAGADS (NG00029) |
| MAYO2 | Illumina Omni 2.5 | MAYO_2 | 314 | Synapse AMP-AD (syn5550404) |
| MIRAGE | Illumina Human CNV370-Duo | MIRAGE_370 | 397 | NIAGADS (NG00031) |
| | Illumina Human 610-Quad | MIRAGE_610 | 1105 | NIAGADS (NG00031) |
| OHSU | Illumina Human CNV370-Duo | OHSU | 647 | NIAGADS (NG00017) |
| ROSMAP | Affymetrix GeneChip 6.0 - Broad Institute | ROSMAP_1B | 1126 | RADC Rush (contact:Gregory_Klein@rush.edu) / Synapse AMP-AD |
| | Affymetrix GeneChip 6.0 - TGen | ROSMAP_1T | 582 | RADC Rush (contact:Gregory_Klein@rush.edu) / Synapse AMP-AD |
| | Illumina Human OmniExpress 12 - Chop | ROSMAP_2C | 382 | RADC Rush (contact:Gregory_Klein@rush.edu) / Synapse AMP-AD |

| | | | | |
|---|---|---|---|---|
| | Illumina Multi-Ethnic - BU | ROSMAP_3BU | 494 | RADC Rush (contact:Gregory_Klein@rush.edu) |
| TARCC | Affymetrix 6.0 | TARCC | 625 | NIAGADS (NG00097) |
| | Illumina Multi-Ethnic – BU | TARCC_full | 2718 | TARCC (contact: Bruce.Jones@UTSouthwestern.edu) |
| TGEN2 | Affymetrix 6.0 | TGEN | 1599 | NIAGADS (NG00028) |
| UPITT | Illumina Human Omni1-Quad | UPITT | 2440 | NIAGADS (NG00026) |
| | Illumina Human 1M-Duo, Illumina 1M | UVM_A | 1153 | NIAGADS (NG00042) |
| UM/VU/MSSM | Affymetrix 6.0 | UVM_B | 864 | NIAGADS (NG00042) |
| | Illumina Human 550K. Illumina Human 610-Quad | UVM_C | 445 | NIAGADS (NG00042) |
| WASHU | Illumina Human 610-Quad | WASHU_1 | 670 | NIAGADS (NG00030) |
| WASHU2 | Illumina Human OmniExpress | WASHU_2 | 235 | NIAGADS (NG00087) |
| WHICAP | Illumina Human OmniExpress | WHICAP | 647 | NIAGADS (NG00093) |

**eTable 2. Overview of ADSP available through NIAGADS DSS (NG00067).**

| Study | Accession Number | Related Datasets |
|---|---|---|
| Accelerating Medicines Partnership- Alzheimer's Disease (AMP-AD) | sa000011 | NG00067 – ADSP Umbrella |
| Cache County Study | sa000014 | NG00067 – ADSP Umbrella |
| University of Pittsburgh- Kamboh WGS | sa000012 | NG00067 – ADSP Umbrella |
| CurePSP and Tau Consortium PSP WGS | sa000016 | NG00067 – ADSP Umbrella |
| NIH, CurePSP and Tau Consortium PSP WGS | sa000015 | NG00067 – ADSP Umbrella |
| UCLA Progressive Supranuclear Palsy | sa000017 | NG00067 – ADSP Umbrella |
| NACC Genentech WGS | sa000013 | NG00067 – ADSP Umbrella |
| Alzheimer's Disease Sequencing Project (ADSP) | sa000001 | NG00067 – ADSP Umbrella |
| Alzheimer's Disease Neuroimaging Initiative (ADNI) | sa000002 | NG00067 – ADSP Umbrella |
| Alzheimer's Disease Genetics Consortium: African Americans (ADGC AA) | sa000003 | NG00067 – ADSP Umbrella |
| The Familial Alzheimer Sequencing (FASe) project | sa000004 | NG00067 – ADSP Umbrella |
| Brkanac – Family-based genome scan for AAO of LOAD | sa000005 | NG00067 – ADSP Umbrella |
| HIHG Miami Families with AD | sa000006 | NG00067 – ADSP Umbrella |
| Washington Heights/Inwood Columbia Aging Project (WHICAP) | sa000007 | NG00067 – ADSP Umbrella |
| Charles F. and Joanne Knight Alzheimer's Disease Research Center (Knight ADRC) | sa000008 | NG00067 – ADSP Umbrella |
| Corticobasal degeneration Study (CBD) | sa000009 | NG00067 – ADSP Umbrella |
| Progressive Supranuclear Palsy Study (PSP) | sa000010 | NG00067 – ADSP Umbrella |

**eTable 3. Overview of participant demographics.**

| Dataset | | Diagnosis | | Pathology | | Sex | Age |
|---|---|---|---|---|---|---|---|
| Name | Participants after QC (N) | Type | (N (%)) | Available (N (%)) | AD Path. (N (%)) | Female (N (%)) | Age (Mean (SD)) |
| **ADGC §** | 23,120 | CN | 11,582 (50.1 %) | 1,359 (11.7 %) | 237 (17.4 %) | 6,930 (59.8 %) | 77.8 (8.9) |
| | | clinical-AD | 11,538 (49.9 %) | 4,018 (34.8 %) | 4,018 (100 %) | 6,940 (60.1 %) | 74.3 (7.7) |
| **ADSP §** | 6,487 | CN | 2,944 (45.4 %) | 595 (20.2 %) | 43 (7.2 %) | 1,789 (60.8 %) | 81.6 (6.6) |
| | | clinical-AD | 3,543 (54.6 %) | 2,009 (56.7 %) | 2,009 (100 %) | 2,020 (57.0 %) | 76.7 (8.3) |
| **UKB ¶** | 433,124 | CN | 379,520 (87.6 %) | 0 (0.0 %) | 0 (0.0 %) | 207,233 (54.6 %) | 75.3 (9.9) |
| | | registry-AD | 2,050 (0.5 %) | 0 (0.0 %) | 0 (0.0 %) | 1,054 (51.4 %) | 70.2 (5.3) |
| | | proxy-ADD | 51,554 (11.9 %) | 0 (0.0 %) | 0 (0.0 %) | 39,155 (75.9 %) | 84.2 (6.3) |
| **FinnGen ‡** | 412,181 | CN | 396,564 (96.2 %) | 0 (0.0 %) | 0 (0.0 %) | 223,435 (53.3 %) | 63.0 (-) ‡ |
| | | registry-AD | 15,617 (3.8 %) | 0 (0.0 %) | 0 (0.0 %) | 6,875 (44.0 %) | 78.3 (-) ‡ |
| **MVP-1** | 117,120 | CN | 93,696 (80.0 %) | 0 (0.0 %) | 0 (0.0 %) | 3,514 (3.8 %) | 77.3 (7.3) |
| | | registry-ADD | 23,424 (20.0 %) | 0 (0.0 %) | 0 (0.0 %) | 665 (3.8 %) | 81.6 (7.9) |
| **MVP-2 ¶ †** | 160,252 | CN | 129,420 (80.8 %) | 0 (0.0 %) | 0 (0.0 %) | 123,839 (95.7 %) | 70.6 (11.6) |
| | | proxy-ADD | 30,832 (19.2 %) | 0 (0.0 %) | 0 (0.0 %) | 29,792 (96.6 %) | 73.6 (8.5) |
| **Meta-analysis** | 1,152,284 | CN | 1,013,726 (88.0 %) | 1,954 (0.2 %) | 280 (14.3 %) | 577,902 (57.0 %) | 70.1 (-) |
| | | Any-AD/ADD | 138,558 (12.0 %) | 6,027 (4.3 %) | 6,027 (100 %) | 86,501 (62.4 %) | 79.4 (-) |

*Abbreviations: CN, cognitively normal; AD, Alzheimer's disease; ADD, Alzheimer's disease-and-dementia; N, number; QC, quality control; SD, standard deviation.*

§ Across ADGC and ADSP, 40% of clinically diagnosed cases were additionally verified to have Alzheimer's disease pathology.

¶ In UKB and MVP-2 reported sex for cases was based on the sex-specificity of proxy and health registry case status, and for controls based on the sex status of the most informative (i.e. older age) parent versus offspring. When there was no sex-specificity, the sex counts were divided.

‡ Age information was not directly available in FinnGen. For controls, it was inferred from a recent research article on FinnGen[48], and for cases, it was determined using the FinnGen endpoint browser (https://r10.risteys.finngen.fi/)

† In MVP-2, most offspring were males for which paternal phenotypes have no bearing, so the analyses were mainly centered on materal proxy phenotypes. In the small subset of offspring that were women (for which both paternal phenotypes have bearing), all women with a maternal proxy-ADD phenotype also had a paternal proxy-ADD phenotype. Age for parents was not available, but all subjects were at least 45 years old at last visit, such that on average parents would be expected to be at least 60 years of age. The reported age in the table is the subject/offspring age and thus does not directly reflect parental age.

**eTable 4. Overview of variant counts in ADGC cohorts with SNP arrays**.

| Cohort-Platform ID | No. variants pre-QC | No. variants post-QC | No. variants after imputation (Rsq>=0.3) |
|---|---|---|---|
| ACT | 13355 | 8044 | 1517800 |
| ADC1 | 11227 | 6486 | 1494962 |
| ADC2 | 12051 | 7147 | 943391 |
| ADC3 | 14784 | 9015 | 1207463 |
| ADC4 | 14172 | 8535 | 1047484 |
| ADC5 | 14260 | 8589 | 1102801 |
| ADC6 | 14001 | 8406 | 1154153 |
| ADC7 | 14188 | 8558 | 1186710 |
| ADM_Q | 10129 | 5975 | 476723 |
| ADM_O | 0 | - | - |
| ADNI_1 | 17681 | 8693 | 850366 |
| ADNI_2 | 17707 | 10559 | 624153 |
| ADNI_3 | 31770 | 15601 | 626454 |
| ADNI_O25 | 55208 | 29503 | 932111 |
| ADNI_DOD | 17502 | 10484 | 416377 |
| GSK | 27380 | 3801 | 809771 |
| LOAD | 14927 | 8665 | 1688085 |
| MAYO_1 | 8906 | 5071 | 1277082 |
| MAYO_2 | 54563 | 22622 | 617560 |
| MIRAGE_370 | 8457 | 4883 | 583471 |
| MIRAGE_610 | 14565 | 8433 | 926878 |
| MTC | 14841 | 9061 | 822239 |
| OHSU | 11208 | 5857 | 770890 |
| ROSMAP_1B | 26992 | 15509 | 993056 |
| ROSMAP_1T | 0 | - | - |
| ROSMAP_2C | 14976 | 9052 | 653947 |
| ROSMAP_3BU | 17790 | 8796 | 653626 |
| TARCC | 23913 | 13150 | 819368 |
| TARCC_full | 50968 | 23661 | 1376958 |
| TGEN | 27380 | 14346 | 1067005 |
| UPITT | 15569 | 9020 | 1402980 |
| UVM_A | 10950 | 6535 | 1073412 |
| UVM_B | 23946 | 14476 | 985311 |
| UVM_C | 17230 | 10487 | 733848 |
| WASHU_1 | 5259 | 3189 | 679337 |
| WASHU_2 | 9559 | 6073 | 482874 |
| WHICAP | 14132 | 8471 | 759760 |

**eTable 5. Overview of variant counts across datasets after quality control and intersection with ADGC.**

| Dataset | No. variants prior to meta-analysis | No. variant intersecting with ADGC in meta-analysis |
|---|---|---|
| ADGC § | 437,105 | 437,105 |
| ADSP ¶ | 1,178,129 | 315,098 |
| UKB ‡ | 745,199 | 407,347 |
| FinnGen ‡ | 611,423 | 360,539 |
| MVP-1 † | 583,938 | 427,641 |
| MVP-2 † | 583,938 | 427,641 |

§ ADGC imputed cohorts were merged and variants filtered to genotyping rate >50% and minor allele count > 20, equivalent to minor allele frequencies >= 0.043%.

¶ ADSP variants (N=8,873,418) were filtered to genotyping rate >20% and minor allele count > 2 (equivalent to minor allele frequencies >= 0.015%), followed by standard, sex-specific, and ADSP-specific quality control.

‡ UKB and Finngen variants underwent cohort-specific QC and were then filtered to imputation scores > 0.3 and effect allele frequencies >= 0.05%

† MVP variants underwent cohort-specific QC and were then filtered to imputation scores > 0.4 and allele frequencies >= 0.1% in the full dataset including all ancestries. The subset of European ancestry indivdiuals was then extracted for XWAS and variants were subsequently filtered to allele frequencies >= 0.05%.

**eTable 6. Phenotype scoring and rescaling approach for the UKB non-stratified AD XWAS for a random XCI model.** In the AD XWAS in UKB, any subject with a direct AD case status or a first-degree relative with ADD case status was attributed a diagnostic/phenotypic value of 1, while other individuals above the ages of 60 in either offspring or parents were attributed the value of 0. A phenotypic weight/score was then determined for cases based on the respective combination of proxy status, subject sex, and X chromosome inheritance pattern, while modeling random XCI. This score represents the anticipated reduction in estimated beta coefficients, such that the correction factor (1/score) allows rescaling onto a regular case-control effect scale. The final beta coefficient adjustment was determined by averaging correction factors across all cases.

| | Phenotype in XWAS | Phenotype Score | correction factor (1/score) | Rationale |
|---|---|---|---|---|
| **Sex-non-specific** | | | | |
| Woman self-AD | 1 | 1 | 1 | Direct association so no correction factor. Parental info is not considered in case of self-AD |
| Woman with mother AD | 1 | 0.25 | 4 | Women have 1 X chromosome from two of the mother X chromosomes, sharing 50% of their genetic information with the mother. There is X chromosome inactivation in the mother, thus phenotye score = (1/2)/2. |
| Woman with father AD | 1 | 0.5 | 2 | Women have 1 X chromsome from father, sharing 50% of their genetic information with the father. There is no X inactivation in the father, thus phenotype score = 1/2. |
| Woman with mother AD & father AD | 1 | 0.75 | 1.33 | Combination of above scores. |
| Man self-AD | 1 | 1 | 1 | Direct association so no correction factor. Parental info is not considered in case of self-AD |
| Man with mother AD | 1 | 0.5 | 2 | Men have only 1 X chromosome from mother, sharing 100% of their genetic information with the mother. There is X chromosome inactivation in mother, thus phenotype score = 1/2. |
| Man with father AD | - | - | - | Paternal phenotype in men is not considered, since men don't inherit X chromsome from their father. |
| Man with sibling AD | 1 | 0.375 | 2.67 | If sibling sex is not known, which is the case in UK Biobank, we take the average of brother/sister scores. |
| Man with brother AD | 1 | 0.5 | 2 | Brothers inherit their X chromosome from their mother, such that the brothers share 50% genetic information. The X chromosome in the brother is active, thus phenotye score = 1/2. |
| Man with sister AD | 1 | 0.25 | 4 | The man shares 50% of his X chromosome genetic information with the sister. The sister also has an X chromosome from the father that the man doesn't. There is X chromsome inactivation in the sister, thus phenotye score = ((1/2)+0)/2. |
| Woman with sibling AD | 1 | 0.4375 | 2.29 | If sibling sex is not known, which is the case in UK Biobank, we take the average of brother/sister scores. |
| Woman with brother AD | 1 | 0.5 | 2 | The woman shares 50% of her X chromosome genetic information with the brother. The X chromosome in the brother is active, thus the phenotype score = 1/2. |
| Woman with sister AD | 1 | 0.375 | 2.67 | Both the woman and sister inherit the same X chromosome from their father (100%) and share 50% of their mother's X chromosome genetic information. There is X chromosome inactivation in the sister, thus phenotype score = (1+1/2)/2. |
| Woman with mother AD & sibling AD | 1 | 0.6875 | 1.45 | Combination of above scores. |
| Woman with father AD & sibling AD | 1 | 0.9375 | 1.07 | Combination of above scores. |
| Woman with father AD & mother AD & sibling AD | 1 | 1 | 1 | Combination of above scores, capped at 1 |
| Man with mother AD & sibling AD | 1 | 0.875 | 1.14 | Combination of above scores. |

**eTable 7. Phenotype scoring and rescaling approach for the UKB sex-stratified AD XWAS.** Compared to eTable6, additional subjects were excluded when sex-specificity was not guaranteed (e.g. both parents had AD or one parent had AD but a sibling had AD while their sex was unknown). Notably, the beta coefficient derived in men following the current approach corresponds to an XCI model, such that the beta coefficients for a single dosage represent a 50% probability of being active. As such, the male beta coefficients are subsequently multiplied by 2 to obtain beta coefficients corresponding to a genotype encoding of 0/1 (i.e. removing the XCI adjustment).

| | Phenotype in XWAS | Phenotype Score | correction factor (1/score) | Rationale |
|---|---|---|---|---|
| **Woman-specific** | | | | |
| Woman self-AD | 1 | 1 | 1 | Direct association so no correction factor. Parental info is not considered in case of self-AD |
| Man with mother AD | 1 | 0.5 | 2 | Men have only 1 X chromosome from mother, sharing 100% of their genetic information with the mother. There is X chromosome inactivation in mother, thus phenotype score = 1/2. |
| Woman with mother AD | 1 | 0.25 | 4 | Women have 1 X chromosome from two of the mother X chromosomes, sharing 50% of their genetic information with the mother. There is X chromosome inactivation in the mother, thus phenotye score = (1/2)/2. |
| no AD | 0 | 0 | - | Controls have no AD and ages>60. Parents and siblings also don't have AD. Either parents or offspring are >60y of age. |
| **Man-specific** | | | | |
| Man self-AD | 1 | 1 | 1 | Direct association so no correction factor. Parental info is not considered in case of self-AD |
| Man with father AD | - | - | - | Paternal phenotype in men is not considered, since men don't inherit X chromsome from their father. |
| Woman with father AD | 1 | 0.5 | 2 | Women have 1 X chromsome from father, sharing 50% of their genetic information with the father. There is no X inactivation in the father, thus phenotype score = 1/2. |
| no AD | 0 | 0 | - | Controls have no AD and ages>60. Parents and siblings also don't have AD. Either parents or offspring are >60y of age. |

**eTable 8. Genetic colocalization with quantitative trait locus data: Extension of Table 2 without collapsing results from overlapping tissues.** Evidence for colocalization was considered at colocalization posterior probability (PP4)>0.7 (bolded). The table presents PP4 results and is restricted to genes and datasets/tissues where at least one colocalization reached PP4>0.7. Bolded entries with an asterisk (*) indicate the lead variant was also a significant QTL in the respective data/tissue. Missing entries indicate that no QTL data were available.

| Locus | Gene with COLOC support (PP4 > 0.7) | Number of times prioritized (PP4 > 0.7) | Number of times prioritized (PP4 > 0.7) - unique data | pQTL_Brain_Wingo2023 | eQTL_Brain_Wingo2023 | eQTL_Brain_CommonMind | eQTL_monocytes_naive_Fairfax2014 | eQTL_monocytes_IFN24_Fairfax2014 | eQTL_monocytes_LPS2_Fairfax2014 | eQTL_monoctyes_CEDAR | eQTL_GTEx_Brain_Amygdala | eQTL_GTEx_Brain_Anterior_cingulate_cortex_BA24 | eQTL_GTEx_Brain_Caudate_basal_ganglia | eQTL_GTEx_Brain_Cerebellum | eQTL_GTEx_Brain_Frontal_Cortex_BA9 | eQTL_GTEx_Brain_Hypothalamus | eQTL_GTEx_Brain_Putamen_basal_ganglia | eQTL_GTEx_Whole_Blood | eQTL_GTEx_Adipose_Subcutaneous | eQTL_GTEx_Adipose_Visceral_Omentum | eQTL_GTEx_Adrenal_Gland | eQTL_GTEx_Artery_Aorta | eQTL_GTEx_Artery_Coronary | eQTL_GTEx_Artery_Tibial | eQTL_GTEx_Breast_Mammary_Tissue | eQTL_GTEx_Cells_Cultured_fibroblasts | eQTL_GTEx_Esophagus_Gastroesophageal_Junction | eQTL_GTEx_Esophagus_Mucosa | eQTL_GTEx_Esophagus_Muscularis | eQTL_GTEx_Heart_Left_Ventricle | eQTL_GTEx_Lung | eQTL_GTEx_Muscle_Skeletal | eQTL_GTEx_Nerve_Tibial | eQTL_GTEx_Pancreas | eQTL_GTEx_Pituitary | eQTL_GTEx_Skin_Not_Sun_Exposed_Suprapubic | eQTL_GTEx_Skin_Sun_Exposed_Lower_leg | eQTL_GTEx_Small_Intestine_Terminal_Ileum | eQTL_GTEx_Stomach | eQTL_GTEx_Testis | eQTL_GTEx_Thyroid |
|---|---|---|---|---|---|---|---|---|---|---|---|---|---|---|---|---|---|---|---|---|---|---|---|---|---|---|---|---|---|---|---|---|---|---|---|---|---|---|---|---|---|
| *SLC9A7* | *ENSG00000286306* | 1 | 1 | | | **0.88** | | | | | | | | | | | | | | | | | | | | | | | | | | | | | | | | | | | |
| | *KRBOX4* | 4 | 2 | | **0.77*** | 0.04 | 0.10 | 0.06 | 0.08 | 0.05 | 0.05 | 0.22 | 0.20 | **0.74** | 0.06 | 0.08 | 0.10 | 0.23 | 0.00 | 0.03 | 0.05 | 0.03 | 0.05 | 0.03 | 0.03 | 0.03 | 0.36 | 0.07 | **0.92** | 0.03 | 0.03 | 0.03 | 0.04 | 0.24 | 0.13 | 0.46 | 0.23 | 0.07 | 0.06 | 0.08 | **0.76** |
| | *CHST7* | 10 | 3 | | **0.95*** | **0.92*** | 0.27 | 0.18 | 0.16 | 0.15 | **0.79** | **0.87** | **0.93** | **0.81*** | **0.86*** | **0.79*** | **0.79*** | 0.20 | 0.00 | 0.07 | 0.16 | **0.71** | 0.32 | 0.13 | 0.03 | 0.40 | 0.04 | 0.04 | 0.10 | 0.07 | 0.05 | 0.07 | 0.11 | 0.22 | 0.04 | 0.03 | 0.06 | 0.05 | 0.06 | 0.07 | 0.59 |
| | *SLC9A7* | 12 | 4 | 0.56 | 0.56 | **0.86*** | **0.92*** | **0.93*** | **0.93*** | **0.93*** | 0.08 | 0.15 | 0.05 | 0.05 | 0.64 | 0.06 | 0.05 | 0.39 | 0.02 | **0.96*** | 0.13 | 0.63 | 0.49 | 0.57 | **0.79** | **0.90*** | 0.58 | 0.66 | 0.56 | **0.94*** | 0.02 | 0.69 | 0.60 | 0.64 | 0.05 | 0.10 | 0.67 | **0.77** | **0.85** | 0.08 | **0.98*** |
| | *RP2* | 1 | 1 | **0.89*** | 0.04 | 0.07 | 0.05 | 0.50 | 0.06 | 0.05 | 0.13 | 0.05 | 0.07 | 0.04 | 0.10 | 0.07 | 0.05 | 0.03 | 0.03 | 0.03 | 0.09 | 0.04 | 0.05 | 0.02 | 0.03 | 0.00 | 0.03 | 0.03 | 0.08 | 0.04 | 0.03 | 0.04 | 0.03 | 0.06 | 0.04 | 0.02 | 0.14 | 0.06 | 0.04 | 0.05 | 0.02 |
| | *JADE3* | 2 | 2 | | **0.70*** | 0.07 | 0.04 | 0.06 | 0.10 | 0.43 | 0.10 | 0.06 | 0.10 | 0.05 | 0.11 | 0.08 | 0.51 | 0.06 | 0.06 | 0.09 | 0.08 | 0.66 | 0.08 | 0.04 | 0.32 | 0.06 | 0.11 | **0.76** | 0.18 | 0.11 | 0.26 | 0.02 | 0.03 | 0.03 | 0.12 | 0.04 | 0.04 | 0.21 | 0.03 | 0.06 | 0.54 |
| | *UBA1* | 1 | 1 | 0.05 | 0.04 | **0.93*** | 0.13 | 0.07 | 0.09 | 0.12 | 0.12 | 0.07 | 0.05 | 0.05 | 0.05 | 0.06 | 0.09 | 0.07 | 0.03 | 0.03 | 0.05 | 0.03 | 0.05 | 0.03 | 0.05 | 0.03 | 0.04 | 0.03 | 0.03 | 0.09 | 0.04 | 0.04 | 0.00 | 0.04 | 0.05 | 0.10 | 0.03 | 0.05 | 0.04 | 0.05 | 0.00 |
| | *ELK1* | 1 | 1 | | 0.00 | 0.04 | 0.11 | **0.86** | 0.06 | 0.08 | 0.05 | 0.04 | 0.33 | 0.05 | 0.10 | 0.18 | 0.09 | 0.03 | 0.02 | 0.04 | 0.16 | 0.03 | 0.04 | 0.03 | 0.03 | 0.02 | 0.03 | 0.11 | 0.03 | 0.03 | 0.03 | 0.02 | 0.02 | 0.06 | 0.07 | 0.04 | 0.03 | 0.05 | 0.03 | 0.05 | 0.04 |
| *ZNF280C* | *ELF4* | 1 | 1 | | | 0.05 | 0.01 | 0.01 | 0.16 | 0.06 | 0.05 | 0.06 | 0.03 | 0.11 | 0.03 | 0.03 | 0.04 | 0.02 | 0.10 | 0.06 | 0.02 | 0.02 | 0.07 | 0.00 | **0.78*** | 0.02 | 0.04 | 0.01 | 0.30 | 0.37 | 0.02 | 0.59 | 0.03 | 0.04 | 0.05 | 0.02 | 0.02 | 0.04 | 0.02 | 0.09 | 0.03 |
| | *AIFM1* | 6 | 3 | 0.02 | 0.16 | 0.12 | 0.14 | **0.73** | 0.50 | **0.78*** | 0.04 | 0.09 | 0.07 | 0.56 | 0.05 | 0.07 | 0.08 | 0.19 | **0.74*** | 0.21 | 0.02 | 0.11 | 0.03 | 0.36 | 0.02 | 0.17 | 0.26 | 0.23 | 0.12 | 0.02 | 0.13 | 0.00 | 0.66 | **0.78** | 0.20 | **0.78*** | **0.74*** | 0.06 | 0.07 | 0.60 | 0.25 |
| | *ZNF280C* | 17 | 1 | | 0.45 | 0.09 | 0.07 | 0.04 | 0.19 | 0.04 | 0.42 | 0.21 | 0.10 | **0.77*** | 0.30 | 0.33 | 0.04 | 0.05 | **0.83*** | **0.86*** | 0.66 | **0.87*** | 0.03 | **0.89*** | 0.67 | **0.84*** | **0.79*** | **0.85*** | **0.89*** | 0.69 | **0.83*** | **0.92*** | **0.86*** | 0.48 | **0.79*** | **0.87*** | **0.84*** | 0.11 | **0.71** | 0.16 | **0.85*** |
| | *RBMX2* | 1 | 1 | | 0.00 | 0.29 | 0.04 | 0.04 | 0.04 | 0.05 | 0.04 | 0.04 | 0.04 | 0.05 | 0.07 | 0.04 | 0.04 | 0.02 | 0.00 | 0.00 | 0.02 | 0.00 | 0.02 | 0.00 | 0.04 | 0.00 | 0.03 | 0.02 | 0.06 | **0.77** | 0.02 | 0.01 | 0.00 | 0.02 | 0.04 | 0.01 | 0.02 | 0.03 | 0.02 | 0.06 | 0.04 |
| *ADGRG4* | *FHL1* | 2 | 2 | 0.12 | **0.78*** | 0.13 | **0.85** | 0.04 | 0.04 | 0.29 | 0.05 | 0.12 | 0.04 | 0.10 | 0.09 | 0.06 | 0.04 | 0.02 | 0.07 | 0.03 | 0.08 | 0.05 | 0.13 | 0.00 | 0.03 | 0.02 | 0.12 | 0.11 | 0.02 | 0.07 | 0.02 | 0.03 | 0.09 | 0.04 | 0.04 | 0.03 | 0.02 | 0.04 | 0.03 | 0.00 | 0.00 |
| | *MAP7D3* | 10 | 3 | 0.05 | **0.93*** | **0.92*** | 0.13 | 0.07 | 0.04 | 0.08 | 0.06 | 0.07 | 0.19 | 0.04 | 0.09 | 0.04 | **0.77*** | **0.92*** | 0.07 | 0.02 | **0.72*** | 0.02 | 0.06 | 0.02 | 0.04 | **0.89*** | 0.02 | 0.02 | 0.02 | **0.72** | **0.74** | **0.87*** | 0.10 | 0.23 | 0.04 | 0.39 | 0.05 | 0.46 | 0.45 | **0.89*** | 0.01 |
| | *BRS3* | 1 | 1 | | | 0.04 | 0.04 | 0.04 | 0.04 | 0.04 | 0.04 | 0.04 | | | | 0.04 | | | | | | | | | 0.03 | | | | | | | | | 0.05 | 0.03 | | | | | **0.73** | 0.27 |
| | *HTATSF1* | 2 | 1 | 0.03 | 0.05 | 0.03 | 0.03 | 0.07 | 0.05 | 0.06 | 0.04 | 0.04 | 0.04 | 0.04 | 0.04 | 0.04 | 0.04 | 0.01 | 0.59 | 0.02 | 0.02 | 0.09 | 0.03 | **0.87*** | 0.02 | 0.02 | 0.02 | 0.02 | 0.02 | 0.02 | 0.04 | 0.02 | 0.08 | 0.03 | 0.03 | 0.02 | **0.86*** | 0.04 | 0.05 | 0.68 | 0.02 |
| | *AL683813.2* | 1 | 1 | | | | | | | | 0.05 | 0.04 | 0.05 | 0.03 | 0.05 | 0.04 | 0.07 | 0.02 | 0.02 | 0.03 | 0.09 | 0.13 | 0.04 | 0.02 | 0.02 | 0.02 | 0.02 | 0.02 | 0.02 | 0.02 | 0.14 | **0.77** | 0.02 | 0.02 | 0.04 | 0.02 | 0.02 | 0.05 | 0.02 | 0.21 | 0.02 |
| *MTM1* | *MTMR1* | 2 | 1 | 0.01 | 0.05 | 0.16 | 0.18 | 0.12 | 0.12 | 0.11 | **0.88** | 0.36 | 0.08 | 0.63 | 0.11 | 0.08 | 0.57 | 0.01 | 0.00 | 0.00 | 0.43 | 0.00 | **0.79** | 0.00 | 0.00 | 0.00 | 0.00 | 0.00 | 0.00 | 0.08 | 0.00 | 0.44 | 0.00 | 0.00 | 0.00 | 0.00 | 0.00 | 0.63 | 0.00 | 0.00 | 0.00 |

**eTable 9. Frequency in cases and controls for *SLC9A7* lead variant across cohorts.**

| Dataset | Allele Frequency Controls | Allele Frequency Cases |
|---|---|---|
| ADGC | 45.75% | 46.99% |
| ADSP | 46.49% | 46.58% |
| UKB † | 45.82% | 46.13% |
| FinnGen | 46.53% | 47.08% |
| MVP-1 | 45.44% | 47.28% |
| MVP-2 † | 45.61% | 46.42% |

† Note that the use of proxy cases causes a dilution of case allele frequencies (which relates to the need to adjust beta coefficients from proxy GWAS and XWAS[63]).

## eTable 10. Evaluation of sex-specific effects and escape from X chromosome inactivation (XCI) for lead variants from non-stratified AD XWAS.

XCI escape was evaluated by dividing beta coefficients from men (100% probability of association) by beta coefficients from women similar to Sidorenko et al. 2019[55], where a ratio close to 2 suggests no escape from XCI and a ratio close to 1 suggests escape from XCI. Sex heterogeneity was evaluated with male beta coefficients scaled to correspond to genotype encoding 0/2 (rather than 0/1 when evaluating XCI escape). Theoretically, it would be the most specific to evaluate sex-specificity in data with clinically confirmed cases only (ADGC+ADSP), but given the small effect sizes or low frequencies of the lead variants, it was reasoned that the best evaluation would be based on the largest available sample size (ADGC+ADSP+UKB+MVP). We further identified if there was any prior support for XCI at each locus, focusing on prioritized genes for common variants (cf. Table 2) or nearest protein-coding genes for rare variants (*NLGN4X* & *MID1*). We consulted 4 resources across 2 published research articles:

- [1a]: Tukiainen et al. 2017 [56] Suppl.Table.1, which reviews XCI status reports from 2 prior papers.
- [1b]: Tukiainen et al. 2017 [56] Suppl.Table.13, which summarizes XCI status reports from their analyses.
- [1a]: Garieri et al. 2018 [57] Dataset S1, which reviews if XCI escape status was reported from 7 prior papers (inactive genes were not listed).
- [1b]: Garieri et al. 2018 [57] Dataset S3, which summarizes XCI status reports from their analyses, and 5 genes reported in their manuscript.

| Locus | non-stratified lead variant | EA | Women - No. subjects | Men - No. subjects | Women - EAF | Men - EAF | Women - Beta | Women - SE | Women - P-value | Men - Beta | Men - SE | Men - P-value | Sex heterogeneity P-value | Men / Women Beta Ratio | Appears to escape XCI in AD? | Prior XCI reports |
|---|---|---|---|---|---|---|---|---|---|---|---|---|---|---|---|---|
| *NLGN4X* | rs150798997 | A | 570,175 | 385,127 | 0.35% | 0.35% | **-0.346** | **0.092** | **1.78E-04** | **-0.298** | **0.121** | **0.014** | **0.074** | **0.86** | **Yes** | Summary: variable escape. [1a] - NLGN4X: variable escape; [1b] - NLGN4X: variable escape; [2a] - NLGN4X: escape; [2b] - NLGN4X: inactive |
| *MID1* | rs12852495 | T | 573,575 | 387,527 | 0.30% | 0.29% | **0.412** | **0.092** | **7.79E-06** | **0.158** | **0.128** | **0.217** | **2.96E-03** | **0.38** | **Yes** | Summary: variable escape. [1a] - MID1: variable escape & inactive; [1b] - MID1: inactive; [2a] - MID1: escape; [2b] - MID1: escape |
| *SLC9A7* | rs2142791 | C | 574,084 | 387,850 | 45.87% | 45.88% | 0.028 | 0.009 | 3.21E-03 | 0.066 | 0.013 | 6.76E-07 | 0.669 | 2.35 | No | Summary: variable escape (appears mostly inactive). [1a] - SLC9A7: inactive; [1a] - CHST7: inactive; [2b] - SLC9A7 - inactive & escape; [2b] - CHST7: escape |
| *ZNF280C* | rs209215 | T | 570,279 | 385,267 | 39.06% | 39.06% | **0.031** | **0.010** | **1.80E-03** | **0.038** | **0.014** | **5.13E-03** | **0.330** | **1.24** | **Yes** | Summary: variable escape. [1a] - ZNF280C: variable escape; [1b] - ZNF280C: inactive; [2b] - ZNF280C: inactive |
| *ADGRG4* | rs5930938 | C | 570,279 | 385,267 | 32.72% | 32.70% | **-0.038** | **0.010** | **2.03E-04** | **-0.053** | **0.014** | **2.01E-04** | **0.350** | **1.39** | **Yes** | Summary: variable escape. [1a] - MAP7D3: inactive; [1b] - MAP7D3: variable escape & inactive; [2a] - MAP7D3: escape; [2b] - MAP7D3: inactive |
| *MTM1* | rs146964414 | T | 411,736 | 387,845 | 8.03% | 8.09% | 0.045 | 0.017 | 7.84E-03 | 0.099 | 0.023 | 2.13E-05 | 0.830 | 2.20 | No | Summary: variable escape. [1a] - MTMR1: inactive; [1b] - MTMR1: inactive; [2a] - MTMR1: escape; [2b] - MTMR1: escape |

**eTable 11. Effect sizes for *SLC9A7* lead variant on *SLC9A7* expression in brain tissue.** The table reports the effect size only for datasets where colocalization with *SLC9A7* expression in brain tissue showed PP4>0.6. CommondMind QTL data was accessed through processed data from the eQTL Catalogue, which used conditional quantile normalization followed by inverse normal transformation[60]. The GTEx effect estimate represents the allelic fold change, i.e. the magnitude of expression change associated with a given genetic variant[64]. Given that *SLC9A7* expression is mainly reported to not escape XCI and similarly did not show signs of XCI escape with regard to AD (cf. **eTable10**), the reported effect sizes in the current table (evaluated under an XCI model) should reflect the effect of a genotype that has 50% probability of being active. The effect sizes should thus be doubled to reflect the effect of a fully active genotype.

| **Dataset** | **SLC9A7 - PP4** | **SLC9A7 eQTL - effect estimate [95%CI]** |
|---|---|---|
| eQTL_Brain_Frontal_Cortex_BA9 | 0.86 | 0.083813 [0.049139, 0.112831] |
| CommonMind_dorsolateral_prefontal_cortex | 0.64 | 0.219761 [0.161608, 0.277915] |

## References


1. Kunkle BW, Grenier-boley B, Sims R, et al. Genetic meta-analysis of diagnosed Alzheimer's disease identifies new risk loci and implicates Aβ, tau, immunity and lipid processing. *Nat Genetic*. 2019;51(3):413-430.

2. Bis JC, Jian X, Chen BWK, et al. Whole exome sequencing study identifies novel rare and common Alzheimer's-Associated variants involved in immune response and transcriptional regulation. *Mol Psychiatry*. 2020;25:1859-1875. doi:10.1038/s41380-018-0112-7

3. Beecham GW, Bis JC, Martin ER, et al. The Alzheimer's disease sequencing project: Study design and sample selection. *Neurol Genet*. 2017;3:e194. doi:10.1212/NXG.0000000000000194

4. Wang M, Beckmann ND, Roussos P, et al. The Mount Sinai cohort of large-scale genomic, transcriptomic and proteomic data in Alzheimer's disease. *Sci Data*. 2018;5:180185. doi:10.1038/sdata.2018.185

5. De Jager PL, Ma Y, McCabe C, et al. A multi-omic atlas of the human frontal cortex for aging and Alzheimer's disease research. *Sci Data*. 2018;5:180142. doi:10.1038/sdata.2018.142

6. Mckhann G, Drachman D, Folstein M, Katzman R, Price D, Mckhann G. Clinical Diagnosis of Alzheimer's Disease: Report of the NINCDS-ADRDA Work Group Under the Auspices of Derpartment of Health and Human Services Task Force on Alzheimer's Disease. *Neurology*. 1984;34:27-28. doi:10.1212/01.wnl.0000400650.92875.cf

7. NIAGADS. NG00067 – ADSP Umbrella. Published 2021. Accessed November 1, 2021. https://dss.niagads.org/datasets/ng00067/

8. Bell CC. DSM-IV: Diagnostic and Statistical Manual of Mental Disorders. *JAMA*. 1994;272(10):828-829.

9. American Psychiatric Association. Diagnostic and Statistical Manual of Mental Disorders 5th edn. *American Psychiatric Association*. Published online 2013.

10. American Psychiatric Association. Diagnostic and Statistical Manual of Mental Disorders 4th edn. *American Psychiatric Association*. Published online 1994.

11. Hughes CP, Berg L, Danziger WL, Coben LA, Martin RL. A new clinical scale for the staging of dementia. *British Journal of Psychiatry*. 1982;140(6):566-572. doi:10.1192/bjp.140.6.566

12. Braak H, Braak E. Neuropathological stageing of Alzheimer-related changes. *Acta Neuropathol*. 1991;82:239-259. doi:10.1109/ICINIS.2015.10

13. Hyman BT, Trojanowski JQ. Editorial on Consensus Recommendations for the Postmortem Diagnosis of Alzheimer Disease from the National Institute on Aging and the Reagan Institute

Working Group on Diagnositic Criteria for the Neuropathological Assessment of Alzheimr Disease. *J Neuropathol Exp Neurol*. 1997;56(10):1095-1097.

14. Folstein MF, Folstein SE, McHugh PR. “Mini-Mental State” A Practical Method for Grading the Cognitive State of Patients for the Clinician. *J Psychiatr Res*. 1975;12:189-198. doi:10.3744/snak.2003.40.2.021

15. Roccaforte WH, Burke WJ, Bayer BL, Wengel SP. Validation of a Telephone Version of the Mini-Mental State Examination. *J Am Geriatr Soc*. 1992;40:697-702.

16. McKhann GM, Knopman DS, Chertkow H, et al. The diagnosis of dementia due to Alzheimer’s disease: Recommendations from the National Institute on Aging- Alzheimer’s Association workgroups on diagnostic guidelines for Alzheimer’s disease. *Alzheimers Dement*. 2011;7(3):263-269. doi:10.1016/j.jalz.2011.03.005.

17. Albert MS, DeKosky ST, Dickson D, et al. The diagnosis of mild cognitive impairment due to Alzheimer’s disease: Recommendations from the National Institute on Aging- Alzheimer’s Association workgroups on diagnostic guidelines for Alzheimer’s disease. *Alzheimers Dement*. 2011;7(3):270-279. doi:10.1016/j.jalz.2011.03.008.

18. Hyman BT, Phelps CH, Beach TG, et al. National Institute on Aging – Alzheimer’s Association guidelines for the neuropathologic assessment of Alzheimer’s disease. *Alzheimer’s & Dementia*. 2012;8(1):1-13. doi:10.1016/j.jalz.2011.10.007

19. Weintraub S, Salmon D, Mercaldo N, et al. The Alzheimer’s Disease Centers’ Uniform Data Set (UDS) The Neuropsychologic Test Battery. *Alzheimer Dis Assoc Disord*. 2009;23(2):91-101.

20. Beekly DL, Ramos EM, Lee WW, et al. The National Alzheimer’s Coordinating Center (NACC) Database: The Uniform Data Set. *Alzheimer Dis Assoc Disord*. 2007;21(3):249-258. doi:10.1097/WAD.0b013e318142774e

21. Besser LM, Kukull WA, Teylan MA, et al. The revised national Alzheimer’s coordinating center’s neuropathology form-available data and new analyses. *J Neuropathol Exp Neurol*. 2018;77(8):717-726. doi:10.1093/jnen/nly049

22. Mirra SS, Heyman A, McKeel D, et al. The Consortium to Establish a Registry for Alzheimer’s Disease (CERAD). Part II. Standardization of the neuropathologic assessment of Alzheimer’s disease. *Neurology*. 1991;41(4):479-486. doi:10.1212/WNL.41.4.479

23. Morris JC, Weintraub S, Chui HC, et al. The Uniform Data Set (UDS): Clinical and cognitive variables and descriptive data from Alzheimer disease centers. *Alzheimer Dis Assoc Disord*. 2006;20(4):210-216. doi:10.1097/01.WAD.0000213865.09806.92

24. Weintraub S, Besser L, Dodge HH, et al. Version 3 of the Alzheimer Disease Centers' Neuropsychological Test Battery in the Uniform Data Set (UDS). *Alzheimer Dis Assoc Disord*. 2018;32(1):10-17. doi:10.1097/WAD.0000000000000223

25. Bennett DA, Schneider JA, Buchman AS, De Leon CM, Bienias JL, Wilson RS. The rush memory and aging project: Study design and baseline characteristics of the study cohort. *Neuroepidemiology*. 2005;25(4):163-175. doi:10.1159/000087446

26. Bennett DA, Wilson RS, Schneider JA, et al. Natural history of mild cognitive impairment in older persons. *Neurology*. 2002;59(2):198-205. doi:10.1212/WNL.59.2.198

27. Schneider JA, Arvanitakis Z, Bang W, Bennett DA. Mixed brain pathologies account for most dementia cases in community-dwelling older persons. *Neurology*. 2007;69(24):2197-2204. doi:10.1212/01.wnl.0000271090.28148.24

28. Hinrichs AS, Karolchik D, Baertsch R, et al. The UCSC Genome Browser Database: update 2006. *Nucleic Acids Res*. 2006;34:590-598. doi:10.1093/nar/gkj144

29. Chang CC, Chow CC, Tellier LCAM, Vattikuti S, Purcell SM, Lee JJ. Second-generation PLINK: Rising to the challenge of larger and richer datasets. *Gigascience*. 2015;4(7). doi:10.1186/s13742-015-0047-8

30. Le Guen Y, Belloy ME, Napolioni V, et al. A novel age-informed approach for genetic association analysis in Alzheimer's disease. *Alzheimers Res Ther*. 2021;13:72. 10.1186/s13195-021-00808-5

31. Karczewski KJ, Francioli LC, Tiao G, et al. The mutational constraint spectrum quantified from variation in 141,456 humans. *Nature*. 2020;581(7809):434-443. doi:10.1038/s41586-020-2308-7

32. Leung YY, Valladares O, Chou YF, et al. VCPA: Genomic variant calling pipeline and data management tool for Alzheimer's Disease Sequencing Project. *Bioinformatics*. 2019;35(10):1768-1770. doi:10.1093/bioinformatics/bty894

33. GATK team. GATK Best Practices Workflows. Accessed January 31, 2021. https://gatk.broadinstitute.org/hc/en-us/articles/360035894751

34. Belloy ME, Le Guen Y, Eger SJ, Napolioni V, Greicius MD, He Z. A fast and robust strategy to remove variant-level artifacts in Alzheimer disease sequencing project data. *Neurol Genet*. 2022;8(5). doi:10.1212/NXG.0000000000200012

35. Manichaikul A, Mychaleckyj JC, Rich SS, Daly K, Sale M, Chen WM. Robust relationship inference in genome-wide association studies. *Bioinformatics*. 2010;26(22):2867-2873. doi:10.1093/BIOINFORMATICS/BTQ559

36. Chen CY, Pollack S, Hunter DJ, Hirschhorn JN, Kraft P, Price AL. Improved ancestry inference using weights from external reference panels. *Bioinformatics*. 2013;29(11):1399-1406. doi:10.1093/bioinformatics/btt144

37. Auton A, Abecasis GR, Altshuler DM, et al. A global reference for human genetic variation. *Nature*. 2015;526(7571):68-74. doi:10.1038/nature15393

38. Lambert CA, Connelly CF, Madeoy J, Qiu R, Olson M V., Akey JM. Highly punctuated patterns of population structure on the X chromosome and implications for African evolutionary history. *Am J Hum Genet*. 2010;86(1):34-44. doi:10.1016/J.AJHG.2009.12.002

39. Conomos MP, Miller MB, Thornton TA. Robust inference of population structure for ancestry prediction and correction of stratification in the presence of relatedness. *Genet Epidemiol*. 2015;39(4):276-293. doi:10.1002/gepi.21896

40. Taliun D, Harris DN, Kessler MD, et al. Sequencing of 53,831 diverse genomes from the NHLBI TOPMed Program. *Nature*. 2021;590(7845):290-299. doi:10.1038/s41586-021-03205-y

41. Das S, Forer L, Schönherr S, et al. Next-generation genotype imputation service and methods. *Nat Genet*. 2016;48(10):1284-1287. doi:10.1038/ng.3656

42. Belloy ME, Andrews SJ, Yann ;, et al. APOE Genotype and Alzheimer Disease Risk Across Age, Sex, and Population Ancestry. *JAMA Neurol*. Published online November 6, 2023. doi:10.1001/JAMANEUROL.2023.3599

43. Loh P ru, Kichaev G, Gazal S, Schoech AP, Price AL. Mixed-model association for biobank-scale datasets. *Nat Genet*. 2018;50:906-908. doi:10.1038/s41588-018-0144-6

44. Wattmo C, Londos E, Minthon L. Risk factors that affect life expectancy in alzheimer's disease: A 15-year follow-up. *Dement Geriatr Cogn Disord*. 2014;38(5-6):286-299. doi:10.1159/000362926

45. Kuzma A, Valladares O, Cweibel R, et al. NIAGADS: The NIA Genetics of Alzheimer's Disease Data Storage Site. *Alzheimer's & Dementia*. 2016;12(11):1200-1203. doi:10.1016/j.jalz.2016.08.018

46. Bycroft C, Freeman C, Petkova D, et al. The UK Biobank resource with deep phenotyping and genomic data. *Nature*. 2018;562:203-209. doi:10.1038/s41586-018-0579-z

47. Schwartzentruber J, Cooper S, Liu JZ, et al. Genome-wide meta-analysis, fine-mapping and integrative prioritization implicate new Alzheimer's disease risk genes. *Nat Genet*. 2021;53(3):392-402. doi:10.1038/s41588-020-00776-w

48. Kurki MI, Karjalainen J, Palta P, et al. FinnGen provides genetic insights from a well-phenotyped isolated population. *Nature*. 2023;613(7944):508-518. doi:10.1038/S41586-022-05473-8

49. Mbatchou J, Barnard L, Backman J, et al. Computationally efficient whole-genome regression for quantitative and binary traits. *Nature Genetics 2021 53:7*. 2021;53(7):1097-1103. doi:10.1038/s41588-021-00870-7

50. Sherva R, Zhang R, Sahelijo N, et al. African ancestry GWAS of dementia in a large military cohort identifies significant risk loci. *Mol Psychiatry*. 2023;28(3):1293-1302. doi:10.1038/s41380-022-01890-3

51. Nguyen XMT, Whitbourne SB, Li Y, et al. Data Resource Profile: Self-reported data in the Million Veteran Program: survey development and insights from the first 850 736 participants. *Int J Epidemiol*. 2023;52(1):e1-e17. doi:10.1093/IJE/DYAC133

52. Hunter-Zinck H, Shi Y, Li M, et al. Genotyping Array Design and Data Quality Control in the Million Veteran Program. *The American Journal of Human Genetics*. 2020;106(4):535-548. doi:10.1016/J.AJHG.2020.03.004

53. Fang H, Hui Q, Lynch J, et al. Harmonizing Genetic Ancestry and Self-identified Race/Ethnicity in Genome-wide Association Studies. *Am J Hum Genet*. 2019;105(4):763-772. doi:10.1016/J.AJHG.2019.08.012

54. Mägi R, Morris AP. GWAMA: Software for genome-wide association meta-analysis. *BMC Bioinformatics*. 2010;11(1):1-6. doi:10.1186/1471-2105-11-288/TABLES/2

55. Sidorenko J, Kassam I, Kemper KE, et al. The effect of X-linked dosage compensation on complex trait variation. *Nat Commun*. 2019;10(1). doi:10.1038/s41467-019-10598-y

56. Tukiainen T, Villani AC, Yen A, et al. Landscape of X chromosome inactivation across human tissues. *Nature*. 2017;550(7675):244-248. doi:10.1038/nature24265

57. Garieri M, Stamoulis G, Blanc X, et al. Extensive cellular heterogeneity of X inactivation revealed by single-cell allele-specific expression in human fibroblasts. *Proc Natl Acad Sci U S A*. 2018;115(51):13015-13020. doi:10.1073/PNAS.1806811115

58. Aguet F, Barbeira AN, Bonazzola R, et al. The GTEx Consortium atlas of genetic regulatory effects across human tissues The Genotype Tissue Expression Consortium. *Science (1979)*. 2020;369:1318-1330. doi:10.1101/787903

59. Wingo AP, Liu Y, Gerasimov ES, et al. Sex differences in brain protein expression and disease. *Nat Med*. Published online September 1, 2023. doi:10.1038/s41591-023-02509-y

60. Kerimov N, Hayhurst JD, Peikova K, et al. A compendium of uniformly processed human gene expression and splicing quantitative trait loci. *Nat Genet*. 2021;53(September):1290-1299. doi:10.1038/s41588-021-00924-w

61. Bellenguez C, Küçükali F, Jansen IE, et al. New insights into the genetic etiology of Alzheimer’s disease and related dementias. *Nat Genet*. 2022;54(4):412-436. doi:10.1038/S41588-022-01024-Z

62. Giambartolomei C, Vukcevic D, Schadt EE, et al. Bayesian Test for Colocalisation between Pairs of Genetic Association Studies Using Summary Statistics. *PLoS Genet*. 2014;10(5):1-15. doi:10.1371/journal.pgen.1004383

63. Liu JZ, Erlich Y, Pickrell JK. Case-control association mapping by proxy using family history of disease. *Nat Genet*. 2017;49(3):325-331. doi:10.1038/ng.3766

64. Mohammadi P, Castel SE, Brown AA, Lappalainen T. Quantifying the regulatory effect size of cis-acting genetic variation using allelic fold change. *Genome Res*. 2017;27(11):1872-1884. doi:10.1101/gr.216747.116

eAppendix. Additional contributions

Acknowledgments for the use of ADSP WES and WGS data:

The Alzheimer's Disease Sequencing Project (ADSP) is comprised of two Alzheimer's Disease (AD) genetics consortia and three National Human Genome Research Institute (NHGRI) funded Large Scale Sequencing and Analysis Centers (LSAC). The two AD genetics consortia are the Alzheimer's Disease Genetics Consortium (ADGC) funded by NIA (U01 AG032984), and the Cohorts for Heart and Aging Research in Genomic Epidemiology (CHARGE) funded by NIA (R01 AG033193), the National Heart, Lung, and Blood Institute (NHLBI), other National Institute of Health (NIH) institutes and other foreign governmental and non-governmental organizations. The Discovery Phase analysis of sequence data is supported through UF1AG047133 (to Drs. Schellenberg, Farrer, Pericak-Vance, Mayeux, and Haines); U01AG049505 to Dr. Seshadri; U01AG049506 to Dr. Boerwinkle; U01AG049507 to Dr. Wijsman; and U01AG049508 to Dr. Goate and the Discovery Extension Phase analysis is supported through U01AG052411 to Dr. Goate, U01AG052410 to Dr. Pericak-Vance and U01 AG052409 to Drs. Seshadri and Fornage.

Sequencing for the Follow Up Study (FUS) is supported through U01AG057659 (to Drs. PericakVance, Mayeux, and Vardarajan) and U01AG062943 (to Drs. Pericak-Vance and Mayeux). Data generation and harmonization in the Follow-up Phase is supported by U54AG052427 (to Drs. Schellenberg and Wang). The FUS Phase analysis of sequence data is supported through U01AG058589 (to Drs. Destefano, Boerwinkle, De Jager, Fornage, Seshadri, and Wijsman), U01AG058654 (to Drs. Haines, Bush, Farrer, Martin, and Pericak-Vance), U01AG058635 (to Dr. Goate), RF1AG058066 (to Drs. Haines, Pericak-Vance, and Scott), RF1AG057519 (to Drs. Farrer and Jun), R01AG048927 (to Dr. Farrer), and RF1AG054074 (to Drs. Pericak-Vance and Beecham).

The ADGC cohorts include: Adult Changes in Thought (ACT) (UO1 AG006781, UO1 HG004610, UO1 HG006375, U01 HG008657), the Alzheimer's Disease Centers (ADC) ( P30 AG019610, P30 AG013846, P50 AG008702, P50 AG025688, P50 AG047266, P30 AG010133, P50 AG005146, P50 AG005134, P50 AG016574, P50 AG005138, P30 AG008051, P30 AG013854, P30 AG008017, P30 AG010161, P50 AG047366, P30 AG010129, P50 AG016573, P50 AG016570, P50 AG005131, P50 AG023501, P30 AG035982, P30 AG028383, P30 AG010124, P50 AG005133, P50 AG005142, P30 AG012300, P50 AG005136, P50 AG033514, P50 AG005681, and P50 AG047270), the Chicago Health and Aging Project (CHAP) (R01 AG11101, RC4 AG039085, K23 AG030944), Indianapolis Ibadan (R01 AG009956, P30 AG010133), the Memory and Aging Project (MAP) ( R01 AG17917), Mayo Clinic (MAYO) (R01 AG032990, U01 AG046139, R01 NS080820, RF1 AG051504, P50 AG016574), Mayo Parkinson's Disease controls (NS039764, NS071674, 5RC2HG005605), University of Miami (R01 AG027944, R01 AG028786, R01 AG019085, IIRG09133827, A2011048), the Multi-Institutional Research in Alzheimer's Genetic Epidemiology Study (MIRAGE) (R01 AG09029, R01 AG025259), the National Cell Repository for Alzheimer's Disease (NCRAD) (U24 AG21886), the National Institute on Aging Late Onset Alzheimer's Disease Family Study (NIA- LOAD) (R01 AG041797), the Religious Orders Study (ROS) (P30 AG10161, R01 AG15819), the Texas Alzheimer's Research and Care Consortium (TARCC) (funded by the Darrell K Royal Texas Alzheimer's Initiative), Vanderbilt University/Case Western Reserve University (VAN/CWRU) (R01

AG019757, R01 AG021547, R01 AG027944, R01 AG028786, P01 NS026630, and Alzheimer's Association), the Washington Heights-Inwood Columbia Aging Project (WHICAP) (RF1 AG054023), the University of Washington Families (VA Research Merit Grant, NIA: P50AG005136, R01AG041797, NINDS: R01NS069719), the Columbia University HispanicEstudio Familiar de Influencia Genetica de Alzheimer (EFIGA) (RF1 AG015473), the University of Toronto (UT) (funded by Wellcome Trust, Medical Research Council, Canadian Institutes of Health Research), and Genetic Differences (GD) (R01 AG007584). The CHARGE cohorts are supported in part by National Heart, Lung, and Blood Institute (NHLBI) infrastructure grant HL105756 (Psaty), RC2HL102419 (Boerwinkle) and the neurology working group is supported by the National Institute on Aging (NIA) R01 grant AG033193.

The CHARGE cohorts participating in the ADSP include the following: Austrian Stroke Prevention Study (ASPS), ASPS-Family study, and the Prospective Dementia Registry-Austria (ASPS/PRODEM-Aus), the Atherosclerosis Risk in Communities (ARIC) Study, the Cardiovascular Health Study (CHS), the Erasmus Rucphen Family Study (ERF), the Framingham Heart Study (FHS), and the Rotterdam Study (RS). ASPS is funded by the Austrian Science Fond (FWF) grant number P20545-P05 and P13180 and the Medical University of Graz. The ASPS-Fam is funded by the Austrian Science Fund (FWF) project I904),the EU Joint Programme - Neurodegenerative Disease Research (JPND) in frame of the BRIDGET project (Austria, Ministry of Science) and the Medical University of Graz and the Steiermärkische Krankenanstalten Gesellschaft. PRODEM-Austria is supported by the Austrian Research Promotion agency (FFG) (Project No. 827462) and by the Austrian National Bank (Anniversary Fund, project 15435. ARIC research is carried out as a collaborative study supported by NHLBI contracts (HHSN268201100005C, HHSN268201100006C, HHSN268201100007C, HHSN268201100008C, HHSN268201100009C, HHSN268201100010C, HHSN268201100011C, and HHSN268201100012C). Neurocognitive data in ARIC is collected by U01 2U01HL096812, 2U01HL096814, 2U01HL096899, 2U01HL096902, 2U01HL096917 from the NIH (NHLBI, NINDS, NIA and NIDCD), and with previous brain MRI examinations funded by R01-HL70825 from the NHLBI. CHS research was supported by contracts HHSN268201200036C, HHSN268200800007C, N01HC55222, N01HC85079, N01HC85080, N01HC85081, N01HC85082, N01HC85083, N01HC85086, and grants U01HL080295 and U01HL130114 from the NHLBI with additional contribution from the National Institute of Neurological Disorders and Stroke (NINDS). Additional support was provided by R01AG023629, R01AG15928, and R01AG20098 from the NIA. FHS research is supported by NHLBI contracts N01-HC-25195 and HHSN268201500001I. This study was also supported by additional grants from the NIA (R01s AG054076, AG049607 and AG033040 and NINDS (R01 NS017950). The ERF study as a part of EUROSPAN (European Special Populations Research Network) was supported by European Commission FP6 STRP grant number 018947 (LSHG-CT-2006-01947) and also received funding from the European Community's Seventh Framework Programme (FP7/2007-2013)/grant agreement HEALTH-F4- 2007-201413 by the European Commission under the programme "Quality of Life and Management of the Living Resources" of 5th Framework Programme (no. QLG2-CT-2002- 01254). High-throughput analysis of the ERF data was supported by a joint grant from the Netherlands Organization for Scientific Research and the Russian Foundation for Basic Research (NWO-RFBR 047.017.043). The Rotterdam Study is funded by Erasmus Medical Center and Erasmus University, Rotterdam, the Netherlands Organization for Health Research and Development (ZonMw), the Research Institute for Diseases in the Elderly (RIDE), the Ministry of Education, Culture and

Science, the Ministry for Health, Welfare and Sports, the European Commission (DG XII), and the municipality of Rotterdam. Genetic data sets are also supported by the Netherlands Organization of Scientific Research NWO Investments (175.010.2005.011, 911-03-012), the Genetic Laboratory of the Department of Internal Medicine, Erasmus MC, the Research Institute for Diseases in the Elderly (014-93-015; RIDE2), and the Netherlands Genomics Initiative (NGI)/Netherlands Organization for Scientific Research (NWO) Netherlands Consortium for Healthy Aging (NCHA), project 050-060-810. All studies are grateful to their participants, faculty and staff. The content of these manuscripts is solely the responsibility of the authors and does not necessarily represent the official views of the National Institutes of Health or the U.S. Department of Health and Human Services.

The FUS cohorts include: the Alzheimer's Disease Centers (ADC) ( P30 AG019610, P30 AG013846, P50 AG008702, P50 AG025688, P50 AG047266, P30 AG010133, P50 AG005146, P50 AG005134, P50 AG016574, P50 AG005138, P30 AG008051, P30 AG013854, P30 AG008017, P30 AG010161, P50 AG047366, P30 AG010129, P50 AG016573, P50 AG016570, P50 AG005131, P50 AG023501, P30 AG035982, P30 AG028383, P30 AG010124, P50 AG005133, P50 AG005142, P30 AG012300, P50 AG005136, P50 AG033514, P50 AG005681, and P50 AG047270), Alzheimer's Disease Neuroimaging Initiative (ADNI) (U19AG024904), Amish Protective Variant Study (RF1AG058066), Cache County Study (R01AG11380, R01AG031272, R01AG21136, RF1AG054052), Case Western Reserve University Brain Bank (CWRUBB) (P50AG008012), Case Western Reserve University Rapid Decline (CWRURD) (RF1AG058267, NU38CK000480), CubanAmerican Alzheimer's Disease Initiative (CuAADI) (3U01AG052410), Estudio Familiar de Influencia Genetica en Alzheimer (EFIGA) (5R37AG015473, RF1AG015473, R56AG051876), Genetic and Environmental Risk Factors for Alzheimer Disease Among African Americans Study (GenerAAtions) (2R01AG09029, R01AG025259, 2R01AG048927), Gwangju Alzheimer and Related Dementias Study (GARD) (U01AG062602), Hussman Institute for Human Genomics Brain Bank (HIHGBB) (R01AG027944, Alzheimer's Association "Identification of Rare Variants in Alzheimer Disease"), Ibadan Study of Aging (IBADAN) (5R01AG009956), Mexican Health and Aging Study (MHAS) (R01AG018016), Multi-Institutional Research in Alzheimer's Genetic Epidemiology (MIRAGE) (2R01AG09029, R01AG025259, 2R01AG048927), Northern Manhattan Study (NOMAS) (R01NS29993), Peru Alzheimer's Disease Initiative (PeADI) (RF1AG054074), Puerto Rican 1066 (PR1066) (Wellcome Trust (GR066133/GR080002), European Research Council (340755)), Puerto Rican Alzheimer Disease Initiative (PRADI) (RF1AG054074), Reasons for Geographic and Racial Differences in Stroke (REGARDS) (U01NS041588), Research in African American Alzheimer Disease Initiative (REAAADI) (U01AG052410), Rush Alzheimer's Disease Center (ROSMAP) (P30AG10161, R01AG15819, R01AG17919), University of Miami Brain Endowment Bank (MBB), and University of Miami/Case Western/North Carolina A&T African American (UM/CASE/NCAT) (U01AG052410, R01AG028786).

The four LSACs are: the Human Genome Sequencing Center at the Baylor College of Medicine (U54 HG003273), the Broad Institute Genome Center (U54HG003067), The American Genome Center at the Uniformed Services University of the Health Sciences (U01AG057659), and the Washington University Genome Institute (U54HG003079).

Biological samples and associated phenotypic data used in primary data analyses were stored at Study Investigators institutions, and at the National Cell Repository for Alzheimer's Disease (NCRAD,

U24AG021886) at Indiana University funded by NIA. Associated Phenotypic Data used in primary and secondary data analyses were provided by Study Investigators, the NIA funded Alzheimer's Disease Centers (ADCs), and the National Alzheimer's Coordinating Center (NACC, U01AG016976) and the National Institute on Aging Genetics of Alzheimer's Disease Data Storage Site (NIAGADS, U24AG041689) at the University of Pennsylvania, funded by NIA This research was supported in part by the Intramural Research Program of the National Institutes of health, National Library of Medicine. Contributors to the Genetic Analysis Data included Study Investigators on projects that were individually funded by NIA, and other NIH institutes, and by private U.S. organizations, or foreign governmental or nongovernmental organizations.

An up to date acknowledgment statement can be found on the ADSP site: https://www.niagads.org/adsp/content/acknowledgement-statement.

Data collection and sharing for this project was funded by the Alzheimer's Disease Neuroimaging Initiative (ADNI) (National Institutes of Health Grant U01 AG024904) and DOD ADNI (Department of Defense award number W81XWH-12-2-0012). ADNI is funded by the National Institute on Aging, the National Institute of Biomedical Imaging and Bioengineering, and through generous contributions from the following: AbbVie, Alzheimer's Association; Alzheimer's Drug Discovery Foundation; Araclon Biotech; BioClinica, Inc.; Biogen; Bristol-Myers Squibb Company; CereSpir, Inc.; Cogstate; Eisai Inc.; Elan Pharmaceuticals, Inc.; Eli Lilly and Company; EuroImmun; F. Hoffmann-La Roche Ltd and its affiliated company Genentech, Inc.; Fujirebio; GE Healthcare; IXICO Ltd.; Janssen Alzheimer Immunotherapy Research & Development, LLC.; Johnson & Johnson Pharmaceutical Research & Development LLC.; Lumosity; Lundbeck; Merck & Co., Inc.; Meso Scale Diagnostics, LLC.; NeuroRx Research; Neurotrack Technologies; Novartis Pharmaceuticals Corporation; Pfizer Inc.; Piramal Imaging; Servier; Takeda Pharmaceutical Company; and Transition Therapeutics. The Canadian Institutes of Health Research is providing funds to support ADNI clinical sites in Canada. Private sector contributions are facilitated by the Foundation for the National Institutes of Health (www.fnih.org). The grantee organization is the Northern California Institute for Research and Education, and the study is coordinated by the Alzheimer's Therapeutic Research Institute at the University of Southern California. ADNI data are disseminated by the Laboratory for Neuro Imaging at the University of Southern California.

Additional information to include in an acknowledgment statement can be found on the LONI site: https://adni.loni.usc.edu/wp-content/uploads/how_to_apply/ADNI_Data_Use_Agreement.pdf.

The Alzheimer's Disease Genetics Consortium (ADGC) supported sample preparation, whole exome sequencing and data processing through NIA grant U01AG032984. Sequencing data generation and harmonization is supported by the Genome Center for Alzheimer's Disease, U54AG052427, and data sharing is supported by NIAGADS, U24AG041689. Samples from the National Centralized Repository for Alzheimer's Disease and Related Dementias (NCRAD), which receives government support under a cooperative agreement grant (U24 AG021886) awarded by the National Institute on Aging (NIA), were used in this study. We thank contributors who collected samples used in this study, as well as patients and their families, whose help and participation made this work possible. NIH grants supported enrollment and data collection for the individual studies including: GenerAAtions R01AG20688 (PI M.

Daniele Fallin, PhD); Miami/Duke R01 AG027944, R01 AG028786 (PI Margaret A. Pericak-Vance, PhD); NC A&T P20 MD000546, R01 AG28786-01A1 (PI Goldie S. Byrd, PhD); Case Western (PI Jonathan L. Haines, PhD); MIRAGE R01 AG009029 (PI Lindsay A. Farrer, PhD); ROS P30AG10161, R01AG15819, R01AG30146, TGen (PI David A. Bennett, MD); MAP R01AG17917, R01AG15819, TGen (PI David A. Bennett, MD). The NACC database is funded by NIA/NIH Grant U01 AG016976. NACC data are contributed by the NIA-funded ADCs: P30 AG019610 (PI Eric Reiman, MD), P30 AG013846 (PI Neil Kowall, MD), P30 AG062428-01 (PI James Leverenz, MD) P50 AG008702 (PI Scott Small, MD), P50 AG025688 (PI Allan Levey, MD, PhD), P50 AG047266 (PI Todd Golde, MD, PhD), P30 AG010133 (PI Andrew Saykin, PsyD), P50 AG005146 (PI Marilyn Albert, PhD), P30 AG062421-01 (PI Bradley Hyman, MD, PhD), P30 AG062422-01 (PI Ronald Petersen, MD, PhD), P50 AG005138 (PI Mary Sano, PhD), P30 AG008051 (PI Thomas Wisniewski, MD), P30 AG013854 (PI Robert Vassar, PhD), P30 AG008017 (PI Jeffrey Kaye, MD), P30 AG010161 (PI David Bennett, MD), P50 AG047366 (PI Victor Henderson, MD, MS), P30 AG010129 (PI Charles DeCarli, MD), P50 AG016573 (PI Frank LaFerla, PhD), P30 AG062429-01(PI James Brewer, MD, PhD), P50 AG023501 (PI Bruce Miller, MD), P30 AG035982 (PI Russell Swerdlow, MD), P30 AG028383 (PI Linda Van Eldik, PhD), P30 AG053760 (PI Henry Paulson, MD, PhD), P30 AG010124 (PI John Trojanowski, MD, PhD), P50 AG005133 (PI Oscar Lopez, MD), P50 AG005142 (PI Helena Chui, MD), P30 AG012300 (PI Roger Rosenberg, MD), P30 AG049638 (PI Suzanne Craft, PhD), P50 AG005136 (PI Thomas Grabowski, MD), P30 AG062715-01 (PI Sanjay Asthana, MD, FRCP), P50 AG005681 (PI John Morris, MD), P50 AG047270 (PI Stephen Strittmatter, MD, PhD).

This work was supported by grants from the National Institutes of Health (R01AG044546, P01AG003991, RF1AG053303, R01AG058501, U01AG058922, RF1AG058501 and R01AG057777). The recruitment and clinical characterization of research participants at Washington University were supported by NIH P50 AG05681, P01 AG03991, and P01 AG026276. This work was supported by access to equipment made possible by the Hope Center for Neurological Disorders, and the Departments of Neurology and Psychiatry at Washington University School of Medicine.

We thank the contributors who collected samples used in this study, as well as patients and their families, whose help and participation made this work possible. Members of the National Institute on Aging Late-Onset Alzheimer Disease/National Cell Repository for Alzheimer Disease (NIA-LOAD NCRAD) Family Study Group include the following: Richard Mayeux, MD, MSc; Martin Farlow, MD; Tatiana Foroud, PhD; Kelley Faber, MS; Bradley F. Boeve, MD; Neill R. Graff-Radford, MD; David A. Bennett, MD; Robert A. Sweet, MD; Roger Rosenberg, MD; Thomas D. Bird, MD; Carlos Cruchaga, PhD; and Jeremy M. Silverman, PhD.

This work was partially supported by grant funding from NIH R01 AG039700 and NIH P50 AG005136. Subjects and samples used here were originally collected with grant funding from NIH U24 AG026395, U24 AG021886, P50 AG008702, P01 AG007232, R37 AG015473, P30 AG028377, P50 AG05128, P50 AG16574, P30 AG010133, P50 AG005681, P01 AG003991, U01MH046281, U01 MH046290 and U01 MH046373. The funders had no role in study design, analysis or preparation of the manuscript. The authors declare no competing interests.

This work was supported by the National Institutes of Health (R01 AG027944, R01 AG028786 to MAPV, R01 AG019085 to JLH, P20 MD000546); a joint grant from the Alzheimer's Association (SG-14-312644) and the Fidelity Biosciences Research Initiative to MAPV; the BrightFocus Foundation (A2011048 to MAPV). NIA-LOAD Family-Based Study supported the collection of samples used in this study through NIH grants U24 AG026395 and R01 AG041797 and the MIRAGE cohort was supported through the NIH grants R01 AG025259 and R01 AG048927. We thank contributors, including the Alzheimer's disease Centers who collected samples used in this study, as well as patients and their families, whose help and participation made this work possible. Study design: HNC, BWK, JLH, MAPV; Sample collection: MLC, JMV, RMC, LAF, JLH, MAPV; Whole exome sequencing and Sanger sequencing: SR, PLW; Sequencing data analysis: HNC, BWK, KLHN, SR, MAK, JRG, ERM, GWB, MAPV; Statistical analysis: BWK, KLHN, JMJ, MAPV; Preparation of manuscript: HNC, BWK. The authors jointly discussed the experimental results throughout the duration of the study. All authors read and approved the final manuscript.

Data collection and sharing for this project was supported by the Washington Heights-Inwood Columbia Aging Project (WHICAP, PO1AG07232, R01AG037212, RF1AG054023) funded by the National Institute on Aging (NIA) and by the National Center for Advancing Translational Sciences, National Institutes of Health, through Grant Number UL1TR001873. This manuscript has been reviewed by WHICAP investigators for scientific content and consistency of data interpretation with previous WHICAP Study publications. We acknowledge the WHICAP study participants and the WHICAP research and support staff for their contributions to this study.

This work was supported by grants from the National Institutes of Health (R01AG044546, P01AG003991, RF1AG053303, R01AG058501, U01AG058922, RF1AG058501 and R01AG057777). The recruitment and clinical characterization of research participants at Washington University were supported by NIH P50 AG05681, P01 AG03991, and P01 AG026276. This work was supported by access to equipment made possible by the Hope Center for Neurological Disorders, and the Departments of Neurology and Psychiatry at Washington University School of Medicine.

We thank the contributors who collected samples used in this study, as well as patients and their families, whose help and participation made this work possible. Members of the National Institute on Aging Late-Onset Alzheimer Disease/National Cell Repository for Alzheimer Disease (NIA-LOAD NCRAD) Family Study Group include the following: Richard Mayeux, MD, MSc; Martin Farlow, MD; Tatiana Foroud, PhD; Kelley Faber, MS; Bradley F. Boeve, MD; Neill R. Graff-Radford, MD; David A. Bennett, MD; Robert A. Sweet, MD; Roger Rosenberg, MD; Thomas D. Bird, MD; Carlos Cruchaga, PhD; and Jeremy M. Silverman, PhD.

This work was supported by grants from the National Institutes of Health (R01AG044546, P01AG003991, RF1AG053303, R01AG058501, U01AG058922, RF1AG058501 and R01AG057777). The recruitment and clinical characterization of research participants at Washington University were supported by NIH P50 AG05681, P01 AG03991, and P01 AG026276. This work was supported by access to equipment made possible by the Hope Center for Neurological Disorders, and the Departments of Neurology and Psychiatry at Washington University School of Medicine.

We thank the contributors who collected samples used in this study, as well as patients and their families, whose help and participation made this work possible. Members of the National Institute on Aging Late-Onset Alzheimer Disease/National Cell Repository for Alzheimer Disease (NIA-LOAD NCRAD) Family Study Group include the following: Richard Mayeux, MD, MSc; Martin Farlow, MD; Tatiana Foroud, PhD; Kelley Faber, MS; Bradley F. Boeve, MD; Neill R. Graff-Radford, MD; David A. Bennett, MD; Robert A. Sweet, MD; Roger Rosenberg, MD; Thomas D. Bird, MD; Carlos Cruchaga, PhD; and Jeremy M. Silverman, PhD.

Mayo RNAseq Study- Study data were provided by the following sources: The Mayo Clinic Alzheimer's Disease Genetic Studies, led by Dr. Nilufer Ertekin-Taner and Dr. Steven G. Younkin, Mayo Clinic, Jacksonville, FL using samples from the Mayo Clinic Study of Aging, the Mayo Clinic Alzheimer's Disease Research Center, and the Mayo Clinic Brain Bank. Data collection was supported through funding by NIA grants P50 AG016574, R01 AG032990, U01 AG046139, R01 AG018023, U01 AG006576, U01 AG006786, R01 AG025711, R01 AG017216, R01 AG003949, NINDS grant R01 NS080820, CurePSP Foundation, and support from Mayo Foundation. Study data includes samples collected through the Sun Health Research Institute Brain and Body Donation Program of Sun City, Arizona. The Brain and Body Donation Program is supported by the National Institute of Neurological Disorders and Stroke (U24 NS072026 National Brain and Tissue Resource for Parkinson's Disease and Related Disorders), the National Institute on Aging (P30 AG19610 Arizona Alzheimer's Disease Core Center), the Arizona Department of Health Services (contract 211002, Arizona Alzheimer's Research Center), the Arizona Biomedical Research Commission (contracts 4001, 0011, 05-901 and 1001 to the Arizona Parkinson's Disease Consortium) and the Michael J. Fox Foundation for Parkinson's Research

ROSMAP- We are grateful to the participants in the Religious Order Study, the Memory and Aging Project. This work is supported by the US National Institutes of Health [U01 AG046152, R01 AG043617, R01 AG042210, R01 AG036042, R01 AG036836, R01 AG032990, R01 AG18023, RC2 AG036547, P50 AG016574, U01 ES017155, KL2 RR024151, K25 AG041906-01, R01 AG30146, P30 AG10161, R01 AG17917, R01 AG15819, K08 AG034290, P30 AG10161 and R01 AG11101.

Mount Sinai Brain Bank (MSBB)- This work was supported by the grants R01AG046170, RF1AG054014, RF1AG057440 and R01AG057907 from the NIH/National Institute on Aging (NIA). R01AG046170 is a component of the AMP-AD Target Discovery and Preclinical Validation Project. Brain tissue collection and characterization was supported by NIH HHSN271201300031C.

This study was supported by the National Institute on Aging (NIA) grants AG030653, AG041718, AG064877 and P30-AG066468.

We would like to thank study participants, their families, and the sample collectors for their invaluable contributions. This research was supported in part by the National Institute on Aging grant U01AG049508 (PI Alison M. Goate). This research was supported in part by Genentech, Inc. (PI Alison M. Goate, Robert R. Graham).

The NACC database is funded by NIA/NIH Grant U01 AG016976. NACC data are contributed by these NIA-funded ADCs: P30 AG013846 (PI Neil Kowall, MD), P50 AG008702 (PI Scott Small, MD), P50

AG025688 (PI Allan Levey, MD, PhD), P30 AG010133 (PI Andrew Saykin, PsyD), P50 AG005146 (PI Marilyn Albert, PhD), P50 AG005134 (PI Bradley Hyman, MD, PhD), P50 AG016574 (PI Ronald Petersen, MD, PhD), P30 AG013854 (PI M. Marsel Mesulam, MD), P30 AG008017 (PI Jeffrey Kaye, MD), P30 AG010161 (PI David Bennett, MD), P30 AG010129 (PI Charles DeCarli, MD), P50 AG016573 (PI Frank LaFerla, PhD), P50 AG005131 (PI Douglas Galasko, MD), P30 AG028383 (PI Linda Van Eldik, PhD), P30 AG010124 (PI John Trojanowski, MD, PhD), P50 AG005142 (PI Helena Chui, MD), P30 AG012300 (PI Roger Rosenberg, MD), P50 AG005136 (PI Thomas Grabowski, MD), P50 AG005681 (PI John Morris, MD), P30 AG028377 (Kathleen Welsh-Bohmer, PhD), and P50 AG008671 (PI Henry Paulson, MD, PhD).

Samples from the National Cell Repository for Alzheimer's Disease (NCRAD), which receives government support under a cooperative agreement grant (U24 AG21886) awarded by the National Institute on Aging (NIA), were used in this study. We thank contributors who collected samples used in this study, as well as patients and their families, whose help and participation made this work possible.

The Alzheimer's Disease Genetics Consortium supported the collection of samples used in this study through National Institute on Aging (NIA) grants U01AG032984 and RC2AG036528.

We acknowledge the generous contributions of the Cache County Memory Study participants. Sequencing for this study was funded by RF1AG054052 (PI: John S.K. Kauwe)

Acknowledgments for the use of GWAS data distributed by NIAGADS

The NIA Genetics of Alzheimer's Disease Data Storage Site (NIAGADS) is supported by a collaborative agreement from the National Institute on Aging, U24AG041689.

NG00047: The NIA supported this work through grants U01-AG032984, RC2-AG036528, U01-AG016976 (Dr Kukull); U24 AG026395, U24 AG026390, R01AG037212, R37 AG015473 (Dr Mayeux); K23AG034550 (Dr Reitz); U24-AG021886 (Dr Foroud); R01AG009956, RC2 AG036650 (Dr Hall); UO1 AG06781, UO1 HG004610 (Dr Larson); R01 AG009029 (Dr Farrer); 5R01AG20688 (Dr Fallin); P50 AG005133, AG030653 (Dr Kamboh); R01 AG019085 (Dr Haines); R01 AG1101, R01 AG030146, RC2 AG036650 (Dr Evans); P30AG10161, R01AG15819, R01AG30146, R01AG17917, R01AG15819 (Dr Bennett); R01AG028786 (Dr Manly); R01AG22018, P30AG10161 (Dr Barnes); P50AG16574 (Dr Ertekin-Taner, Dr Graff-Radford), R01 AG032990 (Dr Ertekin-Taner), KL2 RR024151 (Dr Ertekin-Taner); R01 AG027944, R01 AG028786 (Dr Pericak-Vance); P20 MD000546, R01 AG28786-01A1 (Dr Byrd); AG005138 (Dr Buxbaum); P50 AG05681, P01 AG03991, P01 AG026276 (Dr Goate); and P30AG019610, P30AG13846, U01-AG10483, R01CA129769, R01MH080295, R01AG017173, R01AG025259, R01AG33193, P50AG008702, P30AG028377, AG05128, AG025688, P30AG10133, P50AG005146, P50AG005134, P01AG002219, P30AG08051, MO1RR00096, UL1RR029893, P30AG013854, P30AG008017, R01AG026916, R01AG019085, P50AG016582, UL1RR02777, R01AG031581, P30AG010129, P50AG016573, P50AG016575, P50AG016576, P50AG016577, P50AG016570, P50AG005131, P50AG023501, P50AG019724, P30AG028383, P50AG008671, P30AG010124, P50AG005142, P30AG012300, AG010491, AG027944, AG021547, AG019757, P50AG005136 (Alzheimer Disease GeneticsConsortium [ADGC]). We thank Creighton Phelps, Stephen Synder, and Marilyn Miller from the NIA, who are ex-officio members of the ADGC. Support was also provided by the Alzheimer's Association (IIRG-08-89720 [Dr Farrer] and

IIRG-05-14147 [Dr Pericak-Vance]), National Institute of Neurological Disorders and Stroke grant NS39764, National Institute of Mental Health grant MH60451, GlaxoSmithKline, and the Office of Research and Development, Biomedical Laboratory Research Program, US Department of Veterans Affairs Administration. For the ADGC, biological samples and associated phenotypic data used in primary data analyses were stored at principal investigators' institutions and at the National Cell Repository for Alzheimer's Disease (NCRAD) at Indiana University, funded by the NIA. Associated phenotypic data used in secondary data analyses were stored at the National Alzheimer's Coordinating Center and at the NIA Alzheimer's Disease Data Storage Site at the University of Pennsylvania, funded by the NIA. Contributors to the genetic analysis data included principal investigators on projects ndividually funded by the NIA, other NIH institutes, or private entities.

Acknowledgments for other GWAS and phenotype data

NACC

The NACC database is funded by NIA/NIH Grant U01 AG016976. NACC data are contributed by the NIA-funded ADCs: P30 AG019610 (PI Eric Reiman, MD), P30 AG013846 (PI Neil Kowall, MD), P30 AG062428-01 (PI James Leverenz, MD) P50 AG008702 (PI Scott Small, MD), P50 AG025688 (PI Allan Levey, MD, PhD), P50 AG047266 (PI Todd Golde, MD, PhD), P30 AG010133 (PI Andrew Saykin, PsyD), P50 AG005146 (PI Marilyn Albert, PhD), P30 AG062421-01 (PI Bradley Hyman, MD, PhD), P30 AG062422-01 (PI Ronald Petersen, MD, PhD), P50 AG005138 (PI Mary Sano, PhD), P30 AG008051 (PI Thomas Wisniewski, MD), P30 AG013854 (PI Robert Vassar, PhD), P30 AG008017 (PI Jeffrey Kaye, MD), P30 AG010161 (PI David Bennett, MD), P50 AG047366 (PI Victor Henderson, MD, MS), P30 AG010129 (PI Charles DeCarli, MD), P50 AG016573 (PI Frank LaFerla, PhD), P30 AG062429-01(PI James Brewer, MD, PhD), P50 AG023501 (PI Bruce Miller, MD), P30 AG035982 (PI Russell Swerdlow, MD), P30 AG028383 (PI Linda Van Eldik, PhD), P30 AG053760 (PI Henry Paulson, MD, PhD), P30 AG010124 (PI John Trojanowski, MD, PhD), P50 AG005133 (PI Oscar Lopez, MD), P50 AG005142 (PI Helena Chui, MD), P30 AG012300 (PI Roger Rosenberg, MD), P30 AG049638 (PI Suzanne Craft, PhD), P50 AG005136 (PI Thomas Grabowski, MD), P30 AG062715-01 (PI Sanjay Asthana, MD, FRCP), P50 AG005681 (PI John Morris, MD), P50 AG047270 (PI Stephen Strittmatter, MD, PhD).

MARS & LATC

We thank all Minority Aging Research Study and Latino Core participants and the Rush Alzheimer's Disease Center staff. This database was funded by the NIH/NIA grants R01AG22018 (MARS) and P30AG 072975 (ADC).

GenADA

The genotypic and associated phenotypic data used in the study "Multi-Site Collaborative Study for Genotype-Phenotype Associations in Alzheimer's Disease (GenADA)" were provided by the GlaxoSmithKline, R&D Limited.

ROSMAP

ROSMAP study data were provided by the Rush Alzheimer's Disease Center, Rush University Medical Center, Chicago. Data collection was supported through funding by NIA grants P30AG10161, R01AG15819, R01AG17917, R01AG30146, R01AG36836, U01AG32984, U01AG46152, the Illinois Department of Public Health, and the Translational Genomics Research Institute.

AddNeuroMed

The AddNeuroMed data are from a public-private partnership supported by EFPIA companies and SMEs as part of InnoMed (Innovative Medicines in Europe), an Integrated Project funded by the European Union of the Sixth Framework program priority FP6-2004-LIFESCIHEALTH-5. Clinical leads responsible for data collection are Iwona Kłoszewska (Lodz), Simon Lovestone (London), Patrizia Mecocci (Perugia), Hilkka Soininen (Kuopio), Magda Tsolaki (Thessaloniki), and Bruno Vellas (Toulouse), imaging leads are Andy Simmons (London), Lars-Olad Wahlund (Stockholm) and Christian Spenger (Zurich) and bioinformatics leads are Richard Dobson (London) and Stephen Newhouse (London).

ADNI

Data collection and sharing for this project was funded by the Alzheimer's Disease Neuroimaging Initiative (ADNI) (National Institutes of Health Grant U01 AG024904) and DOD ADNI (Department of Defense award number W81XWH-12-2-0012). ADNI is funded by the National Institute on Aging, the National Institute of Biomedical Imaging and Bioengineering and through generous contributions from the following: AbbVie. Alzheimer's Association; Alzheimer's Drug Discovery Foundation; Araclon Biotech; BioClinica. Inc.; Biogen; Bristol-Myers Squibb Company; CereSpir. Inc.; Cogstate; Eisai Inc.; Elan Pharmaceuticals. Inc.; Eli Lilly and Company; EuroImmun; F. Hoffmann-La Roche Ltd and its affiliated company Genentech. Inc.; Fujirebio; GE HealtControlsare; IXICO Ltd.; Janssen Alzheimer Immunotherapy Research & Development. LLC.; Johnson & Johnson Pharmaceutical Research & Development LLC.; Lumosity; Lundbeck; Merck & Co. Inc.; Meso Scale Diagnostics. LLC.; NeuroRx Research; Neurotrack Technologies; Novartis Pharmaceuticals Corporation; Pfizer Inc.; Piramal Imaging; Servier; Takeda Pharmaceutical Company; and Transition Therapeutics. The Canadian Institutes of Health Research is providing funds to support ADNI clinical sites in Canada. Private sector contributions are facilitated by the Foundation for the National Institutes of Health. The grantee organization is the Northern California Institute for Research and Education, and the study is coordinated by the Alzheimer's Therapeutic Research Institute at the University of Southern California. ADNI data are disseminated by the Laboratory for Neuro Imaging at the University of Southern California.

NCRAD

Biological samples used in this study were stored at study investigators' institutions and at the National Cell Repository for Alzheimer's Disease (NCRAD) at Indiana University, which receives government support under a cooperative agreement grant (U24 AG21886) awarded by the National Institute on Aging (NIA). We thank contributors who collected samples used in this study, as well as patients and their families, whose help and participation made this work possible.

UK Biobank

UK Biobank data were analyzed under Application Number 45420.

FinnGen Study

We want to acknowledge the participants and investigators of the FinnGen study"

VA Million Veteran Program: Core Acknowledgement

MVP Program Office

- Sumitra Muralidhar, Ph.D., Program Director

US Department of Veterans Affairs, 810 Vermont Avenue NW, Washington, DC 20420

- Jennifer Moser, Ph.D., Associate Director, Scientific Programs

US Department of Veterans Affairs, 810 Vermont Avenue NW, Washington, DC 20420

- Jennifer E. Deen, B.S., Associate Director, Cohort & Public Relations

US Department of Veterans Affairs, 810 Vermont Avenue NW, Washington, DC 20420

MVP Executive Committee

- Co-Chair: Philip S. Tsao, Ph.D.

VA Palo Alto Health Care System, 3801 Miranda Avenue, Palo Alto, CA 94304

- Co-Chair: Sumitra Muralidhar, Ph.D.

US Department of Veterans Affairs, 810 Vermont Avenue NW, Washington, DC 20420

- J. Michael Gaziano, M.D., M.P.H.

VA Boston Healthcare System, 150 S. Huntington Avenue, Boston, MA 02130

- Elizabeth Hauser, Ph.D.

Durham VA Medical Center, 508 Fulton Street, Durham, NC 27705

- Amy Kilbourne, Ph.D., M.P.H.

VA HSR&D, 2215 Fuller Road, Ann Arbor, MI 48105

- Michael Matheny, M.D., M.S., M.P.H.

VA Tennessee Valley Healthcare System, 1310 24th Ave. South, Nashville, TN 37212

- Dave Oslin, M.D.

Philadelphia VA Medical Center, 3900 Woodland Avenue, Philadelphia, PA 19104

MVP Co-Principal Investigators

- J. Michael Gaziano, M.D., M.P.H.

VA Boston Healthcare System, 150 S. Huntington Avenue, Boston, MA 02130

- Philip S. Tsao, Ph.D.

VA Palo Alto Health Care System, 3801 Miranda Avenue, Palo Alto, CA 94304

MVP Core Operations

- Jessica V. Brewer, M.P.H., Director, Cohort Operations

VA Boston Healthcare System, 150 S. Huntington Avenue, Boston, MA 02130

- Mary T. Brophy M.D., M.P.H., Director, Biorepository

VA Boston Healthcare System, 150 S. Huntington Avenue, Boston, MA 02130

- Kelly Cho, M.P.H, Ph.D., Director, MVP Phenomics

VA Boston Healthcare System, 150 S. Huntington Avenue, Boston, MA 02130

- Lori Churby, B.S., Director, Regulatory Affairs

VA Palo Alto Health Care System, 3801 Miranda Avenue, Palo Alto, CA 94304

- Scott L. DuVall, Ph.D., Director, VA Informatics and Computing Infrastructure (VINCI)

VA Salt Lake City Health Care System, 500 Foothill Drive, Salt Lake City, UT 84148

- Saiju Pyarajan Ph.D., Director, Data and Computational Sciences

VA Boston Healthcare System, 150 S. Huntington Avenue, Boston, MA 02130

- Luis E. Selva, Ph.D., Director, MVP Biorepository Coordination

VA Boston Healthcare System, 150 S. Huntington Avenue, Boston, MA 02130

- Shahpoor (Alex) Shayan, M.S., Director, MVP PRE Informatics

VA Boston Healthcare System, 150 S. Huntington Avenue, Boston, MA 02130

- Stacey B. Whitbourne, Ph.D., Director, MVP Cohort Development and Management

VA Boston Healthcare System, 150 S. Huntington Avenue, Boston, MA 02130

- MVP Coordinating Centers

o MVP Coordinating Center, Boston - J. Michael Gaziano, M.D., M.P.H.

VA Boston Healthcare System, 150 S. Huntington Avenue, Boston, MA 02130

o MVP Coordinating Center, Palo Alto – Philip S. Tsao, Ph.D.

VA Palo Alto Health Care System, 3801 Miranda Avenue, Palo Alto, CA 94304

o MVP Information Center, Canandaigua – Brady Stephens, M.S.

Canandaigua VA Medical Center, 400 Fort Hill Avenue, Canandaigua, NY 14424

o Cooperative Studies Program Clinical Research Pharmacy Coordinating Center, Albuquerque – Todd Connor, Pharm.D.; Dean P. Argyres, B.S., M.S.

New Mexico VA Health Care System, 1501 San Pedro Drive SE, Albuquerque, NM 87108

MVP Publications and Presentations Committee

- Co-Chair: Themistocles L. Assimes, M.D., Ph. D

VA Palo Alto Health Care System, 3801 Miranda Avenue, Palo Alto, CA 94304

- Co-Chair: Adriana Hung, M.D.; M.P.H

VA Tennessee Valley Healthcare System, 1310 24th Ave. South, Nashville, TN 37212

- Co-Chair: Henry Kranzler, M.D.

Philadelphia VA Medical Center, 3900 Woodland Avenue, Philadelphia, PA 19104

MVP Local Site Investigators

- Samuel Aguayo, M.D., Phoenix VA Health Care System

650 E. Indian School Road, Phoenix, AZ 85012

- Sunil Ahuja, M.D., South Texas Veterans Health Care System

7400 Merton Minter Boulevard, San Antonio, TX 78229

- Kathrina Alexander, M.D., Veterans Health Care System of the Ozarks

1100 North College Avenue, Fayetteville, AR 72703

- Xiao M. Androulakis, M.D., Columbia VA Health Care System

6439 Garners Ferry Road, Columbia, SC 29209

- Prakash Balasubramanian, M.D., William S. Middleton Memorial Veterans Hospital

2500 Overlook Terrace, Madison, WI 53705

- Zuhair Ballas, M.D., Iowa City VA Health Care System

601 Highway 6 West, Iowa City, IA 52246-2208

- Elizabeth S. Bast, M.D., M.P.H., Miami VA Health Care System

1201 NW 16th Street, 11 GRC, Miami FL 33125

- Jean Beckham, Ph.D., Durham VA Medical Center

508 Fulton Street, Durham, NC 27705

- Sujata Bhushan, M.D., VA North Texas Health Care System

4500 S. Lancaster Road, Dallas, TX 75216

- Edward Boyko, M.D., VA Puget Sound Health Care System

1660 S. Columbian Way, Seattle, WA 98108-1597

- David Cohen, M.D., Portland VA Medical Center

3710 SW U.S. Veterans Hospital Road, Portland, OR 97239

- Louis Dellitalia, M.D., Birmingham VA Medical Center

700 S. 19th Street, Birmingham AL 35233

- Gerald Wayne Dryden, Jr., M.D., Ph.D., Louisville VA Medical Center

800 Zorn Avenue, Louisville, KY 40206

- L. Christine Faulk, M.D., Robert J. Dole VA Medical Center

5500 East Kellogg Drive, Wichita, KS 67218-1607

- Joseph Fayad, M.D., VA Southern Nevada Healthcare System

6900 North Pecos Road, North Las Vegas, NV 89086

- Daryl Fujii, Ph.D., VA Pacific Islands Health Care System

459 Patterson Rd, Honolulu, HI 96819

- Saib Gappy, M.D., John D. Dingell VA Medical Center

4646 John R Street, Detroit, MI 48201

- Frank Gesek, Ph.D., White River Junction VA Medical Center

163 Veterans Drive, White River Junction, VT 05009

- Michael Godschalk, M.D., Richmond VA Medical Center

1201 Broad Rock Blvd., Richmond, VA 23249

- Jennifer Greco, M.D., Sioux Falls VA Health Care System

2501 W 22nd Street, Sioux Falls, SD 57105

- Todd W. Gress, M.D., Ph.D., Hershel “Woody” Williams VA Medical Center

1540 Spring Valley Drive, Huntington, WV 25704

- Samir Gupta, M.D., M.S.C.S., VA San Diego Healthcare System

3350 La Jolla Village Drive, San Diego, CA 92161

- Salvador Gutierrez, M.D., Edward Hines, Jr. VA Medical Center

5000 South 5th Avenue, Hines, IL 60141

- Mark Hamner, M.D., Ralph H. Johnson VA Medical Center

109 Bee Street, Mental Health Research, Charleston, SC 29401

- John Harley, M.D., Ph.D., Cincinnati VA Medical Center

3200 Vine Street, Cincinnati, OH 45220

- Daniel J. Hogan, M.D., Bay Pines VA Healthcare System

10,000 Bay Pines Blvd Bay Pines, FL 33744

- Adriana Hung, M.D., M.P.H., VA Tennessee Valley Healthcare System

1310 24th Avenue, South Nashville, TN 37212

- Robin Hurley, M.D., W.G. (Bill) Hefner VA Medical Center

1601 Brenner Ave, Salisbury, NC 28144

- Pran Iruvanti, D.O., Ph.D., Hampton VA Medical Center

100 Emancipation Drive, Hampton, VA 23667

- Frank Jacono, M.D., VA Northeast Ohio Healthcare System

10701 East Boulevard, Cleveland, OH 44106

- Darshana Jhala, M.D., Philadelphia VA Medical Center

3900 Woodland Avenue, Philadelphia, PA 19104

- Seema Joshi, M.D., F.A.C.P., ABOIM; VA Eastern Kansas Health Care System

4101 S 4th Street Trafficway, Leavenworth, KS 66048

- Scott Kinlay, M.B.B.S., Ph.D., VA Boston Healthcare System

150 S. Huntington Avenue, Boston, MA 02130

- Michael Landry, Ph.D., Southeast Louisiana Veterans Health Care System

2400 Canal Street, New Orleans, LA 70119

- Peter Liang, M.D., M.P.H., VA New York Harbor Healthcare System

423 East 23rd Street, New York, NY 10010

- Suthat Liangpunsakul, M.D., M.P.H., Richard Roudebush VA Medical Center

1481 West 10th Street, Indianapolis, IN 46202

- Jack Lichy, M.D., Ph.D., Washington DC VA Medical Center

50 Irving St, Washington, D. C. 20422

- Tze Shien Lo, M.D., Fargo VA Health Care System

2101 N. Elm, Fargo, ND 58102

- C. Scott Mahan, M.D., Charles George VA Medical Center

1100 Tunnel Road, Asheville, NC 28805

- Ronnie Marrache, M.D., VA Maine Healthcare System Center, Augusta, ME 04330

- Stephen Mastorides, M.D., James A. Haley Veterans' Hospital

13000 Bruce B. Downs Blvd, Tampa, FL 33612

- Kristin Mattocks, Ph.D., M.P.H., Central Western Massachusetts Healthcare System

421 North Main Street, Leeds, MA 01053

- Paul Meyer, M.D., Ph.D., Southern Arizona VA Health Care System

3601 S 6th Avenue, Tucson, AZ 85723

- Jonathan Moorman, M.D., Ph.D., James H. Quillen VA Medical Center

Corner of Lamont & Veterans Way, Mountain Home, TN 37684

- Providencia Morales, R.N., Northern Arizona VA Health Care System

500 Highway 89 North, Prescott, AZ 86313

- Timothy Morgan, M.D., VA Long Beach Healthcare System

5901 East 7th Street Long Beach, CA 90822

- Maureen Murdoch, M.D., M.P.H., Minneapolis VA Health Care System

One Veterans Drive, Minneapolis, MN 55417

- Eknath Naik, M.D., Ph.D., West Palm Beach VA Medical Center,

7305 North Military Trail, West Palm Beach, FL 33410-6400

- James Norton, Ph.D., VA Health Care Upstate New York

113 Holland Avenue, Albany, NY 12208

- Olaoluwa Okusaga, M.D., Michael E. DeBakey VA Medical Center

2002 Holcombe Blvd, Houston, TX 77030

- Michael K. Ong, M.D., VA Greater Los Angeles Health Care System

11301 Wilshire Blvd, Los Angeles, CA 90073

- Kris Ann Oursler, M.D., Salem VA Medical Center

1970 Roanoke Blvd, Salem, VA 24153

- Ismene Petrakis, M.D., VA Connecticut Healthcare System

950 Campbell Avenue, West Haven, CT 06516

- Samuel Poon, M.D., Manchester VA Medical Center

718 Smyth Road, Manchester, NH 03104

- Amneet S. Rai, Pharm.D., VA Sierra Nevada Health Care System

975 Kirman Avenue, Reno, NV 89502

- Michael Rauchman, M.D., St Louis VA Health Care System

915 North Grand Blvd, St Louis, MO 63106

- Richard Servatius, Ph.D., Syracuse VA Medical Center

800 Irving Avenue, Syracuse, NY 13210

- Satish Sharma, M.D., Providence VA Medical Center

830 Chalkstone Avenue, Providence, RI 02908

- River Smith, Ph.D., Eastern Oklahoma VA Health Care System

1011 Honor Heights Drive, Muskogee, OK 74401

- Peruvemba Sriram, M.D., N. FL/S. GA Veterans Health System

1601 SW Archer Road, Gainesville, FL 32608

- Patrick Strollo, Jr., M.D., VA Pittsburgh Health Care System

University Drive, Pittsburgh, PA 15240

- Neeraj Tandon, M.D., Overton Brooks VA Medical Center

510 East Stoner Ave, Shreveport, LA 71101

- Philip Tsao, Ph.D., VA Palo Alto Health Care System

3801 Miranda Avenue, Palo Alto, CA 94304-1290

- Gerardo Villareal, M.D., New Mexico VA Health Care System

1501 San Pedro Drive, S.E. Albuquerque, NM 87108

- Jessica Walsh, M.D., VA Salt Lake City Health Care System

500 Foothill Drive, Salt Lake City, UT 84148

- John Wells, Ph.D., Edith Nourse Rogers Memorial Veterans Hospital

200 Springs Road, Bedford, MA 01730

- Jeffrey Whittle, M.D., M.P.H., Clement J. Zablocki VA Medical Center

5000 West National Avenue, Milwaukee, WI 53295

- Mary Whooley, M.D., San Francisco VA Health Care System

4150 Clement Street, San Francisco, CA 94121

- Peter Wilson, M.D., Atlanta VA Medical Center

1670 Clairmont Road, Decatur, GA 30033

- Junzhe Xu, M.D., VA Western New York Healthcare System

3495 Bailey Avenue, Buffalo, NY 14215-1199

- Shing Shing Yeh, Ph.D., M.D., Northport VA Medical Center

79 Middleville Road, Northport, NY 11768

- Andrew W. Yen, M.D., VA Northern California Health Care System

10535 Hospital Way, Mather, CA 95655
