# Supplementary material for: Role of the X Chromosome in Alzheimer Disease Genetics
Source: JAMA Neurol. 2024 Sep 9;81(10):1032–42. doi: 10.1001/jamaneurol.2024.2843 (PMC11385320; doi:10.1001/jamaneurol.2024.2843)
Supplement: Supplement 2. — Data sharing statement [file jamaneurol-e242843-s002.pdf]

# Data Sharing Statement

Belloy. Role of the X Chromosome in Alzheimer Disease Genetics. *JAMA Neurol*. Published September 03, 2024. doi:10.1001/jamaneurol.2024.2843

**Data**
**Data available:** No

**Additional Information**
**Explanation for why data not available:** All summary statistics from the genetic meta-analyses are available in the GWAS catalogue (GCP000979). We additionally provide all data pointer and access locations for raw data, to which other researchers can apply for access. However, we are not at liberty to release the raw data ourselves.
